# Distinct changes in the morphology of cortical and subcortical grey matter associated with age-related hearing loss and tinnitus in the UK Biobank participants

**DOI:** 10.1093/braincomms/fcaf203

**Published:** 2025-05-27

**Authors:** Fatin N Zainul Abidin, Francesca Biondo, Andre Altmann, Sally J Dawson

**Affiliations:** UCL Ear Institute, University College London, London WC1X 8EE, UK; UCL Hawkes Institute, Department of Medical Physics and Biomedical Engineering, University College London, London WC1V 6BH, UK; UCL Hawkes Institute, Department of Medical Physics and Biomedical Engineering, University College London, London WC1V 6BH, UK; UCL Hawkes Institute, Department of Medical Physics and Biomedical Engineering, University College London, London WC1V 6BH, UK; UCL Ear Institute, University College London, London WC1X 8EE, UK

**Keywords:** age-related hearing loss, tinnitus, neuroimaging, thalamus, Heschl’s gyrus

## Abstract

Prevalence of both hearing loss and tinnitus increases with age. However, neuroimaging studies of both conditions report inconsistent changes in brain morphology likely due to small sample size and variable methodology. Structural and functional neuroimaging studies in hearing loss and tinnitus have revealed distinct neural correlates, and further replication is needed to confirm these findings. This study aims to investigate the effects of hearing loss and tinnitus on the brain morphology in a well-powered sample. We utilized self-reported hearing difficulty and tinnitus in participants with magnetic resonance imaging (MRI) in the UK Biobank cohort. Control participants without hearing difficulty and tinnitus were age and sex matched leading to total sample sizes of 13 074 and 6242 for self-reported hearing difficulty and tinnitus, respectively. We utilized the rich UK Biobank dataset (i) to reveal these brain changes in a well-powered large study of hearing loss and tinnitus, (ii) to document the effect of confounding factors on these associations, (iii) to discriminate the effects of tinnitus versus hearing difficulty on the brain and (iv) to estimate the brain-age gap in hearing difficulty and tinnitus subjects compared with controls. Hearing difficulty is significantly associated with smaller grey matter volumes exclusively in the bilateral transverse temporal regions, whereas tinnitus is associated with larger volumes of bilateral hippocampi and thalami when compared with the control group. Furthermore, correcting for confounders (i.e. diabetes, cardiovascular disease, age, sex, smoking, alcohol consumption and Townsend deprivation index) during statistical analysis helped to better delineate the impact of hearing status on brain structural changes. The brain-age gap analysis showed that participants with tinnitus appeared to have significantly younger brains than controls, whereas participants with hearing difficulty did not differ significantly from the control group. Altogether, our results confirmed previous findings and suggest the enlargement of bilateral thalami as the main effect in people with tinnitus. We also established that there are independent and distinct brain pathologies between hearing difficulty and tinnitus. Therefore, the self-reported measure is a reasonable approach to assess the hearing loss and tinnitus pathologies.

## Teaser

Using UK Biobank dataset, Zainul Abidin *et al*. report that hearing difficulty and tinnitus patients possess different brain structural characteristics where Heschl’s gyri and thalami are significantly associated with hearing difficulty and tinnitus respectively. In hearing difficulty, Heschl’s gyri are reduced in volume, whereas thalami are increased in volume.

## Graphical Abstract

**Graphical Abstract:**
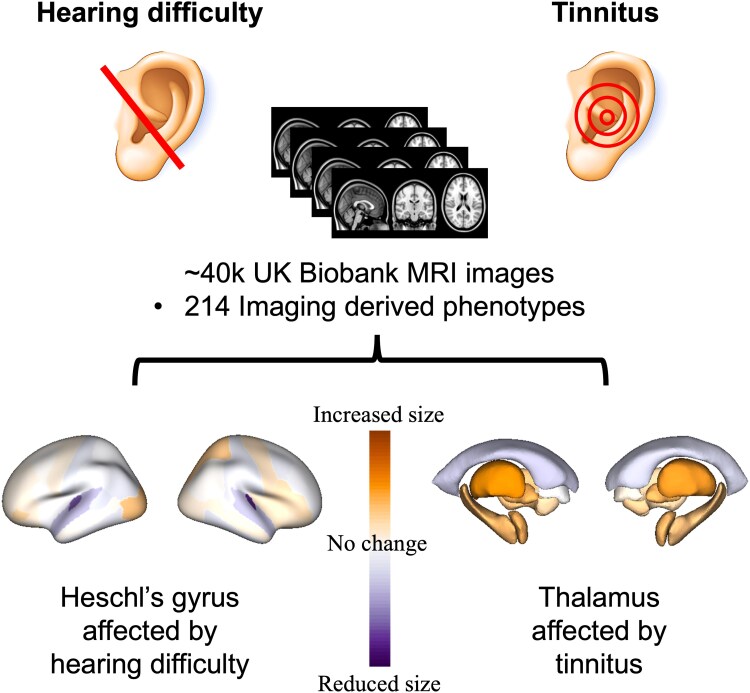

## Introduction

Age-related hearing loss or presbycusis is the gradual loss of hearing with increasing age and is the single biggest cause of hearing loss. According to the World Health Organization, 400 million adults worldwide currently experience disabling hearing loss. By 2050, this number is projected to rise to 700 million or one in 10 people.^1^ Age-related hearing loss is characterized by reduced hearing sensitivity in both ears, impaired localization of sound sources, decreased ability to understand speech in background noise and slowed central processing of acoustic input.^2^ Age-related hearing loss leads to depression and social isolation. Hearing loss in midlife is one of the most significant modifiable risk factors for dementia, alongside hypertension and obesity. Early intervention could potentially delay or decrease the risk of developing dementia in later life.^3^

Hearing loss is often accompanied by tinnitus (ringing in the ears without an acoustic stimulus) and prevalence of both hearing loss and tinnitus increases with age.^4-6^ Moreover, the prevalence of tinnitus is known to increase with worsening of hearing status.^7^ Since most individuals with tinnitus already have hearing loss, it is challenging to distinguish the effects caused by hearing loss from those caused by tinnitus. Previous studies have suggested that hearing loss, tinnitus and mental health problems are commonly associated.^8-10^ Thus, it is important to detect and treat these conditions. Recent genetic studies have found hearing loss and tinnitus to have distinct genetic architecture.^8,11,12^ Meta-analyses between hearing difficulty and tinnitus genome-wide association studies have indicated only 40.7% of variants influencing tinnitus are shared with hearing difficulty, suggesting tinnitus as a distinct disorder separate from hearing difficulty.^8^

Structural brain MRI studies that provide information on the shape, size and integrity of brain structures also suggest independent neural correlates of hearing loss and tinnitus.^13-15^ Hearing loss has also been shown to be linked to quicker shrinkage of areas of the brain responsible for processing sounds^16^ and memories.^17^ However, to date, most of these studies on brain structural changes in hearing impairment or tinnitus have been of small scale (<200 subjects), restricted to a few selected regions of interest, and these data have not been adjusted for hearing loss or tinnitus risk factors^13-18^ (see Supplementary Table 1), thus producing variable results between studies. Previous studies have reported increased prevalence of hearing loss and/or tinnitus in people with diabetes,^19,20^ cardiovascular disease risk factors^20-22^ and lifestyle factors^14^ (i.e. insomnia, income and Townsend index).^14^ However, each risk factor may independently also affect brain morphology. Therefore, to isolate the effect of hearing loss or tinnitus, statistical analyses should account for these risk factors as covariates.

We utilized the rich UK Biobank (UKBB) dataset to (i) reveal what these brain changes are in well-powered large study of hearing loss and tinnitus, (ii) document the effect of confounding factors on these associations, (iii) discriminate the effects of tinnitus versus hearing difficulty on the brain and (iv) estimated the brain-age gap (BAG) in hearing difficulty and tinnitus subjects. In these analyses, we investigated the effects of known confounders on the association of affected brain regions linked to either condition. In addition, we hypothesized that hearing loss and tinnitus may affect overall brain health. Therefore, we investigated the different effect on age prediction from brain imaging, also known as ‘brain age’,^23^ between participants with hearing difficulty and tinnitus compared with healthy controls. The ‘BAG’, which is the difference between brain age and chronological age, has been established as a useful personalized biomarker of brain health.^24^ Our findings provide insight in the context of the established effects of hearing loss and tinnitus on depression, cognitive decline and mental health.

## Materials and methods

### Participants and phenotype definition

The study utilized a sample comprising individuals who took part in the UKBB study,^25^ a nationwide initiative initially established to investigate the impact of lifestyle and genetic factors on aging traits. Its primary goal is to enhance understanding and promote healthy aging at a population level. Between 2007 and 2013, more than 500 000 volunteers attended 23 assessment centres across the UK, where they contributed samples for genotyping, completed lifestyle questionnaires and underwent standard measurements. All participants provided informed consent to join the UKBB, and ethical approval was obtained from the Northwest Multi-Centre Ethics Committee. The conducted experiments adhered to these guidelines and regulations, following the ethical governance and framework established by the UKBB (https://www.ukbiobank.ac.uk/ethics/). The research was carried out using the UKBB Resource under application numbers 11516 and 41127.

Questions regarding hearing difficulty and tinnitus were included in the UKBB ‘Health and medical history’ questionnaire that participants completed on touchscreen monitors while attending an assessment centre at the same imaging visit (Instance 2) when MRI was undertaken. As described previously,^11^ cases for hearing difficulty were defined as having answered ‘Yes’ to both questions: ‘Do you have any difficulty with your hearing?’ and ‘Do you find it difficult to follow a conversation if there is background noise (such as TV, radio, children playing)?’. Questions concerning the use of hearing aid were not considered for inclusion into the case cohort. Controls were selected if their response to both questions was ‘No’. Participants with any other combination of responses were removed. Participants were removed from the control group if they answered ‘Yes’ to ‘Do you use a hearing aid most of the time?’. Cases for tinnitus were defined as previously described^12^ based on responses to the question ‘Do you get, or have you had noises (such as ringing or buzzing in your head or in one or both ears that lasts for more than 5 min at a time)?’. Participants who responded either ‘Yes, now most or all of the time’ or ‘Yes, now a lot of the time’ were assigned ‘cases’ and those that responded ‘No’ were assigned controls. This study focused on individuals who reported frequent tinnitus. Therefore, following previous work,^12^ people who answered ‘Yes, now some of the time’ or ‘Yes, but not now, but have in the past’ were neither included in the case nor in the control group for tinnitus.

**Figure 1:**
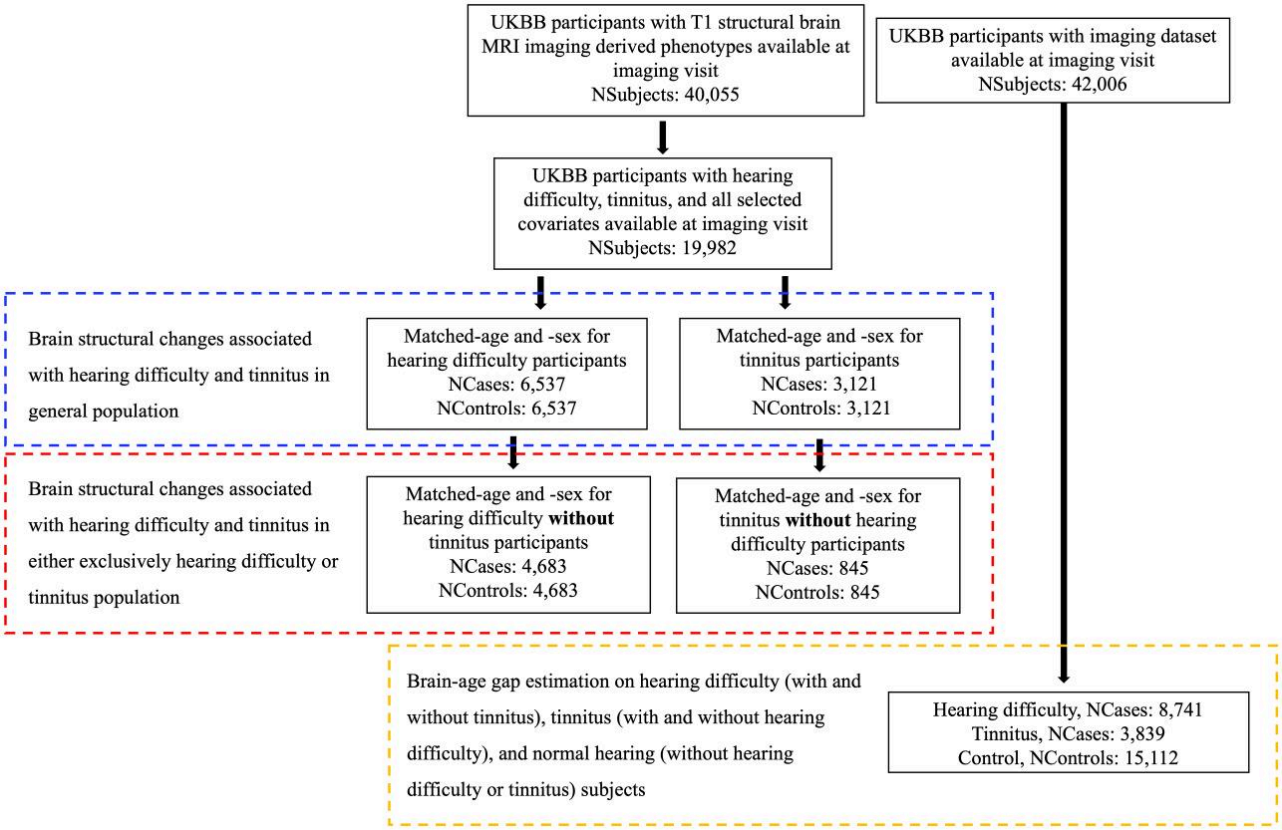
Flow chart describing case-control assignments and sample sizes for the hearing difficulty and tinnitus phenotypes and brain-age estimation study.

For this analysis, we used the UKBB ethnic group White British for case and control groups participants (Supplementary Table 2). Case and control groups were also matched for both age and sex in both hearing difficulty and tinnitus analyses (see Fig. 1). Individuals were included according to the availability of MRI and covariate data that include cardiovascular disease, diabetes, alcohol consumption status, smoking status, Townsend deprivation index and body mass index (BMI) (Supplementary Table 2). These covariates were chosen based on their association with hearing impairment in previous studies.^20,26-28^ In addition, we also investigated a sub-population of people with tinnitus but no hearing difficulty (854 cases and 854 controls with age and sex matched) and people with hearing difficulty but no tinnitus (4863 cases and 4863 controls with age and sex matched) (see Fig. 1). Matching by demographics (age and sex) and further including demographic variables as covariates in our statistical analysis helped to minimize their effect on the investigated group differences.

### Imaging-derived phenotypes

At the time of analysis (March 2023), there were 40 055 participants with processed data from MRI in UKBB at the imaging visit (from year 2014 to March 2023). T1-weighted MRI is a structural technique with high-resolution depiction of brain anatomy, having strong contrast between grey and white matter, reflecting differences in the interaction of water with surrounding tissues. To facilitate high-throughput processing of brain images, an automated image analysis pipeline was also developed,^29^ permitting rapid and reproducible image analysis of large datasets. The pipeline also extracts imaging-derived phenotypes (IDPs): quantitative measures relating to area, mean thickness and volumes of cortical and subcortical brain structures that can be easily used and interpreted by non-imaging experts. Structural MRI was processed using Freesurfer^30^; the Desikan-Killiany^31^ and the automatically segmented brain volume (ASEG)^32^ atlases were used to segment the cortex and subcortex, respectively. Details of the acquisition protocols, image processing pipeline, image data files and derived measures IDPs of brain structure can be found online (https://biobank.ctsu.ox.ac.uk/crystal/crystal/docs/brain_mri.pdf). In total, 198 cortical IDPs (66 regions for both hemispheres including surface area, mean thickness and volume) and 16 subcortical IDPs (volumes for 8 regions for both hemispheres)^20,33^ (see Supplementary Fig. 1) were included in this study.

### Statistical analysis

Differences between selected key demographics and health outcomes between the two case-control groups (either hearing difficulty versus no hearing difficulty or tinnitus versus no tinnitus) were analysed in R using Student’s *t*-test for continuous variables and the $\chi^{2}$ test for categorical variables. Multiple logistic regression models with various levels of covariate adjustments were used to test the associations between hearing difficulty and tinnitus groups and brain structures or IDPs (see Supplementary Tables 3 and 4). In the primary analysis, the outcome measures were from each of 198 cortical and 16 subcortical IDPs. For each IDP, a separate model was fitted with effects:

**Regression Model 1**: IDP ∼ hearing difficulty/tinnitus status + age + sex + intracranial volume.

**Regression Model 2**: IDP ∼ hearing difficulty/tinnitus status + age + sex + intracranial volume + further covariates.

Regression Model 1 tested the effect of hearing difficulty or tinnitus on brain structures in case versus control groups adjusting for the known confounds of the IDPs, which were age, sex and intracranial volume.^34^ Regression Model 2 extends Regression Model 1 by additionally adjusting the comparison for further covariates including BMI, cardiovascular disease, diabetes, drinking, Townsend deprivation index and smoking status. The rationale of Regression Model 2 over Regression Model 1 is to account for further confounding factors that are associated with hearing difficulty or tinnitus and that also may influence the IDP. This way the effect of hearing difficulty and tinnitus on brain morphology is further isolated. Multiple tests were conducted across all IDPs for surface area, mean cortical thickness and volume in each regression model (214 tests) for hearing loss and tinnitus. In addition, we also studied the brain changes on hearing difficulty alone without tinnitus and vice versa (Supplementary Tables 5 and 6). Due to the dependence between tests, the *P*-values were adjusted for multiple testing using the Benjamini and Hochberg^35^ method for false discovery rate (FDR) correction, and an adjusted *P*-value of <0.05 was considered as statistically significant. Maps of the *t*-statistics for cortical and subcortical analyses for both hearing difficulty and tinnitus were visualized using the ENIGMA toolbox.^36^

### Brain-age gap estimation on hearing difficulty and tinnitus subjects

Brain-age models use regression methods to predict a subject’s age from brain imaging data. Increased brain age compared with chronological age indicates accelerated brain aging.^23^ Brain age prediction has previously been used to evaluate the effects of interactions of genes, environment, life burden and diseases on the pace of brain aging^37^ and has been successfully applied in multiple sclerosis,^38^ Alzheimer’s disease, dementia^39,40^ and major depressive disorder.^41^ We used brain age as a biomarker that reflects cognitive decline and that can be analysed in relation to hearing difficulty or tinnitus status. To estimate participants’ brain age, we followed the processing and feature extraction adopted by pyBrainAge (https://github.com/james-cole/PyBrainAge). However, because pyBrainAge relied extensively on UKBB participants for model training, it could not be applied to reliably estimate brain age for our study cohort, which comprises only UKBB participants. In machine learning, the application of the trained model to its training data results in overly optimistic estimates. Thus, for valid estimates, machine learning models, including brain-age predictions, must always be applied to out-of-sample data, i.e. examples that have not been used during training. To circumvent this problem, we estimated brain age on UKBB data using train test splits and cross-validation. Briefly, the model utilized neuroimaging features including cortical thickness based on the Destrieux atlas parcellations^42^ as well as subcortical volumes both extracted using FreeSurfer,^30^ resulting in a total of 187 input features. The full list of imaging features is available here: https://github.com/james-cole/PyBrainAge/blob/main/ROIS_input_template.txt. There were 42 006 subjects with imaging data available (date accessed: 10/07/2023). A total of 14 947 subjects with known neurological and health issues were excluded from the training data and formed the ‘case’ cohort. The remaining 27 059 subjects were randomly split into 80% training (*N* = 21 642) and 20% testing (*N* = 5410).

Next, the brain-age model using elastic net regression was trained in R (version 4.1.0) using the glmnet package (version 4.1-7). In this model, the participants’ age at imaging was the target variable and all the 187 MRI-derived features were the predictor variables. For the elastic net regression, the trade-off between L1 and L2 penalty was set to 0.5 and the strength of regularization (i.e. lambda) was optimized using 10-fold cross-validation on the training data using the inbuilt cv.glmnet() function. The lambda parameter that minimized the mean absolute error between age at imaging and predicted age at imaging was selected. Next, the model was re-trained on the full training data using the optimized lambda parameter and brain age was predicted for the test set as well as the ‘case’ cohort. For the training set, we used the brain-age prediction obtained during the cross-validation. This enabled us to obtain a predicted brain age for all 42 006 participants. The BAG was defined as the difference between the predicted brain age and the actual age at imaging. Positive values of BAG indicate older-appearing brains while negative values of BAG indicated younger-appearing brains. However, due to the common regression to the mean phenomenon, where the predicted age for every subject will be shrunk towards the mean age of the training sample,^43^ we computed the predicted BAG (pBAG). To this end, we regressed the actual age against the BAG estimate with a simple linear regression and used the residual as the pBAG. To ensure that the predictions on the training set from cross-validation and the out-of-sample predictions (e.g. for the test set and the ‘case’ cohort) were comparable, we compared the pBAG for the training set (based on cross-validation) and the test set using a *t*-test.

## Results

### Characteristics of designated case-control groups

Applying our definition of hearing difficulty and tinnitus resulted in 13 074 and 6242 subjects, respectively, with a complete set of MRI and covariates (Fig. 1). The final experimental sample groups comprise 6537 participants with hearing difficulty and 3121 participants with tinnitus as well as corresponding age- and sex-matched control groups. As expected, a large proportion of participants with tinnitus (73%) also experience hearing difficulty (see Table 1). Conversely, only 28% of participants with hearing difficulty also experience tinnitus. In addition, 24% of hearing difficulty and 20% tinnitus subjects are wearing hearing aids. Hearing difficulty and tinnitus were both strongly associated with known covariates such as socioeconomic status (Townsend deprivation index), smoking, cardiovascular health, diabetes and BMI, but not with alcohol intake (Table 1). There were no associations with age and sex, indicating successful matching.

**Table 1:** Characteristics of hearing difficulty and tinnitus participants at imaging visit

	Hearing difficulty			Tinnitus		
Characteristic	Cases (*N* = 6537)	Controls (*N* = 6537)	*P*-value	Cases (*N* = 3121)	Controls (*N* = 3121)	*P*-value
Hdiff						
No	NA	NA	NA	854 (27.36%)	3121 (100%)	<2.2e^−16^
Yes	NA	NA		2267 (72.64%)	0 (0%)	
Tinnitus						
No	4683 (71.64%)	6537 (100%)	<2.2e^−16^	NA	NA	NA
Yes	1854 (28.36%)	0 (0%)		NA	NA	
Haid						
No	4966 (75.97%)	6537 (100%)	<2.2e^−16^	2483(79.56%)	3121(100%)	<2.2e^−16^
Yes	1563 (23.91%)	0 (0%)		633 (20.28%)	0	
Age (years)	65.44 (6.97)	65.44 (6.97)	1	65.09 (7.02)	65.09 (7.02)	1
Sex						
Female	3483 (53.28%)	3483(53.28%)	1	1816 (58.19%)	1816 (58.19%)	1
Male	3054 (46.72%)	3054 (46.72%)		1305 (41.81%)	1305 (41.81%)	
BMI	26.49 (4.30)	24.20 (3.28)	<2.2e^−16^	26.78 (4.37)	22.35 (2.15)	<2.2e^−16^
Smoking						
No	3954 (60.49%)	4316 (66.02%)	5.803e^−11^	1826 (58.51%)	2134 (68.38%)	7.114e^−16^
Yes	2583 (39.51%)	2221 (33.98%)		1295 (41.49%)	987 (31.62%)	
Drinking						
No	187 (2.86%)	179 (2.74%)	0.7105	94 (3.01%)	91 (2.92%)	0.8813
Yes	6350 (97.14%)	6358 (97.26%)		3027 (96.99%)	3030 (97.08%)	
CVD						
No	4665 (71.36%)	5033 (76.99%)	2.237e^−13^	2191 (70.20%)	2560 (82.02%)	<2.2e^−16^
Yes	1872 (28.64%)	1504 (23.01%)		930 (29.80%)	561 (17.98%)	
Diabetes						
No	6230 (95.30%)	6333 (96.88%)	4.163e^−06^	2958 (94.78%)	3062 (98.11%)	1.933e^−12^
Yes	307 (4.70%)	204 (3.12%)		163 (5.22%)	59 (1.89%)	
TDI	−2.06 (2.59)	−2.24 (2.54)	4.812e^−05^	−1.97 (2.66)	−2.22 (2.57)	7.402e^−05^

Data are shown as mean (standard deviation) for continuous variables and as number (percentage) for categorical variables. *P*-values were analysed using Student's *t*-test for continuous variables and the *χ*^2^ test for categorical variables.

BMI, body mass index; CVD, cardiovascular disease; Haid, hearing aid; Hdiff, hearing difficulty; NA, not applicable; TDI, Townsend deprivation index.

### Brain structural changes associated with hearing difficulty and tinnitus

**Figure 2:**
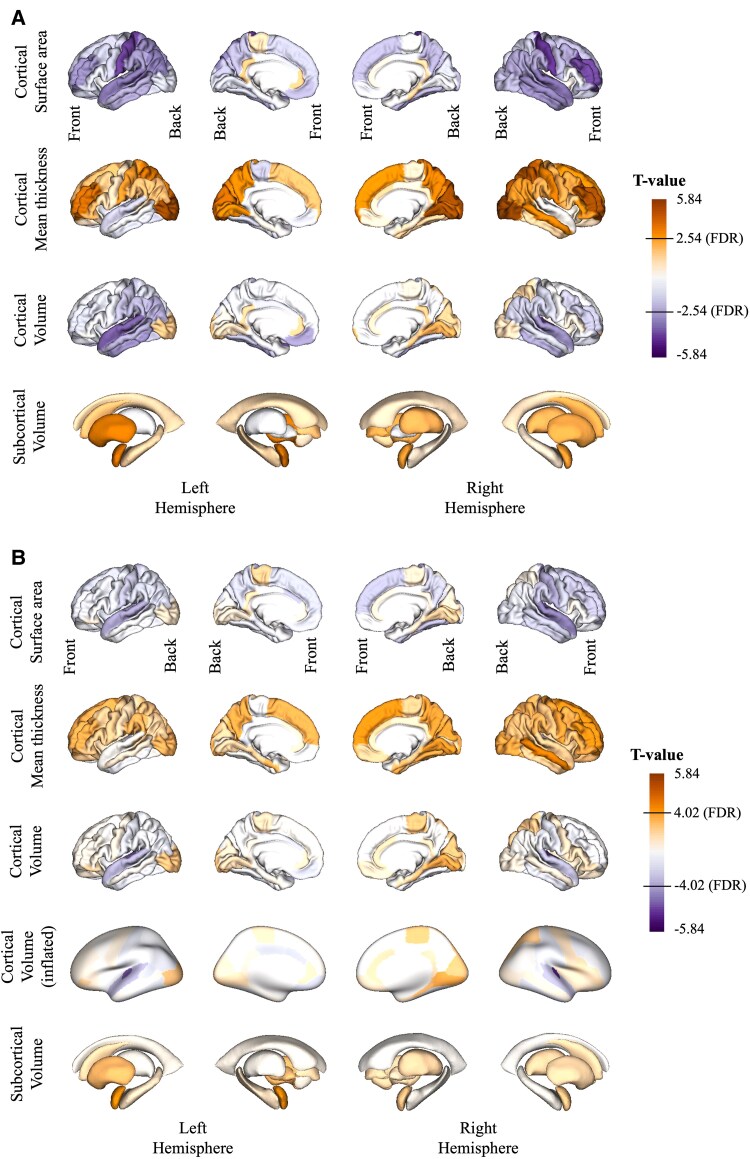
**The association between hearing difficulty and brain structure detected in structural T1-weighted MRI.** This association was investigated in the multiple linear regression models (*N*_cases_ = 6537 and *N*_controls_ = 6537) with (**A**) Regression Model 1 without additional covariates and (**B**) Regression Model 2 with additional covariates added. (B) Additional row showing the inflated cortex representation to better highlight the Heschl’s gyri. Colours relate to the *T*-value of the effect sizes from both multiple linear regression models collectively ranging from −5.84 to 5.84. Negative and positive effect sizes indicate cortical/subcortical thinning and thickening, respectively, in hearing difficulty cases compared with controls. FDR, false discovery rate.

**Figure 3:**
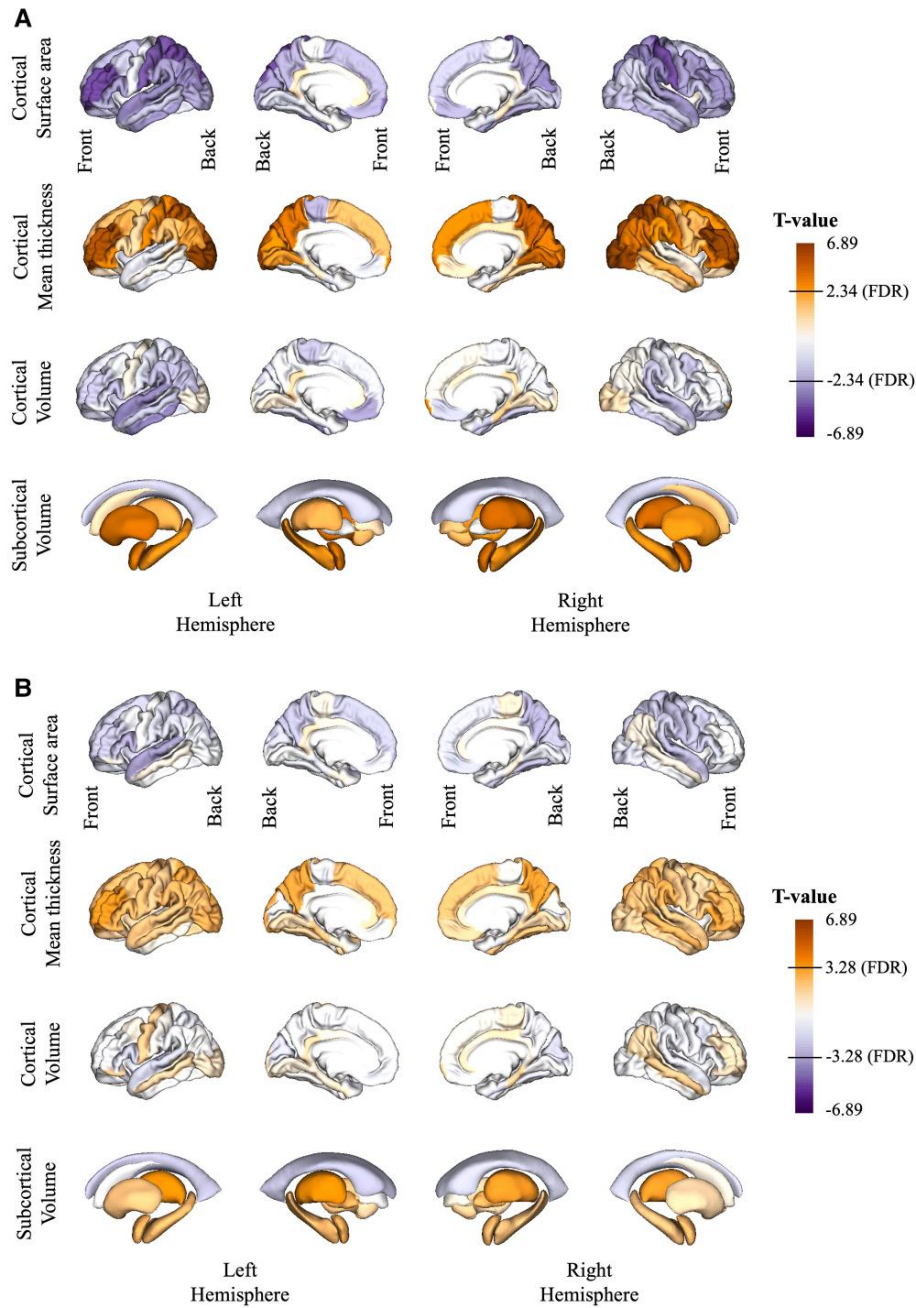
**The association between tinnitus and brain structure detected in structural T1-weighted MRI.** This association was investigated in the multiple linear regression models (*N*_cases_ = 3121 and *N*_controls_ = 3121) with (**A**) Regression Model 1 without additional covariate and (**B**) Regression Model 2 with additional covariates. Colours relate to the *T*-value of the effect sizes from both multiple linear regression models collectively ranging from −6.89 to 6.89. Negative and positive effect sizes indicate cortical/subcortical thinning and thickening respectively in tinnitus cases compared with controls. FDR, false discovery rate.

We investigated the effects of self-reported hearing loss and tinnitus on brain morphology using linear regression models. In Model 1, we adjusted for standard covariates sex, age and intracranial volume only (Materials and methods). In the hearing difficulty analyses, we predominantly observed shrinkage in surface area (most prominent in transverse temporal area) and also observed increases in cortical thickness (most prominent in occipital area and the frontal lobe) (Fig. 2A and Supplementary Table 3). Overall, the net effect on cortical volume is negative (most prominent in the temporal lobe and the Heschl’s gyri) with the occipital lobe being the exception. In the subcortical areas, we observed increases of thalamus and amygdala in the left hemisphere (Fig. 2A and Supplementary Table 3). Overall, for hearing difficulty, the strongest effect sizes were observed for the mean thicknesses of the lateral occipital regions (Model 1; left *T* = 5.35; *P*_FDR_ = 7.92e^−06^ and right *T* = 5.84; *P*_FDR_ = 1.17e^−06^). In Model 2 (Materials and methods) when we adjusted for additional confounders, these effects largely disappeared (Fig. 2B and Supplementary Table 3). The strongest remaining effect sizes under this model were observed for volumes of the bilateral Heschl’s gyri (Model 2; left *T* = −4.02; *P*_FDR_ = 4.77e^−03^ and right *T* = −4.99; *P*_FDR_ = 1.27e^−04^). These are the only regions to survive multiple testing correction in both analyses (Fig. 2, Supplementary Fig. 2 and Supplementary Table 3). In a second analysis investigating the effects of tinnitus status, a large number of regions were associated with tinnitus with the strongest effect sizes being detected for mean thicknesses in lateral occipital regions (Model 1; left *T* = 6.87; *P*_FDR_ = 7.51e^−10^ and right *T* = 6.89; *P*_FDR_ = 7.51e^−10^) (Fig. 3 and Supplementary Table 4). After additional covariates correction in Model 2, effect sizes of tinnitus on the volumes of left and right thalamus were the strongest (Model 2; left *T* = 3.77; *P*_FDR_ = 2.65e^−02^ and right *T* = 3.53; *P*_FDR_ = 2.65e^−02^). Other regions that survived FDR multiple testing correction include increasing mean thicknesses of both left and right precuneus, mean thicknesses of left rostral middle frontal, left superior parietal and right parstriangularis (see Fig. 3, Supplementary Fig. 3 and Supplementary Table 4).

### Distinct and shared effects of hearing difficulty and tinnitus on brain structure

**Figure 4:**
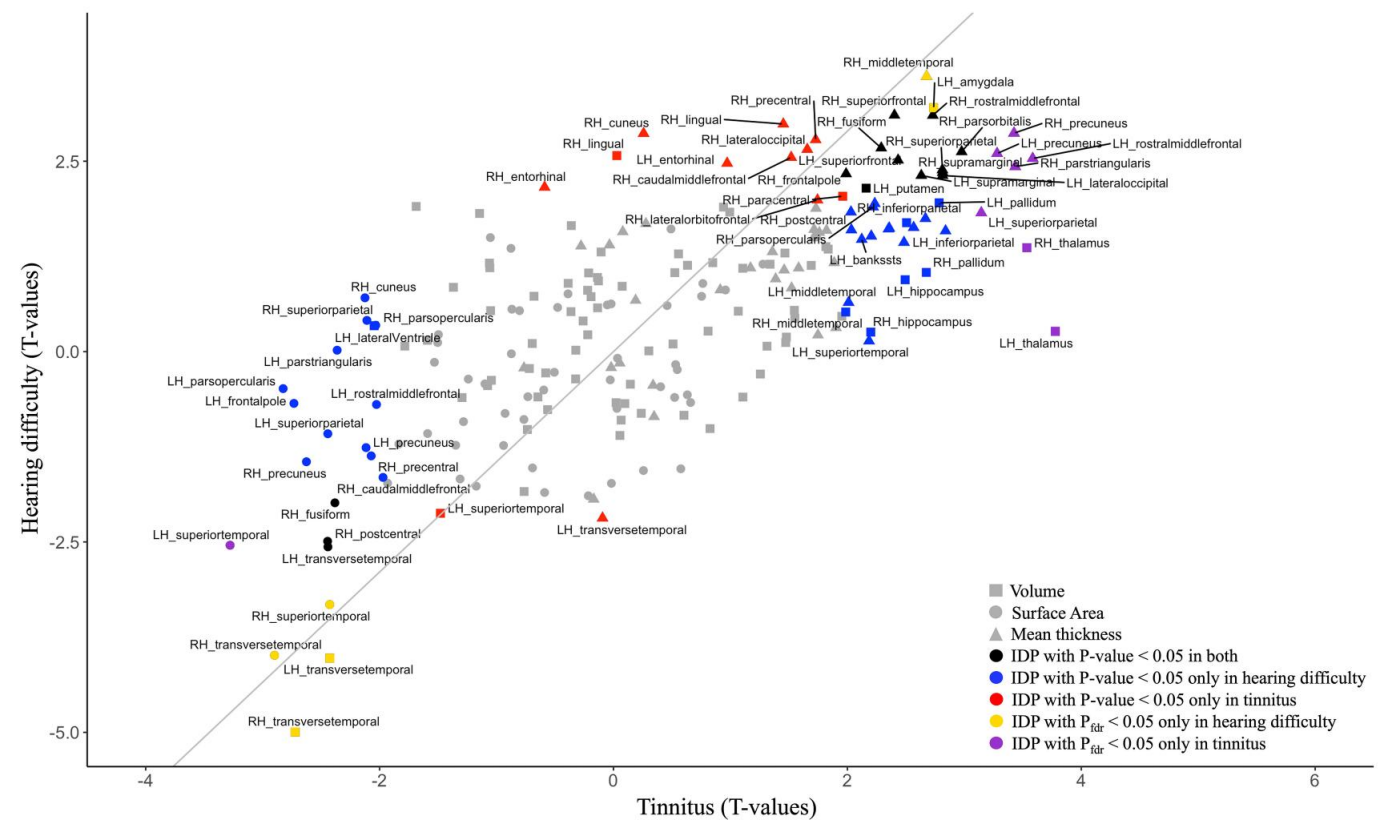
**Plot comparing *T*-values from hearing difficulty and tinnitus linear regression (Model 2).** Analyses adjusted for age, sex, intracranial volume and further health-related covariates. Each data point represents IDP in two different conditions. The straight line represents the expected association between *T*-values for hearing difficulty (*N*_total_ = 13 074) and tinnitus (*N*_total_ = 6242) from mixed effect linear Model 2 after accounting for sample size differences (reference line: intercept = 0, slope = sqrt(13 047/6242). FDR, false discovery rate; *P*_FDR_, FDR-corrected *P*-value; IDP, imaging-derived phenotype; LH, left hemisphere; RH, right hemisphere.

In the analyses of brain structural changes in relation to hearing difficulty or tinnitus, we observe shared effects. For example, temporal regions (i.e. transverse temporal and superior temporal) and subcortical regions (i.e. hippocampus, amygdala, thalamus and pallidum) were reduced and increased in size, respectively, in both conditions (Fig. 4). Regions that were associated specifically with hearing difficulty at *P*_FDR_ < 0.05 include reduced volumes of bilateral transverse temporal gyrus and the area of the right superior temporal gyrus, increased thickness of the right middle temporal gyrus and increased volume of the left amygdala (see Fig. 4 for significant regions and Supplementary Table 3 for full cortical effects). Meanwhile, in tinnitus, specific changes were observed in the volumes of the bilateral thalamus, volume of the left pallidum, mean thickness of the bilateral precuneus and the right parstriangularis were increased compared with healthy controls (*P*_FDR_ < 0.05; see Fig. 4 for significant regions and Supplementary Table 4 for full cortical effects). Effects associated with hearing difficulty only at a nominal *P*-value threshold of <0.05 that did not survive multiple testing correction are mean thicknesses in right cuneus, right lingual, lateral occipital, bilateral entorhinal and volumes in the right lingual and left superior temporal gyrus were enlarged in hearing difficulty and not significant in tinnitus. Reduced areas in bilateral cuneus, bilateral parsopercularis, bilateral superior parietal, left frontal pole, right caudal middle frontal, right paracentral and volumes in left lateral ventricle were found to be associated with tinnitus and not hearing difficulty at a nominal *P*-value of <0.05. Meanwhile, several subcortical regions were found increased in volumes in bilateral amygdala, hippocampus, pallidum and thalamus at *P* < 0.05 (see Fig. 4 for significant regions and Supplementary Tables 3 and 4 for full cortical effects). We have confirmed the thalamus enlargement in tinnitus-only subjects at *P* < 0.05 (Supplementary Table 6).

### Brain age is more strongly related to tinnitus than hearing loss

**Figure 5:**
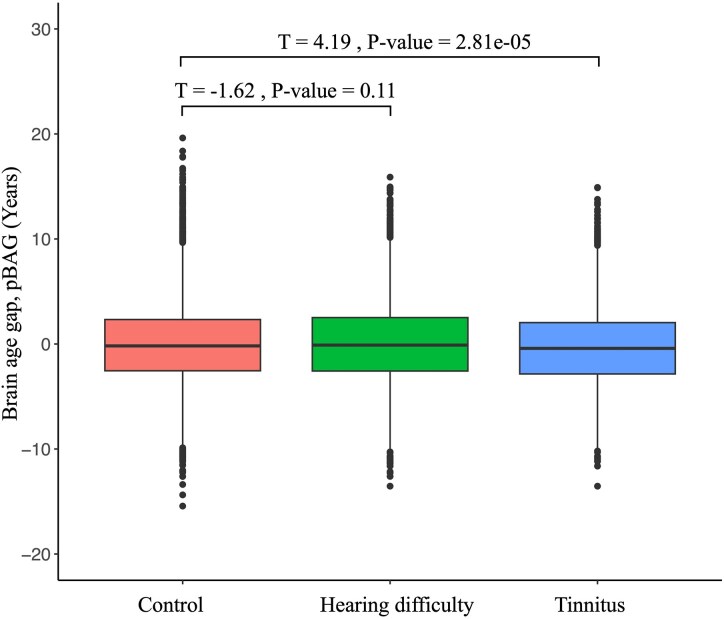
**Boxplots on the comparisons of pBAG between control, hearing difficulty and tinnitus groups.** The boxplot depicting the pBAG (predicted brain age—chronological age) for each group (control, hearing difficulty and tinnitus). Welch’s two-sample *T*-tests were used between hearing difficulty and tinnitus with control group separately. Number of subjects in control (*N* = 15 112), hearing difficulty with and without tinnitus (*N* = 8741) and tinnitus with and without hearing difficulty (*N* = 3839) groups. pBAG, predicted brain-age gap.

We attained a mean absolute difference of 4.01 years between predicted age and chronological age for our brain-age model (Supplementary Fig. 4). There was no statistically significant difference between pBAG in the test set and the cross-validation prediction (*T*-test; *T* = 0.44, *P* = 0.66) suggesting that these pBAG estimates can be analysed jointly. Following this, we have included 15 112 controls (without hearing loss and tinnitus), 8741 participants with hearing difficulty and 3839 participants with tinnitus in the brain-age study. We found that participants with tinnitus have predicted brain age significantly younger than controls (negative pBAG value, Cohen’s *D* = 0.08, *T* = 4.19, *P* = 2.81e^−05^; Fig. 5) whereas participants with hearing difficulty have a predicted brain age that is not significantly different to the control group (Cohen’s *D* = 0.02, *T* = −1.62, *P* = 0.11; Fig. 5).

## Discussion

In this study, we utilized the self-reported hearing difficulty and tinnitus data in UKBB cohort to study the brain changes detected in MRI brain images in these groups. We controlled for the known effects of aging and sex on hearing loss and tinnitus by ensuring age and sex matching between cases and controls and then adjusting for these factors in our regression Models 1 and 2. Besides age and sex, we identified cardiovascular disease, diabetes, socioeconomic status, smoking and BMI as factors associated with hearing loss and tinnitus, and these associations are consistent with previous studies.^20,21,26,27^ However, alcohol consumption did not show any association with either hearing loss or tinnitus. This lack of association with alcohol consumption may be attributed to the high proportion of active alcohol drinkers among participants recruited from the UKBB, comprising 97% of the total participants. Thus, variables reflecting a more granular quantification of alcohol consumption may shed further light onto this association.

By implementing additional covariate corrections beyond the typical adjustments for age, sex and intracranial volume, we were able to identify more specific regions than in Model 1 using only basic covariate adjustment. In our Model 2 for hearing difficulty, several significant effects on temporal regions detected in Model 1 disappeared and only bilateral Heschl’s gyri association remained. This highlights the importance of accounting for relevant covariates, or confounders, in all imaging studies of disease. Our previous work combining functional imaging (i.e. positron emission tomography) and self-reported hearing impairment showed reduced glucose metabolism in the bilateral Heschl’s gyri, inferior colliculus and cochlear nucleus.^16^ The analysis in this work, using structural MRI, confirms a loss of grey matter in the bilateral Heschl’s gyri (also referred to as transverse temporal gyri). These results are consistent with other audiometry test-based studies’ findings on Heschl’s gyrus effects.^15,44^ Moreover, decline of cortical grey matter in the brain’s primary auditory processing regions parallels findings in people in people with vision problems who exhibit reduced sizes of the occipital lobe or the primary visual cortex.^45-47^ Interestingly, in our study, cortical thickness in the occipital regions appeared larger in the hearing difficulty group compared with controls, suggesting that these regions may compensate for diminished auditory input by engaging more in visual and linguistic processing. Additional correction of covariates in Model 2 also had similar effects on associations with tinnitus, where bilateral thalamus volumes and precuneus mean thicknesses remained significantly affected, while many other regions previously identified using Model 1 vanished.

Our study features a considerably larger sample size (*N*_total_ = 13 074 and *N*_total_ = 6242 for hearing difficulty and tinnitus, respectively) in comparison with prior research focusing on objective hearing tests among individuals experiencing hearing loss and self-reported tinnitus (these are summarized in Supplementary Table 1). Additionally, these studies often did not appropriately account for additional covariates.^13,15,18-48^ In recent years, the availability of the UKBB cohort has led to increased sample sizes for investigating the effects of hearing loss and tinnitus on the brain. For instance, the work by Chen *et al.*^14^ comprised over 5000 participants. However, due to different phenotype definition, exclusions and MRI availability at that time, only 311 had hearing loss, 720 had tinnitus, and 117 had both. Notably, Wang *et al.*^20^ included all available participants with brain imaging data in UKBB (*N* = 38 438). Besides age and sex, their study also adjusted for a few additional sociodemographic variables. They reported widespread reduction of volume in cortical and subcortical regions, most prominently in the temporal lobe. However, their analysis used the speech-in-noise hearing test, which a previous study did not consider suitable due to lack of clear heritability or association with age.^11^ Moreover, although their analysis covaries for age and sex, they did not match cases and controls based on these covariates; therefore, the results may have been confounded. Our Model 1 agrees with their findings of reduced volume in the temporal lobe of people with hearing loss (Fig. 2A). However, after adjusting for additional confounders, including cardiovascular disease, most associations disappeared leaving the bilateral transverse temporal gyri with the strongest signal.

In our research, we observed that 73% of individuals with tinnitus also have hearing difficulty, while only 28% of those with hearing difficulties experience tinnitus. This is consistent with many previous studies that have found damaged hearing is strongly linked to tinnitus. Tinnitus has been proposed to be a central auditory response to peripheral cochlea dysfunction although the mechanism has not been established.^49,50^ Here, we have found the bilateral brain regions mostly affected by tinnitus are in the precuneus and subcortical brain region of thalamus. Compared with the effects observed with hearing difficulty, which shows volume reductions primarily in the bilateral Heschl’s gyrus, these regions show a notable expansion in tinnitus. Our Model 1 shows the enlargement in a number of subcortical regions such as amygdala, hippocampus, putamen, accumbens and pallidum, which have previously been reported.^13-15^ However, these are not significant in Model 2 suggesting these enlargements could be driven by covariates such as BMI or diabetes in general. Previous studies^13-15^ have not adjusted for these covariates; consequently, these regions may not be tinnitus specific, whereas our finding on thalamus and precuneus remain significant after adjusting for covariates should be tinnitus specific. The strongest expansion observed in the bilateral thalami, which acts as a relay centre, directing sensory information from the periphery including auditory signals to other non-auditory subcortical and cortical brain regions for processing. The observed expansion was more than twice the expansion observed in hearing difficulty compared with controls. Thus, the thalamus appears to play an important role in tinnitus and supports findings by Mühlau *et al.*^51^ in a much smaller cohort (28 had tinnitus with no hearing loss). Similarly, bilateral precuneus mean thicknesses also appeared larger in the tinnitus group. The precuneus is a highly integrated structure and a hub of the brain’s default mode network. As such, the region is involved in functions such as self-consciousness and shifting of attention. Previous work has established closer links between the precuneus and tinnitus, including auditory memory retrieval, auditory imagery and memory-related aspects of the tinnitus perception.^52,53^ Moreover, the precuneus was also found to play a significant role in tinnitus-related distress.^54-56^

While the thalami, along with other brain regions, typically experience volume reduction and structural alterations as individuals age, our observations suggest the contrary in people with tinnitus. Similar observations were found in tinnitus functional MRI and structural MRI studies where non-auditory brain regions were showing generalized aberrant neural activity^57^ and cortical volume enlargement.^58^ Generally, tinnitus can result in anxiety and depression, which frequently induce alterations in the morphology of limbic structures.^59,60^ Lateral ventricles were reduced in volume, likely due to the expansion of other nearby subcortical structures.

Lastly, in our brain-age analysis, individuals with tinnitus exhibited a younger predicted brain age compared with healthy controls (Fig. 5). This is not in the expected direction given that typically negative health outcomes are associated to older looking brains,^61-64^ while younger looking brains were associated with positive health outcomes (e.g. higher levels of exercise and meditation).^65,66^ This inverse direction, although small (Cohen’s *D* = 0.08), may be attributed to the overall enlargement of multiple brain regions in participants with tinnitus when compared with controls (Fig. 3). Conversely, there was no difference between controls and participants with hearing difficulty (Fig. 5). The lack of association may be due to the way this brain-age model was trained: although participants with neurodegenerative disease and severe health issues were excluded from the training set, subjects with hearing difficulty were considered normal and retained in the training data. Thus, the subtle effect of hearing difficulty on brain morphology may be considered a normal part of aging in this model. Overall, this analysis serves also as a cautionary tale: some common conditions change the brain in the opposite direction of the expected aging effect. In hearing difficulty, the cortical thickness for the occipital regions increases (makes the brain look younger), and in tinnitus, we see multiple regions increasing (e.g. thalamus, precuneus and hippocampus) (see Fig. 4), in this case, leading to a significantly lower BAG than in controls.

A limitation in our study lies in the fact that this study relied on region-based analyses rather than voxel-based analyses. Thus, our study provides the basis for future studies on voxel level for tinnitus and hearing loss researchers which we would expect to pinpoint the same general brain regions but likely home in on much smaller targeted sub-region. A large portion of the participants with hearing difficulty (24%) or tinnitus (20%) reported the use of hearing aids. Our analyses did not include hearing aid use as a covariate; thus, potential protective effects may have reduced the observed effects of hearing impairment on brain morphology. The role of hearing aid use as possible intervention to protect brain health will require further longitudinal analyses of the UKBB cohort in future. Furthermore, we used self-reported hearing and tinnitus data rather than audiometric measurements. However, our findings from this and previous studies^11,12,16^ provide evidence to confidently conclude that questionnaires on hearing difficulty and tinnitus are meaningful in terms of genetics as well as brain changes that accompany these conditions. To conclude, through these self-reported measures, we were able to establish the bilateral thalami as the main effect of tinnitus and independent and distinct brain pathologies between tinnitus and hearing difficulty. Therefore, the self-reported measure is a reasonable approach to assess the hearing loss and tinnitus pathologies.

## Supplementary Material

fcaf203_Supplementary_Data

## Data Availability

Imaging, clinical and demographic data from UK Biobank were downloaded under the application 11516 and 41127 and can be accessed via their standard data access procedure (see http://www.ukbiobank.ac.uk/register-apply).
