# Supplementary material for: Distinct changes in the morphology of cortical and subcortical grey matter associated with age-related hearing loss and tinnitus in the UK Biobank participants
Source: Brain Commun. 2025 May 27;7(3):fcaf203. doi: 10.1093/braincomms/fcaf203 (PMC12149739; doi:10.1093/braincomms/fcaf203)
Supplement: fcaf203_Supplementary_Data [file fcaf203_supplementary_data.pdf]

Distinct changes in the morphology of cortical and subcortical grey matter associated with age-related hearing loss and tinnitus in the UK Biobank participants

Fatin N Zainul Abidin[1,2], Francesca Biondo[2], Andre Altmann[2,†], Sally J Dawson[1,†]

[†] These authors contributed equally to this work.

1 UCL Ear Institute, University College London, London, WC1X 8EE, United Kingdom
2 UCL Hawkes Institute, Department of Medical Physics and Biomedical Engineering, University College London, London, WC1V 6BH, United Kingdom

Correspondence to: Dr. Andre Altmann and Prof Sally Dawson
Full address: UCL Hawkes Institute, Department of Medical Physics and Biomedical Engineering, University College London, London, WC1V 6BH, United Kingdom
Email: a.altmann@ucl.ac.uk, sally.dawson@ucl.ac.uk

Supplementary Figures Page

Supplementary Figure 1 …………………………………………………………..………...…3
Imaging derived phenotypes (IDPs) of brain regions used in our analyses
Supplementary Figure 2 …………………………………………………………..………...…4
High contrast colour scheme for the association between hearing difficulty and brain structure detected in structural T1-weighted MRI
Supplementary Figure 3……………………………………………………………….………..…5
High contrast colour scheme for the association between tinnitus and brain structure detected in structural T1-weighted MRI.
Supplementary Figure 4……………………………………………………………….………..…6
Scatter plots between chronological age at imaging (x-axis) and predicted age from brain imaging (y-axis)

Supplementary Tables Page

Supplementary Table 1 ………………………………………………………..……..…...…7
A summary of previous studies assessing the association between hearing impairment, tinnitus and structural MRI

Supplementary Table 2 ……………………………………………………….……..…...…10
UKBB categories and descriptions used as covariates for model two
Supplementary Table 3 ………………………………………………………..………...…11
The association between hearing difficulty and brain surface area, mean thickness and volume in the UK Biobank.
Supplementary Table 4 ………………………………………………………..……..…....16
The association between tinnitus and brain surface area, mean thickness and volume in the UK Biobank
Supplementary Table 5 ………………………………………………………..……..…....21
The association between hearing difficulty without tinnitus (hearing difficulty only), mean thickness and volume in the UK Biobank.
Supplementary Table 6 ………………………………………………………..……..……26
The association between tinnitus without hearing loss (tinnitus only), mean thickness and volume in the UK Biobank.

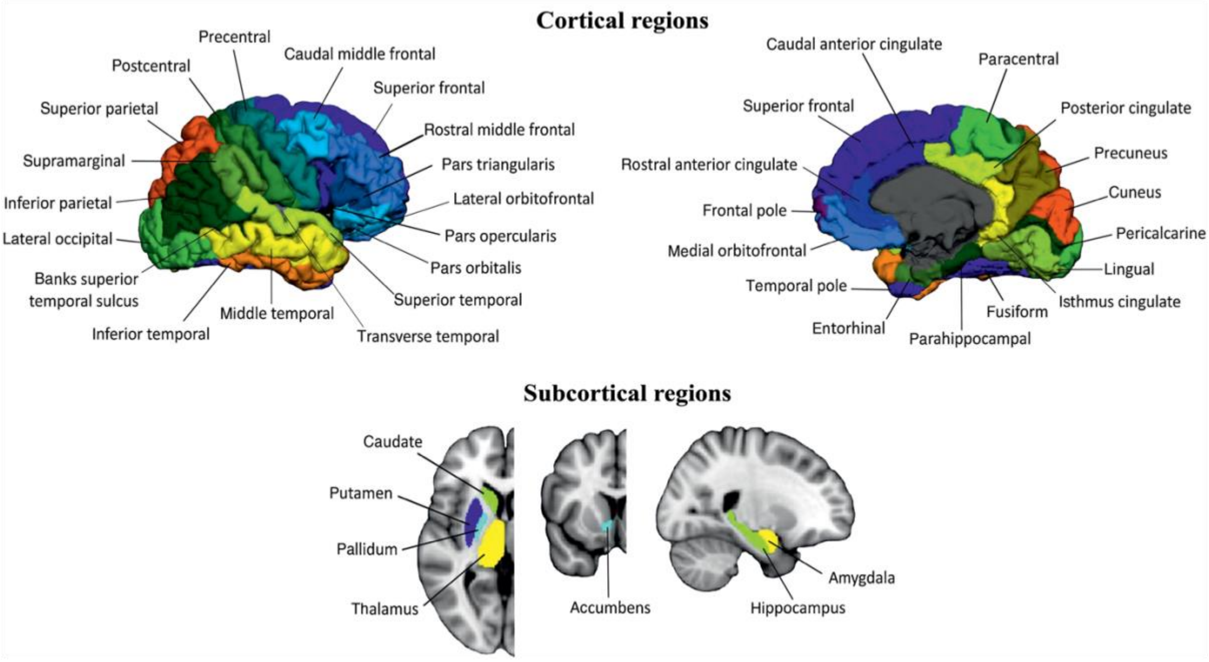

Supplementary Figure 1: Imaging derived phenotypes (IDPs) of brain regions used in our analyses. The cortical surface area, mean thickness and subcortical volumes were derived from these regions for every imaging subject in the UK Biobank. The figure was originally produced and published by Pereira *et al.*[1] and is reproduced without changes under the Creative Commons Attribution 4.0 International License (https://creativecommons.org/licenses/by/4.0/).

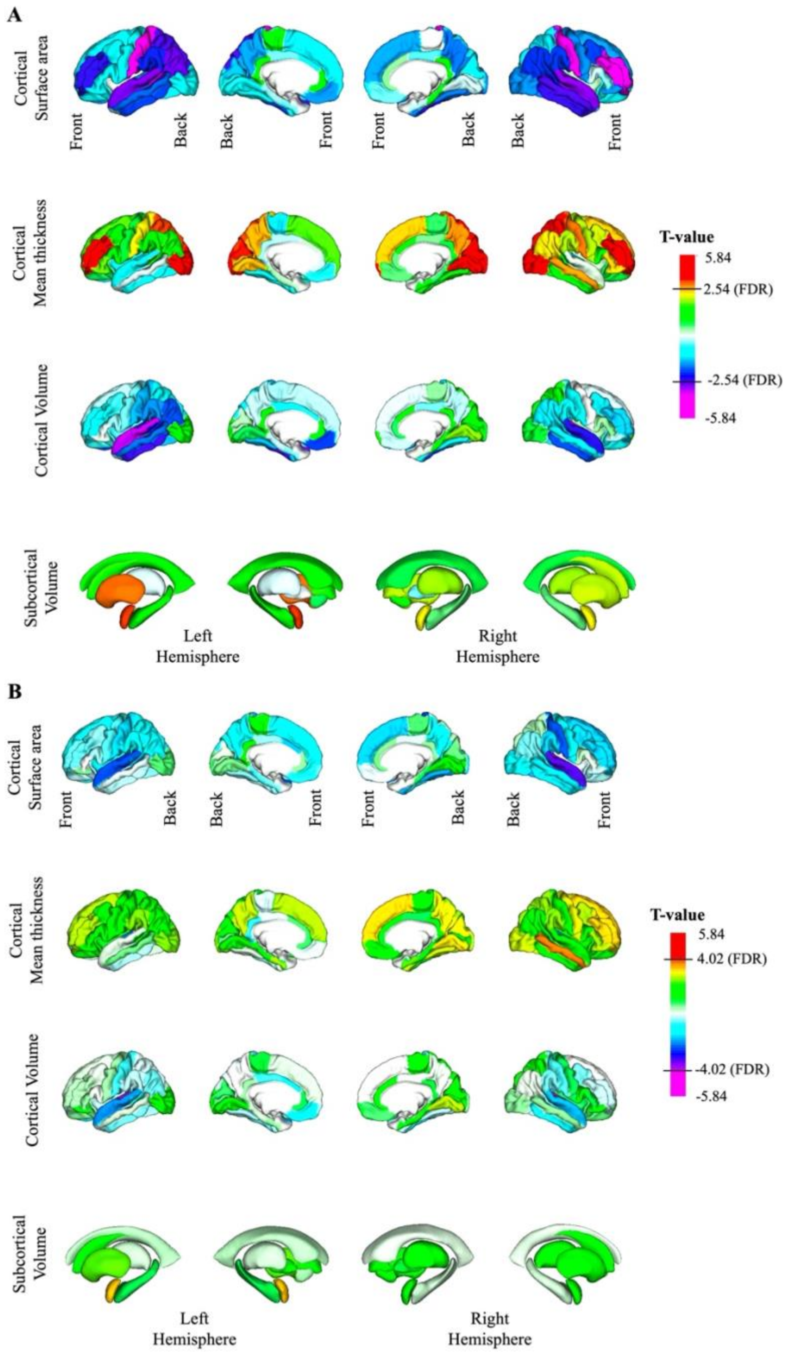

Supplementary Figure 2: High contrast colour scheme for the association between hearing difficulty and brain structure detected in structural T1-weighted MRI. This association was investigated in the multiple linear regression (MLR) models (N_{cases} =6,537 and N_{controls}=6,537) with **A)** regression model 1 without additional covariates and **B)** regression model 2 with additional covariates added. Colours relate to the T-value of the effect sizes from both MLR models collectively ranging from -5.84 to 5.84. Negative and positive effect sizes indicate cortical/subcortical thinning and thickening, respectively, in hearing difficulty cases compared to controls. FDR, false discovery rate corrected *P-value*.

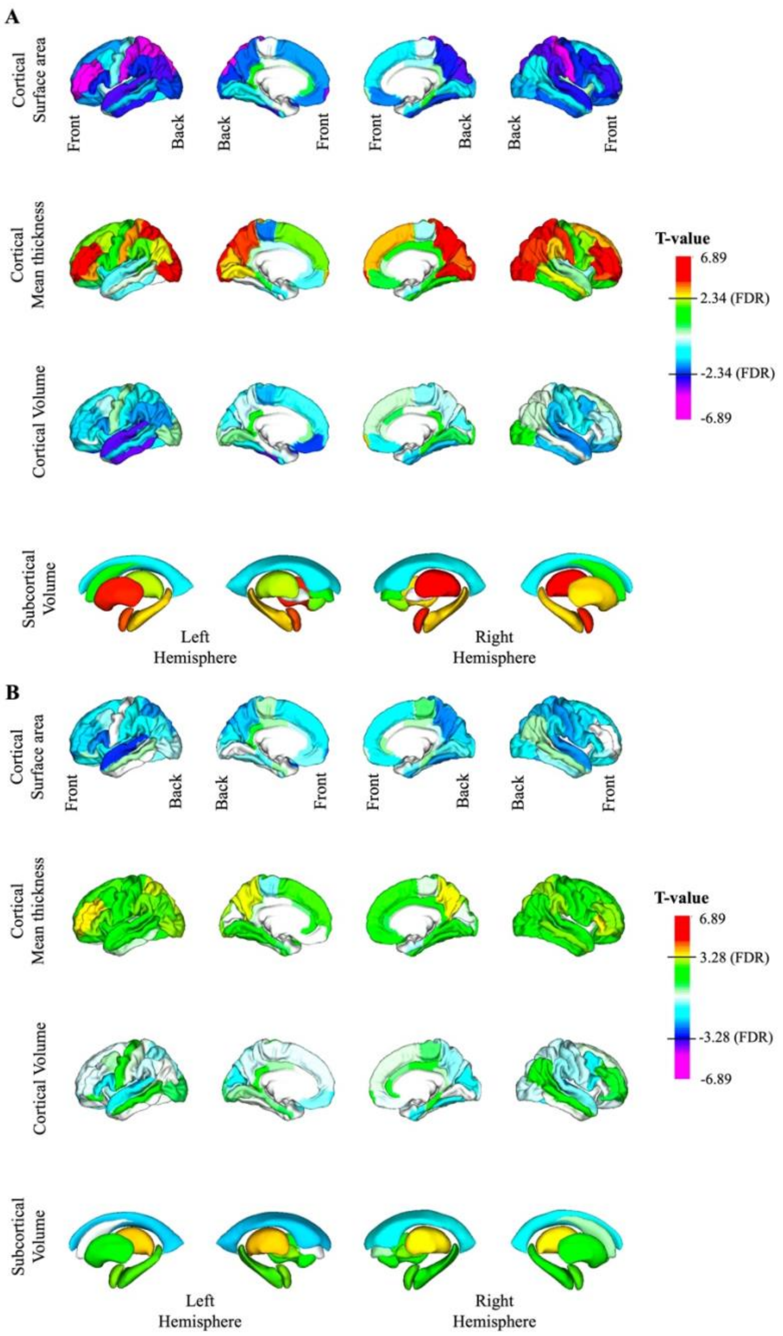

Supplementary Figure 3: High contrast colour scheme for the association between tinnitus and brain structure detected in structural T1-weighted MRI. This association was investigated in the multiple linear regression (MLR) models (N_{cases} =3,121 and *N*controls =3,121) with A) regression model 1 without additional covariate and B) regression model 2 with additional covariates. Colours relate to the *T-value* the effect sizes from both MLR models collectively ranging from -6.89 to 6.89. Negative and positive effect sizes indicate cortical/subcortical thinning and thickening respectively in tinnitus cases compared to controls. FDR, false discovery rate corrected *P-value.*

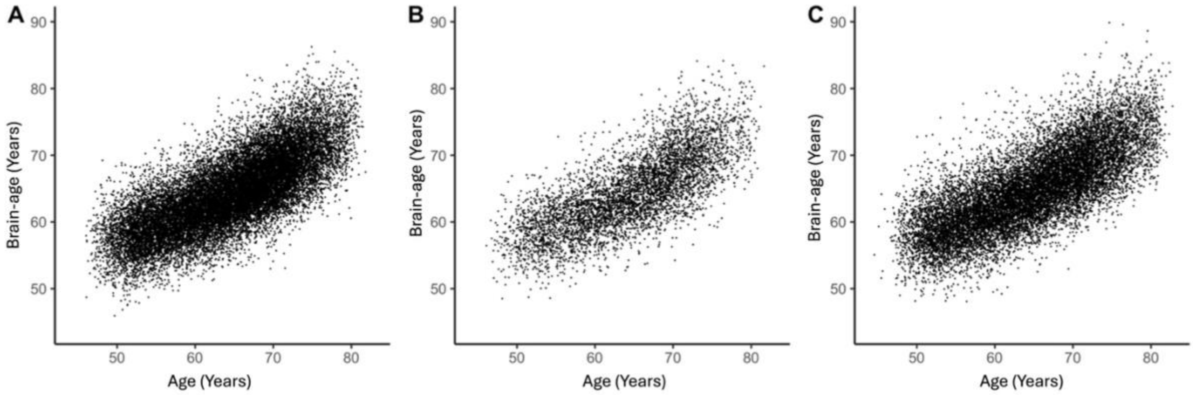

Supplementary Figure 4: Scatter plots between chronological age at imaging (x-axis) and predicted age from brain imaging (y-axis). (**A**) Predictions obtained from cross-validation on the training data (*N*=21,642; mean absolute difference (MAD) =4.01 years; Pearson's r^2 =0.555). (**B**) Predictions for the independent test data of healthy subjects (*N* =5,410; MAD =3.98 years; Pearson's r^2 =0.554). (**C**) Predictions for the independent 'case' cohort with recorded neurological and health issues (*N* =14,947; MAD =4.22 years; Pearson's r^2 =0.564).

Supplementary Table 1. A summary of previous studies assessing the association between hearing impairment, tinnitus and structural MRI. Regions are statistically significant regions reported as the main result by authors of the corresponding paper. Pc, P-value after multiple testing correction either using Bonferroni or FDR method; P, nominal P-value.

**Hearing phenotype**	**Criteria**	***N***	**Associated regions**	**Author, year, link**
Hearing impairment and tinnitus *UK Biobank study*	Self-reported hearing difficulty<br>Self-reported tinnitus	6,537<br>3,121	Hearing difficulty reduces volume of bilateral transverse temporal gyrus (left and right) Pc <0.05<br>Tinnitus increases volume of bilateral thalamus (left and right), area and mean thickness of superior parietal (left), mean thickness of bilateral precuneus (left and right), mean thickness of rostral middle frontal and superior parietal (left), and mean thickness of parstriangularis (right). Pc <0.05	This study
Hearing impairment *The Baltimore Longitudinal Study of Ageing*	Pure tone audiometry	194	Midlife hearing impairment in the right ear was associated with steeper volumetric declines in the temporal grey matter (right), hippocampus (right) and entorhinal cortex (right). Pc <0.05	Armstrong *et al.* (2019)[2]
Hearing loss and tinnitus *UK Biobank study*	Speech reception threshold, Self-reported tinnitus	5,222	None for hearing loss after adjustment for intracranial volume. Pc <0.05<br>Tinnitus increases volume of accumbens (left) and occipital pole (right). Pc <0.05	Chen *et al.* (2024)[3]
Hearing loss and tinnitus	Pure tone audiometry, Tinnitus handicap inventory	73	Hearing loss decreases volume of Heschl's gyrus. P <0.05<br>Tinnitus increases volume of hippocampus and amygdala (right). P <0.05<br>Tinnitus has no effect on the structure of auditory cortex.	Profant *et al.* (2020)[4]

Hearing performance *UK Biobank study*	Speech-in-noise test	38,438	Poor hearing performance reduces volumes of bilateral superior, middle, and inferior temporal gyrus, hippocampus, precuneus, inferior parietal lobe, supramarginal gyrus, fusiform, orbitofrontal cortex, transverse temporal gyrus (left), rostral anterior cingulate (left), rostral middle frontal gyrus (right).<br>Pc <0.05<br>Poor hearing performance associated with lower volumes of amygdala bilaterally, thalamus and nucleus accumbens. Pc <0.05	Wang *et al.* (2022)[5]
Tinnitus with mild-moderate hearing impairment	Pure tone audiometry, Supra-threshold hearing, Speech-in-noise test, Tinnitus questionnaire, Tinnitus handicap inventory	66	Tinnitus increases in cortical volume and surface area of the supramarginal gyrus (right), posterior plannum temporale, surface area middle-anterior of superior temporal sulcus. P <0.05<br>Tinnitus also increases volumes in the amygdala (left) and hippocampus (left). P <0.05	Elmer *et al.* (2023)[6]
Hearing impairment	Pure tone audiometry	126	Hearing impairment accelerates volume declines of whole brain, superior, middle, and inferior temporal gyri (right). P <0.05<br>No significant changes in baseline/cross-sectional analysis.	Lin *et al.* (2014)[7]
Hearing impairment *ADNI study*	Self-reported hearing related keywords	725	In AD group, hearing loss decreases volume of left and right cerebellum white matter, brainstem, ventral diencephalon (left), cerebellar cortex (left and right).<br>P <0.05	Llano *et al.* (2021)[8]

Tinnitus	Self-reported tinnitus with no hearing loss	56	Tinnitus increases gray-matter volume only at thalamic level. Pc <0.05<br>Tinnitus decreases the gray-matter volume in the subcallosal region including nucleus accumbens. Pc <0.05	Muhlau *et al.* (2005)[9]
Tinnitus	Self-reported tinnitus (present/absent)	2,616	Tinnitus increases white matter volumes in each lobe, not in the gray matter volume. P <0.05<br>Voxel-based did not show regional specificity.	Oosterloo *et al.* (2021)[10]
Tinnitus in semantic dementia	Tinnitus handicap inventory	44	Tinnitus preserves grey matter in the posterior superior temporal lobe and reduces grey matter in the orbitofrontal cortex and medial geniculate nucleus. Pc <0.05	Mahoney *et al.* (2011)[11]

Supplementary Table 2: UKBB categories and descriptions used as covariates for model two.

UKBB data field	Category	Description
22006	Genetic ethnic grouping	Indicates samples who self-identified as 'White British' according to Field 21000 and have very similar genetic ancestry based on a principal components analysis of the genotypes.
20002	Non-cancer illness code, self-reported	Hypertension, heart/cardiac problem, cerebrovascular disease, peripheral vascular disease, venous thromboembolic disease.<br>Gestational Diabetes, type 1 Diabetes, type 2 Diabetes.
20117	Alcohol drinker status	Never, Previous, current
20116	Smoking status	Never, Previous, current
22189	Townsend deprivation index at recruitment	Townsend deprivation index calculated immediately prior to participant joining UK Biobank. Based on the preceding national census output areas. Each participant is assigned a score corresponding to the output area in which their postcode is located.
21001	Body mass index (BMI)	BMI value here is constructed from height and weight measured during the initial Assessment Centre visit. Value is not present if either of these readings were omitted.

Supplementary Table 3: The association between hearing difficulty and brain surface area, mean thickness and volume in the UK Biobank. Values highlighted in red are the significant values at respective P-value and corrected P-value at 0.005. ICV: intracranial volume; CVD: cardiovascular disease; BMI: body mass index; TDI, Townsend deprivation index; ROI: region of interest; IDP: imaging derived phenotypes; CI: confidence interval; FDR: false discovery rate.

Cases (6,537) vs. Controls (6,537)	IDP	Model 1: IDP ~ Hdiff + Age + Sex + ICV						Model 2: IDP ~ Hdiff + Age + Sex + ICV + Smoking + Alcohol + Diabetes + CVD + BMI + TDI					
		Estimate	T-value	P-value	Lower-CI	Upper-CI	FDR	Estimate	T-value	P-value	Lower-CI	Upper-CI	FDR
Cortical Surface Area	L_bankssts	-9.090	-3.747	1.79E-04	-13.844	-4.336	2.11E-03	-4.805	-1.893	5.83E-02	-9.779	0.169	2.84E-01
	L_caudalanteriorcingulate	-2.418	-1.127	2.60E-01	-6.623	1.786	4.21E-01	-4.159	-1.850	6.43E-02	-8.564	0.246	2.92E-01
	L_caudalmiddlefrontal	-7.232	-1.496	1.35E-01	-16.704	2.240	2.74E-01	-2.552	-0.504	6.14E-01	-12.475	7.372	7.87E-01
	L_cuneus	-6.431	-1.832	6.70E-02	-13.312	0.450	1.67E-01	0.819	0.223	8.23E-01	-6.379	8.018	9.05E-01
	L_entorhinal	-1.079	-0.712	4.77E-01	-4.050	1.892	6.14E-01	-1.061	-0.668	5.04E-01	-4.174	2.051	7.34E-01
	L_frontalpole	-0.716	-1.581	1.14E-01	-1.604	0.172	2.47E-01	-0.323	-0.680	4.96E-01	-1.253	0.608	7.34E-01
	L_fusiform	-5.922	-1.237	2.16E-01	-15.303	3.459	3.76E-01	-2.970	-0.592	5.54E-01	-12.798	6.857	7.59E-01
	L_inferiorparietal	-30.050	-3.420	6.28E-04	-47.272	-12.829	5.17E-03	-11.322	-1.233	2.18E-01	-29.324	6.679	4.98E-01
	L_inferiortemporal	-16.141	-2.639	8.32E-03	-28.128	-4.153	3.56E-02	-2.377	-0.372	7.10E-01	-14.911	10.157	8.49E-01
	L_insula	0.020	0.006	9.95E-01	-6.763	6.803	9.95E-01	-3.900	-1.076	2.82E-01	-11.005	3.204	5.53E-01
	L_isthmuscingulate	4.222	1.853	6.39E-02	-0.243	8.686	1.63E-01	2.741	1.149	2.51E-01	-1.936	7.419	5.37E-01
	L_lateraloccipital	-9.220	-1.059	2.89E-01	-26.280	7.840	4.56E-01	6.907	0.758	4.48E-01	-10.944	24.758	7.05E-01
	L_lateralorbitofrontal	-4.272	-1.256	2.09E-01	-10.937	2.393	3.70E-01	-1.286	-0.361	7.18E-01	-8.267	5.695	8.51E-01
	L_lingual	-5.522	-0.883	3.77E-01	-17.782	6.739	5.32E-01	4.012	0.613	5.40E-01	-8.821	16.846	7.59E-01
	L_medialorbitofrontal	-6.482	-2.143	3.22E-02	-12.411	-0.552	1.03E-01	-2.823	-0.891	3.73E-01	-9.033	3.387	6.44E-01
	L_middletemporal	-12.522	-2.405	1.62E-02	-22.727	-2.316	5.92E-02	-0.930	-0.171	8.64E-01	-11.600	9.740	9.30E-01
	L_paracentral	-1.329	-0.560	5.76E-01	-5.984	3.326	6.92E-01	-2.020	-0.812	4.17E-01	-6.897	2.857	6.71E-01
	L_parahippocampal	1.364	1.182	2.37E-01	-0.898	3.625	4.06E-01	1.947	1.610	1.07E-01	-0.423	4.316	3.55E-01
	L_parsopercularis	-3.499	-0.975	3.30E-01	-10.532	3.534	4.83E-01	-1.828	-0.486	6.27E-01	-9.196	5.540	7.98E-01
	L_parsorbitalis	-1.328	-1.074	2.83E-01	-3.752	1.096	4.48E-01	0.782	0.604	5.46E-01	-1.754	3.319	7.59E-01
	L_parstriangularis	-2.594	-0.907	3.65E-01	-8.199	3.012	5.27E-01	0.055	0.019	9.85E-01	-5.817	5.927	9.90E-01
	L_pericalcarine	-4.770	-1.153	2.49E-01	-12.877	3.337	4.13E-01	2.328	0.538	5.91E-01	-6.157	10.813	7.76E-01
	L_postcentral	-25.494	-4.400	1.09E-05	-36.849	-14.139	2.59E-04	-10.125	-1.672	9.45E-02	-21.990	1.740	3.48E-01
	L_posteriorcingulate	-1.850	-0.721	4.71E-01	-6.876	3.176	6.14E-01	-1.525	-0.567	5.70E-01	-6.792	3.742	7.68E-01
	L_precentral	-9.292	-1.441	1.50E-01	-21.932	3.348	2.94E-01	-5.039	-0.746	4.56E-01	-18.280	8.201	7.12E-01
	L_precuneus	-12.696	-2.018	4.36E-02	-25.027	-0.365	1.20E-01	-8.311	-1.261	2.07E-01	-21.228	4.606	4.82E-01
	L_rostralanteriorcingulate	4.230	1.767	7.72E-02	-0.461	8.922	1.90E-01	1.395	0.557	5.78E-01	-3.518	6.308	7.73E-01
	L_rostralmiddlefrontal	-28.005	-3.062	2.20E-03	-45.929	-10.081	1.27E-02	-6.642	-0.695	4.87E-01	-25.385	12.101	7.34E-01
	L_superiorfrontal	-15.506	-1.531	1.26E-01	-35.360	4.349	2.64E-01	-12.892	-1.214	2.25E-01	-33.699	7.915	5.06E-01
	L_superiorparietal	-30.404	-3.270	1.08E-03	-48.625	-12.183	7.95E-03	-10.485	-1.079	2.81E-01	-29.537	8.567	5.53E-01
	L_superiortemporal	-19.947	-3.537	4.06E-04	-31.000	-8.894	3.63E-03	-15.022	-2.543	1.10E-02	-26.600	-3.444	1.14E-01
	L_supramarginal	-24.140	-2.791	5.26E-03	-41.092	-7.187	2.50E-02	-11.130	-1.230	2.19E-01	-28.871	6.612	4.98E-01
	L_transversetemporal	-2.291	-2.038	4.16E-02	-4.494	-0.088	1.20E-01	-3.020	-2.564	1.04E-02	-5.328	-0.712	1.14E-01

	R_bankssts	-5.419	-2.902	3.71E-03	-9.079	-1.759	1.87E-02	-3.053	-1.562	1.18E-01	-6.884	0.778	3.57E-01
	R_caudalanteriorcingulate	1.398	0.569	5.69E-01	-3.414	6.209	6.88E-01	1.612	0.627	5.31E-01	-3.430	6.654	7.59E-01
	R_caudalmiddlefrontal	-12.844	-2.626	8.65E-03	-22.430	-3.258	3.63E-02	-8.466	-1.652	9.85E-02	-18.509	1.576	3.51E-01
	R_cuneus	-4.074	-1.173	2.41E-01	-10.880	2.733	4.06E-01	2.568	0.707	4.80E-01	-4.553	9.689	7.28E-01
	R_entorhinal	-0.509	-0.400	6.89E-01	-3.000	1.982	7.76E-01	-0.038	-0.029	9.77E-01	-2.647	2.571	9.90E-01
	R_frontalpole	-0.554	-0.982	3.26E-01	-1.660	0.552	4.81E-01	-0.273	-0.462	6.44E-01	-1.431	0.885	8.11E-01
	R_fusiform	-10.872	-2.240	2.51E-02	-20.387	-1.358	8.40E-02	-10.102	-1.986	4.71E-02	-20.073	-0.132	2.72E-01
	R_inferiorparietal	-27.704	-2.723	6.49E-03	-47.648	-7.760	3.02E-02	-6.401	-0.602	5.47E-01	-27.253	14.451	7.59E-01
	R_inferiortemporal	-17.686	-3.104	1.92E-03	-28.855	-6.517	1.15E-02	-9.113	-1.527	1.27E-01	-20.806	2.581	3.71E-01
	R_insula	1.306	0.329	7.42E-01	-6.465	9.077	8.07E-01	-0.361	-0.087	9.31E-01	-8.503	7.781	9.58E-01
	R_isthmuscingulate	2.773	1.355	1.75E-01	-1.238	6.784	3.32E-01	1.244	0.580	5.62E-01	-2.959	5.447	7.66E-01
	R_lateraloccipital	-19.596	-2.130	3.32E-02	-37.627	-1.566	1.04E-01	-8.862	-0.920	3.58E-01	-27.739	10.014	6.38E-01
	R_lateralorbitofrontal	-5.396	-1.309	1.91E-01	-13.477	2.686	3.49E-01	-1.829	-0.424	6.72E-01	-10.293	6.635	8.22E-01
	R_lingual	-1.768	-0.263	7.93E-01	-14.966	11.429	8.44E-01	9.553	1.356	1.75E-01	-4.255	23.362	4.36E-01
	R_medialorbitofrontal	-1.924	-0.717	4.73E-01	-7.182	3.334	6.14E-01	-0.394	-0.140	8.88E-01	-5.903	5.114	9.37E-01
	R_middletemporal	-16.507	-3.238	1.21E-03	-26.499	-6.515	8.34E-03	-8.212	-1.539	1.24E-01	-18.667	2.244	3.68E-01
	R_paracentral	4.427	1.576	1.15E-01	-1.077	9.932	2.47E-01	2.632	0.894	3.71E-01	-3.136	8.400	6.44E-01
	R_parahippocampal	-0.007	-0.007	9.95E-01	-2.202	2.187	9.95E-01	0.949	0.809	4.18E-01	-1.349	3.247	6.71E-01
	R_parsopercularis	-0.734	-0.249	8.03E-01	-6.504	5.037	8.51E-01	1.067	0.346	7.30E-01	-4.980	7.113	8.58E-01
	R_parsorbitalis	-3.144	-2.081	3.75E-02	-6.105	-0.183	1.10E-01	-0.429	-0.271	7.86E-01	-3.526	2.669	8.94E-01
	R_parstriangularis	-2.053	-0.586	5.58E-01	-8.916	4.810	6.82E-01	0.437	0.119	9.05E-01	-6.754	7.627	9.45E-01
	R_pericalcarine	-1.653	-0.376	7.07E-01	-10.270	6.965	7.84E-01	6.888	1.497	1.34E-01	-2.128	15.904	3.77E-01
	R_postcentral	-27.153	-4.573	4.85E-06	-38.790	-15.516	1.48E-04	-15.469	-2.491	1.28E-02	-27.642	-3.295	1.19E-01
	R_posteriorcingulate	-2.137	-0.809	4.18E-01	-7.313	3.039	5.63E-01	-0.655	-0.237	8.13E-01	-6.078	4.768	9.05E-01
	R_precentral	-14.107	-2.154	3.12E-02	-26.941	-1.273	1.01E-01	-9.397	-1.370	1.71E-01	-22.844	4.051	4.34E-01
	R_precuneus	-14.873	-2.317	2.05E-02	-27.454	-2.292	7.20E-02	-9.726	-1.446	1.48E-01	-22.904	3.453	4.01E-01
	R_rostralanteriorcingulate	1.182	0.622	5.34E-01	-2.544	4.908	6.61E-01	1.446	0.726	4.68E-01	-2.459	5.351	7.20E-01
	R_rostralmiddlefrontal	-42.599	-4.293	1.78E-05	-62.049	-23.149	3.17E-04	-17.948	-1.730	8.36E-02	-38.279	2.383	3.27E-01
	R_superiorfrontal	-20.513	-2.019	4.35E-02	-40.424	-0.602	1.20E-01	-18.814	-1.767	7.72E-02	-39.678	2.050	3.18E-01
	R_superiorparietal	-13.910	-1.586	1.13E-01	-31.098	3.277	2.47E-01	3.760	0.410	6.82E-01	-14.219	21.739	8.29E-01
	R_superiortemporal	-17.740	-3.637	2.77E-04	-27.300	-8.180	2.69E-03	-16.978	-3.322	8.97E-04	-26.996	-6.960	3.84E-02
	R_supramarginal	-19.223	-2.677	7.44E-03	-33.299	-5.148	3.31E-02	-12.995	-1.727	8.42E-02	-27.741	1.751	3.27E-01
	R_transversetemporal	-2.713	-3.698	2.18E-04	-4.151	-1.275	2.29E-03	-3.066	-3.988	6.69E-05	-4.572	-1.559	4.77E-03
**Cortical Mean Thickness**	L_bankssts	0.003	1.051	2.93E-01	-0.003	0.010	4.58E-01	0.005	1.474	1.41E-01	-0.002	0.011	3.86E-01
	L_caudalanteriorcingulate	0.003	0.351	7.26E-01	-0.013	0.018	7.97E-01	0.007	0.810	4.18E-01	-0.009	0.023	6.71E-01
	L_caudalmiddlefrontal	0.005	1.690	9.11E-02	-0.001	0.010	2.12E-01	0.004	1.517	1.29E-01	-0.001	0.010	3.73E-01
	L_cuneus	0.011	4.105	4.06E-05	0.006	0.016	6.21E-04	0.004	1.400	1.62E-01	-0.002	0.009	4.27E-01
	L_entorhinal	0.002	0.405	6.85E-01	-0.009	0.013	7.76E-01	0.014	2.473	1.34E-02	0.003	0.025	1.19E-01
	L_frontalpole	0.009	2.121	3.40E-02	0.001	0.018	1.05E-01	0.001	0.314	7.53E-01	-0.007	0.010	8.76E-01
	L_fusiform	-0.003	-1.351	1.77E-01	-0.008	0.001	3.32E-01	0.001	0.218	8.27E-01	-0.004	0.005	9.05E-01
	L_inferiorparietal	0.005	2.026	4.28E-02	0.000	0.009	1.20E-01	0.003	1.434	1.52E-01	-0.001	0.008	4.06E-01
	L_inferiortemporal	-0.003	-1.017	3.09E-01	-0.008	0.002	4.70E-01	-0.001	-0.443	6.58E-01	-0.006	0.004	8.17E-01
	L_insula	-0.003	-0.991	3.22E-01	-0.009	0.003	4.81E-01	0.004	1.071	2.84E-01	-0.003	0.010	5.53E-01
	L_isthmuscingulate	-0.001	-0.473	6.36E-01	-0.008	0.005	7.52E-01	-0.003	-0.854	3.93E-01	-0.009	0.004	6.68E-01
	L_lateraloccipital	0.012	5.350	8.94E-08	0.008	0.016	7.92E-06	0.005	2.312	2.08E-02	0.001	0.010	1.48E-01
	L_lateralorbitofrontal	0.002	0.853	3.94E-01	-0.003	0.007	5.44E-01	0.004	1.604	1.09E-01	-0.001	0.009	3.55E-01

	L_lingual	0.008	3.251	1.15E-03	0.003	0.014	8.22E-03	0.004	1.311	1.90E-01	-0.002	0.009	4.59E-01
	L_medialorbitofrontal	-0.002	-0.879	3.80E-01	-0.008	0.003	5.32E-01	0.000	-0.151	8.80E-01	-0.006	0.005	9.37E-01
	L_middletemporal	-0.001	-0.204	8.38E-01	-0.006	0.005	8.75E-01	0.002	0.646	5.18E-01	-0.004	0.008	7.49E-01
	L_paracentral	0.002	0.446	6.55E-01	-0.005	0.008	7.62E-01	0.006	1.611	1.07E-01	-0.001	0.013	3.55E-01
	L_parahippocampal	-0.009	-1.611	1.07E-01	-0.019	0.002	2.43E-01	-0.001	-0.215	8.30E-01	-0.012	0.010	9.05E-01
	L_parsopercularis	0.003	1.274	2.03E-01	-0.002	0.009	3.63E-01	0.005	1.630	1.03E-01	-0.001	0.010	3.55E-01
	L_parsorbitalis	0.007	2.266	2.35E-02	0.001	0.014	7.98E-02	0.006	1.748	8.06E-02	-0.001	0.013	3.25E-01
	L_parstriangularis	0.006	2.197	2.80E-02	0.001	0.011	9.23E-02	0.004	1.585	1.13E-01	-0.001	0.010	3.55E-01
	L_pericalcarine	0.009	3.339	8.42E-04	0.004	0.014	6.44E-03	0.002	0.673	5.01E-01	-0.003	0.007	7.34E-01
	L_postcentral	0.008	2.920	3.51E-03	0.002	0.013	1.83E-02	0.003	1.167	2.43E-01	-0.002	0.009	5.35E-01
	L_posteriorcingulate	-0.001	-0.419	6.75E-01	-0.008	0.005	7.76E-01	-0.001	-0.210	8.33E-01	-0.008	0.006	9.05E-01
	L_precentral	0.003	1.020	3.08E-01	-0.003	0.010	4.70E-01	0.005	1.621	1.05E-01	-0.001	0.012	3.55E-01
	L_precuneus	0.008	3.364	7.71E-04	0.004	0.013	6.11E-03	0.007	2.602	9.29E-03	0.002	0.012	1.14E-01
	L_rostralanteriorcingulate	-0.001	-0.286	7.75E-01	-0.009	0.007	8.31E-01	0.003	0.835	4.04E-01	-0.005	0.011	6.70E-01
	L_rostralmiddlefrontal	0.010	4.150	3.34E-05	0.005	0.014	5.50E-04	0.006	2.536	1.12E-02	0.001	0.011	1.14E-01
	L_superiorfrontal	0.006	2.099	3.58E-02	0.000	0.011	1.08E-01	0.007	2.515	1.19E-02	0.002	0.012	1.16E-01
	L_superiorparietal	0.010	3.952	7.78E-05	0.005	0.014	9.79E-04	0.005	1.822	6.84E-02	0.000	0.010	2.92E-01
	L_superiortemporal	-0.004	-1.423	1.55E-01	-0.010	0.002	3.01E-01	0.000	0.140	8.89E-01	-0.006	0.007	9.37E-01
	L_supramarginal	0.005	1.993	4.63E-02	0.000	0.010	1.25E-01	0.006	2.313	2.07E-02	0.001	0.011	1.48E-01
	L_transversetemporal	-0.007	-1.591	1.12E-01	-0.016	0.002	2.47E-01	-0.010	-2.186	2.88E-02	-0.019	-0.001	1.99E-01
	R_bankssts	0.004	1.240	2.15E-01	-0.002	0.011	3.76E-01	0.005	1.564	1.18E-01	-0.001	0.012	3.57E-01
	R_caudalanteriorcingulate	0.010	1.624	1.04E-01	-0.002	0.022	2.40E-01	0.010	1.512	1.31E-01	-0.003	0.023	3.73E-01
	R_caudalmiddlefrontal	0.007	2.689	7.17E-03	0.002	0.013	3.26E-02	0.007	2.550	1.08E-02	0.002	0.013	1.14E-01
	R_cuneus	0.013	5.311	1.11E-07	0.008	0.018	7.92E-06	0.008	2.865	4.17E-03	0.002	0.013	8.12E-02
	R_entorhinal	0.007	1.142	2.53E-01	-0.005	0.018	4.17E-01	0.013	2.159	3.09E-02	0.001	0.026	2.07E-01
	R_frontalpole	0.019	4.377	1.21E-05	0.010	0.027	2.59E-04	0.010	2.336	1.95E-02	0.002	0.019	1.48E-01
	R_fusiform	0.004	1.470	1.42E-01	-0.001	0.009	2.83E-01	0.007	2.674	7.51E-03	0.002	0.012	1.14E-01
	R_inferiorparietal	0.007	2.990	2.79E-03	0.002	0.012	1.50E-02	0.005	1.944	5.19E-02	0.000	0.010	2.82E-01
	R_inferiortemporal	0.003	1.368	1.71E-01	-0.001	0.008	3.27E-01	0.004	1.592	1.11E-01	-0.001	0.009	3.55E-01
	R_insula	0.001	0.284	7.77E-01	-0.005	0.007	8.31E-01	0.005	1.574	1.15E-01	-0.001	0.012	3.57E-01
	R_isthmuscingulate	0.003	1.015	3.10E-01	-0.003	0.010	4.70E-01	0.004	1.098	2.72E-01	-0.003	0.011	5.47E-01
	R_lateraloccipital	0.014	5.836	5.47E-09	0.009	0.018	1.17E-06	0.007	2.656	7.92E-03	0.002	0.011	1.14E-01
	R_lateralorbitofrontal	0.003	0.994	3.20E-01	-0.003	0.008	4.81E-01	0.006	1.991	4.65E-02	0.000	0.011	2.72E-01
	R_lingual	0.013	5.100	3.45E-07	0.008	0.018	1.48E-05	0.008	2.989	2.81E-03	0.003	0.013	6.67E-02
	R_medialorbitofrontal	0.002	0.741	4.59E-01	-0.003	0.008	6.06E-01	0.004	1.493	1.36E-01	-0.001	0.010	3.77E-01
	R_middletemporal	0.009	3.425	6.16E-04	0.004	0.015	5.17E-03	0.010	3.614	3.02E-04	0.005	0.016	1.62E-02
	R_paracentral	0.003	0.844	3.99E-01	-0.004	0.010	5.47E-01	0.007	1.881	5.99E-02	0.000	0.014	2.85E-01
	R_parahippocampal	0.004	0.834	4.04E-01	-0.005	0.013	5.51E-01	0.008	1.681	9.28E-02	-0.001	0.017	3.48E-01
	R_parsopercularis	0.005	1.844	6.51E-02	0.000	0.010	1.64E-01	0.005	1.899	5.76E-02	0.000	0.011	2.84E-01
	R_parsorbitalis	0.010	2.989	2.80E-03	0.003	0.016	1.50E-02	0.009	2.625	8.67E-03	0.002	0.015	1.14E-01
	R_parstriangularis	0.008	3.182	1.46E-03	0.003	0.013	9.50E-03	0.007	2.430	1.51E-02	0.001	0.012	1.30E-01
	R_pericalcarine	0.011	4.294	1.77E-05	0.006	0.016	3.17E-04	0.004	1.386	1.66E-01	-0.002	0.009	4.33E-01
	R_postcentral	0.010	3.691	2.24E-04	0.005	0.016	2.29E-03	0.005	1.833	6.68E-02	0.000	0.011	2.92E-01
	R_posteriorcingulate	0.002	0.647	5.18E-01	-0.004	0.009	6.48E-01	0.003	0.955	3.40E-01	-0.004	0.010	6.22E-01
	R_precentral	0.008	2.550	1.08E-02	0.002	0.015	4.25E-02	0.009	2.783	5.40E-03	0.003	0.016	9.62E-02
	R_precuneus	0.009	3.536	4.07E-04	0.004	0.014	3.63E-03	0.007	2.868	4.13E-03	0.002	0.012	8.12E-02

	R_rostralanteriorcingulate	0.003	0.752	4.52E-01	-0.005	0.011	6.01E-01	0.005	1.098	2.72E-01	-0.004	0.013	5.47E-01
	R_rostralmiddlefrontal	0.012	5.257	1.49E-07	0.007	0.016	7.97E-06	0.007	3.103	1.92E-03	0.003	0.012	5.13E-02
	R_superiorfrontal	0.008	3.222	1.28E-03	0.003	0.013	8.55E-03	0.008	3.105	1.91E-03	0.003	0.014	5.13E-02
	R_superiorparietal	0.012	5.023	5.16E-07	0.008	0.017	1.84E-05	0.006	2.387	1.70E-02	0.001	0.011	1.40E-01
	R_superiortemporal	0.000	0.163	8.71E-01	-0.005	0.006	9.00E-01	0.005	1.598	1.10E-01	-0.001	0.011	3.55E-01
	R_supramarginal	0.007	2.521	1.17E-02	0.001	0.012	4.39E-02	0.006	2.352	1.87E-02	0.001	0.012	1.48E-01
	R_transversetemporal	-0.008	-1.700	8.91E-02	-0.017	0.001	2.09E-01	-0.009	-1.938	5.26E-02	-0.019	0.000	2.82E-01
**Cortical Volume**	L_bankssts	-20.572	-2.863	4.20E-03	-34.653	-6.490	2.04E-02	-7.599	-1.011	3.12E-01	-22.328	7.130	5.80E-01
	L_caudalanteriorcingulate	-6.625	-0.873	3.83E-01	-21.497	8.248	5.32E-01	-8.755	-1.101	2.71E-01	-24.340	6.830	5.47E-01
	L_caudalmiddlefrontal	-11.175	-0.812	4.17E-01	-38.138	15.788	5.63E-01	1.439	0.100	9.20E-01	-26.791	29.670	9.52E-01
	L_cuneus	3.562	0.434	6.64E-01	-12.513	19.638	7.68E-01	7.256	0.844	3.98E-01	-9.586	24.097	6.70E-01
	L_entorhinal	-3.198	-0.629	5.29E-01	-13.159	6.764	6.59E-01	2.827	0.531	5.95E-01	-7.606	13.260	7.77E-01
	L_frontalpole	0.055	0.035	9.72E-01	-3.006	3.116	9.86E-01	-0.731	-0.447	6.55E-01	-3.934	2.473	8.17E-01
	L_fusiform	-27.327	-1.859	6.31E-02	-56.146	1.491	1.63E-01	-6.590	-0.428	6.68E-01	-36.754	23.573	8.22E-01
	L_inferiorparietal	-61.423	-2.542	1.10E-02	-108.773	-14.073	4.25E-02	-17.236	-0.682	4.95E-01	-66.739	32.266	7.34E-01
	L_inferiortemporal	-58.753	-3.147	1.65E-03	-95.343	-22.163	1.04E-02	-13.089	-0.671	5.02E-01	-51.324	25.145	7.34E-01
	L_insula	-7.722	-0.736	4.62E-01	-28.285	12.842	6.06E-01	-3.076	-0.280	7.79E-01	-24.604	18.452	8.94E-01
	L_isthmuscingulate	8.080	1.470	1.41E-01	-2.690	18.851	2.83E-01	3.123	0.543	5.87E-01	-8.159	14.404	7.76E-01
	L_lateraloccipital	37.563	1.732	8.34E-02	-4.955	80.081	2.00E-01	41.623	1.832	6.70E-02	-2.919	86.165	2.92E-01
	L_lateralorbitofrontal	-8.204	-0.803	4.22E-01	-28.233	11.824	5.64E-01	6.142	0.575	5.66E-01	-14.805	27.089	7.66E-01
	L_lingual	13.796	0.884	3.77E-01	-16.808	44.400	5.32E-01	18.539	1.133	2.57E-01	-13.524	50.603	5.41E-01
	L_medialorbitofrontal	-23.309	-2.671	7.58E-03	-40.415	-6.204	3.31E-02	-9.340	-1.023	3.06E-01	-27.233	8.552	5.75E-01
	L_middletemporal	-38.870	-2.301	2.14E-02	-71.975	-5.764	7.38E-02	3.361	0.191	8.49E-01	-31.201	37.924	9.17E-01
	L_paracentral	-2.712	-0.404	6.86E-01	-15.873	10.448	7.76E-01	1.892	0.269	7.88E-01	-11.888	15.671	8.94E-01
	L_parahippocampal	-1.964	-0.456	6.48E-01	-10.398	6.470	7.58E-01	4.654	1.033	3.01E-01	-4.174	13.482	5.71E-01
	L_parsopercularis	-5.619	-0.537	5.91E-01	-26.113	14.875	7.07E-01	0.793	0.072	9.42E-01	-20.665	22.252	9.61E-01
	L_parsorbitalis	0.464	0.120	9.05E-01	-7.125	8.052	9.31E-01	5.586	1.379	1.68E-01	-2.355	13.526	4.33E-01
	L_parstriangularis	-0.723	-0.092	9.27E-01	-16.164	14.718	9.49E-01	4.434	0.538	5.91E-01	-11.733	20.601	7.76E-01
	L_pericalcarine	3.196	0.391	6.95E-01	-12.805	19.198	7.76E-01	6.216	0.727	4.67E-01	-10.548	22.981	7.20E-01
	L_postcentral	-30.944	-1.891	5.86E-02	-63.016	1.129	1.55E-01	-13.883	-0.810	4.18E-01	-47.458	19.692	6.71E-01
	L_posteriorcingulate	-7.768	-1.127	2.60E-01	-21.279	5.743	4.21E-01	-6.043	-0.837	4.03E-01	-20.201	8.115	6.70E-01
	L_precentral	-13.076	-0.656	5.12E-01	-52.159	26.006	6.44E-01	9.673	0.464	6.43E-01	-31.193	50.539	8.11E-01
	L_precuneus	-5.355	-0.323	7.47E-01	-37.833	27.123	8.07E-01	0.323	0.019	9.85E-01	-33.688	34.333	9.90E-01
	L_rostralanteriorcingulate	10.582	1.575	1.15E-01	-2.586	23.750	2.47E-01	6.535	0.928	3.53E-01	-7.263	20.333	6.35E-01
	L_rostralmiddlefrontal	-26.982	-1.100	2.71E-01	-75.042	21.079	4.33E-01	10.318	0.402	6.88E-01	-39.956	60.592	8.31E-01
	L_superiorfrontal	-12.319	-0.402	6.88E-01	-72.396	47.759	7.76E-01	7.087	0.221	8.25E-01	-55.765	69.938	9.05E-01
	L_superiorparietal	-27.497	-1.163	2.45E-01	-73.847	18.853	4.10E-01	-5.501	-0.222	8.24E-01	-53.992	42.990	9.05E-01
	L_superiortemporal	-76.436	-3.953	7.76E-05	-114.334	-38.539	9.79E-04	-42.915	-2.123	3.37E-02	-82.527	-3.303	2.12E-01
	L_supramarginal	-50.036	-2.112	3.47E-02	-96.473	-3.599	1.06E-01	-8.884	-0.359	7.20E-01	-57.418	39.650	8.51E-01
	L_transversetemporal	-9.058	-3.100	1.94E-03	-14.785	-3.331	1.15E-02	-12.313	-4.025	5.73E-05	-18.309	-6.317	4.77E-03
	R_bankssts	-11.446	-1.871	6.13E-02	-23.433	0.541	1.60E-01	-3.822	-0.597	5.50E-01	-16.369	8.724	7.59E-01
	R_caudalanteriorcingulate	8.864	1.175	2.40E-01	-5.921	23.648	4.06E-01	9.235	1.168	2.43E-01	-6.259	24.728	5.35E-01
	R_caudalmiddlefrontal	-21.123	-1.517	1.29E-01	-48.414	6.169	2.69E-01	-8.802	-0.604	5.46E-01	-37.384	19.781	7.59E-01
	R_cuneus	12.901	1.608	1.08E-01	-2.820	28.623	2.43E-01	16.021	1.906	5.66E-02	-0.450	32.492	2.84E-01
	R_entorhinal	0.923	0.213	8.32E-01	-7.586	9.433	8.75E-01	5.285	1.162	2.45E-01	-3.627	14.197	5.35E-01
	R_frontalpole	3.946	2.094	3.63E-02	0.252	7.641	1.08E-01	2.234	1.132	2.58E-01	-1.633	6.101	5.41E-01

	R_fusiform	-18.847	-1.301	1.93E-01	-47.237	9.543	3.50E-01	-5.715	-0.377	7.06E-01	-35.438	24.007	8.49E-01
	R_inferiorparietal	-42.529	-1.554	1.20E-01	-96.167	11.108	2.55E-01	3.424	0.120	9.05E-01	-52.672	59.520	9.45E-01
	R_inferiortemporal	-44.471	-2.543	1.10E-02	-78.751	-10.192	4.25E-02	-16.461	-0.900	3.68E-01	-52.329	19.406	6.44E-01
	R_insula	5.114	0.456	6.49E-01	-16.881	27.110	7.58E-01	10.081	0.858	3.91E-01	-12.951	33.113	6.68E-01
	R_isthmuscingulate	8.909	1.716	8.62E-02	-1.267	19.086	2.05E-01	5.638	1.036	3.00E-01	-5.023	16.298	5.71E-01
	R_lateraloccipital	23.753	1.035	3.01E-01	-21.220	68.727	4.66E-01	12.619	0.525	6.00E-01	-34.487	59.725	7.77E-01
	R_lateralorbitofrontal	-9.878	-0.920	3.58E-01	-30.920	11.164	5.20E-01	8.096	0.721	4.71E-01	-13.904	30.096	7.20E-01
	R_lingual	38.755	2.330	1.98E-02	6.155	71.355	7.07E-02	44.841	2.574	1.01E-02	10.697	78.984	1.14E-01
	R_medialorbitofrontal	-2.518	-0.328	7.43E-01	-17.582	12.546	8.07E-01	6.480	0.806	4.20E-01	-9.284	22.244	6.71E-01
	R_middletemporal	-18.923	-1.122	2.62E-01	-51.992	14.147	4.22E-01	9.176	0.520	6.03E-01	-25.395	43.746	7.77E-01
	R_paracentral	15.333	1.950	5.12E-02	-0.082	30.748	1.37E-01	16.805	2.040	4.14E-02	0.660	32.949	2.53E-01
	R_parahippocampal	2.143	0.586	5.58E-01	-5.029	9.315	6.82E-01	7.271	1.898	5.77E-02	-0.237	14.780	2.84E-01
	R_parsopercularis	4.002	0.476	6.34E-01	-12.460	20.464	7.52E-01	9.693	1.102	2.70E-01	-7.547	26.933	5.47E-01
	R_parsorbitalis	-1.724	-0.370	7.12E-01	-10.863	7.415	7.85E-01	5.337	1.095	2.74E-01	-4.219	14.893	5.47E-01
	R_parstriangularis	5.467	0.573	5.67E-01	-13.237	24.171	6.88E-01	9.729	0.973	3.30E-01	-9.859	29.318	6.09E-01
	R_pericalcarine	13.007	1.504	1.33E-01	-3.948	29.962	2.73E-01	16.441	1.814	6.97E-02	-1.323	34.205	2.92E-01
	R_postcentral	-20.918	-1.271	2.04E-01	-53.173	11.336	3.63E-01	-13.138	-0.762	4.46E-01	-46.918	20.641	7.05E-01
	R_posteriorcingulate	-4.739	-0.685	4.93E-01	-18.290	8.812	6.28E-01	0.516	0.071	9.43E-01	-13.680	14.712	9.61E-01
	R_precentral	0.588	0.029	9.77E-01	-39.460	40.636	9.86E-01	19.169	0.897	3.70E-01	-22.738	61.076	6.44E-01
	R_precuneus	-6.530	-0.391	6.96E-01	-39.289	26.229	7.76E-01	1.865	0.107	9.15E-01	-32.445	36.175	9.51E-01
	R_rostralanteriorcingulate	4.986	0.876	3.81E-01	-6.168	16.140	5.32E-01	6.845	1.148	2.51E-01	-4.844	18.534	5.37E-01
	R_rostralmiddlefrontal	-46.505	-1.751	7.99E-02	-98.546	5.536	1.94E-01	-8.186	-0.295	7.68E-01	-62.623	46.251	8.89E-01
	R_superiorfrontal	-6.285	-0.207	8.36E-01	-65.756	53.186	8.75E-01	0.290	0.009	9.93E-01	-61.907	62.487	9.93E-01
	R_superiorparietal	29.282	1.312	1.90E-01	-14.466	73.031	3.49E-01	38.662	1.655	9.80E-02	-7.129	84.453	3.51E-01
	R_superiortemporal	-51.638	-3.016	2.57E-03	-85.199	-18.078	1.45E-02	-32.915	-1.838	6.62E-02	-68.023	2.194	2.92E-01
	R_supramarginal	-29.845	-1.462	1.44E-01	-69.866	10.176	2.85E-01	-12.717	-0.595	5.52E-01	-54.610	29.176	7.59E-01
	R_transversetemporal	-9.289	-4.522	6.17E-06	-13.315	-5.263	1.65E-04	-10.743	-4.996	5.92E-07	-14.958	-6.529	1.27E-04
	Laccumb	1.153	0.886	3.75E-01	-1.396	3.701	5.32E-01	0.598	0.440	6.60E-01	-2.068	3.264	8.17E-01
	Lamyg	12.589	3.995	6.50E-05	6.413	18.765	9.27E-04	10.561	3.202	1.37E-03	4.097	17.026	4.88E-02
	Lcaud	13.509	2.026	4.28E-02	0.437	26.580	1.20E-01	9.132	1.308	1.91E-01	-4.548	22.811	4.59E-01
	Lhippo	7.619	1.342	1.79E-01	-3.504	18.741	3.34E-01	5.604	0.944	3.45E-01	-6.037	17.245	6.26E-01
	LLatVent	142.555	1.193	2.33E-01	-91.741	376.852	4.02E-01	42.465	0.340	7.34E-01	-202.622	287.551	8.59E-01
	Lpal	-0.180	-0.056	9.55E-01	-6.435	6.076	9.73E-01	6.517	1.954	5.08E-02	-0.021	13.055	2.82E-01
	Lput	29.266	3.737	1.87E-04	13.918	44.615	2.11E-03	17.575	2.146	3.19E-02	1.527	33.622	2.07E-01
**Subcortical Volume**	Lthal	-1.649	-0.185	8.53E-01	-19.105	15.807	8.86E-01	2.481	0.266	7.90E-01	-15.780	20.743	8.94E-01
	Raccumb	1.741	1.409	1.59E-01	-0.681	4.163	3.07E-01	1.739	1.345	1.79E-01	-0.794	4.272	4.39E-01
	Ramyg	9.056	2.899	3.75E-03	2.933	15.178	1.87E-02	5.536	1.693	9.05E-02	-0.873	11.945	3.46E-01
	Rcaud	17.304	2.539	1.11E-02	3.945	30.663	4.25E-02	9.151	1.283	1.99E-01	-4.826	23.127	4.69E-01
	Rhippo	4.244	0.707	4.79E-01	-7.517	16.005	6.14E-01	1.607	0.256	7.98E-01	-10.707	13.921	8.99E-01
	RLatVent	102.906	0.982	3.26E-01	-102.428	308.241	4.81E-01	16.288	0.149	8.82E-01	-198.502	231.079	9.37E-01
	Rpal	-2.128	-0.677	4.98E-01	-8.291	4.034	6.31E-01	3.421	1.040	2.98E-01	-3.027	9.868	5.71E-01
	Rput	20.345	2.575	1.00E-02	4.862	35.829	4.13E-02	10.694	1.294	1.96E-01	-5.502	26.890	4.65E-01
	Rthal	19.819	2.402	1.63E-02	3.647	35.991	5.92E-02	11.782	1.365	1.72E-01	-5.134	28.698	4.34E-01

Supplementary Table 4: The association between tinnitus and brain surface area, mean thickness and volume in the UK Biobank. Values highlighted in red are the significant values at respective P-value and corrected P-value at 0.005. ICV: intracranial volume; CVD: cardiovascular disease; BMI: body mass index; TDI, Townsend deprivation index; ROI: region of interest; IDP: imaging derived phenotypes; CI: confidence interval; FDR: false discovery rate.

Cases (3,141) vs. Controls (3,141): Tinnitus vs. Control	IDP	Model 1: IDP ~ Tinnitus + Age + Sex + ICV						Model 2: IDP ~ Tinnitus + Age + Sex + ICV + Smoking + Alcohol + Diabetes + CVD + BMI + TDI					
		Estimate	T-value	P-value	Lower-CI	Upper-CI	FDR	Estimate	T-value	P-value	Lower-CI	Upper-CI	FDR
Cortical Surface Area	L_bankssts	-8.428	-2.384	1.71E-02	-15.355	-1.500	4.32E-02	-0.915	-0.215	8.30E-01	-9.243	7.414	9.42E-01
	L_caudalanteriorcingulate	1.569	0.504	6.14E-01	-4.536	7.674	7.20E-01	-2.208	-0.589	5.56E-01	-9.555	5.138	7.63E-01
	L_caudalmiddlefrontal	-13.102	-1.872	6.12E-02	-26.819	0.614	1.20E-01	-5.023	-0.596	5.51E-01	-21.531	11.486	7.63E-01
	L_cuneus	-16.555	-3.197	1.40E-03	-26.704	-6.406	5.15E-03	-9.321	-1.497	1.35E-01	-21.527	2.885	3.13E-01
	L_entorhinal	0.449	0.203	8.39E-01	-3.892	4.790	9.07E-01	1.757	0.659	5.10E-01	-3.467	6.981	7.32E-01
	L_frontalpole	-2.881	-4.423	9.90E-06	-4.157	-1.604	8.83E-05	-2.142	-2.733	6.30E-03	-3.678	-0.606	6.96E-02
	L_fusiform	-9.092	-1.298	1.94E-01	-22.825	4.642	2.93E-01	-6.173	-0.732	4.64E-01	-22.697	10.351	6.90E-01
	L_inferiorparietal	-49.685	-3.866	1.12E-04	-74.875	-24.494	6.68E-04	-14.503	-0.940	3.47E-01	-44.747	15.741	5.81E-01
	L_inferiortemporal	-31.998	-3.599	3.21E-04	-49.421	-14.574	1.51E-03	-0.295	-0.028	9.78E-01	-21.198	20.608	9.86E-01
	L_insula	-1.399	-0.279	7.80E-01	-11.237	8.438	8.63E-01	-9.600	-1.589	1.12E-01	-21.437	2.238	2.85E-01
	L_isthmuscingulate	4.647	1.420	1.56E-01	-1.768	11.062	2.48E-01	5.036	1.278	2.01E-01	-2.687	12.759	3.99E-01
	L_lateraloccipital	-39.367	-3.064	2.19E-03	-64.548	-14.187	7.69E-03	-6.007	-0.389	6.97E-01	-36.273	24.258	8.69E-01
	L_lateralorbitofrontal	-11.597	-2.363	1.81E-02	-21.216	-1.979	4.46E-02	-7.330	-1.241	2.15E-01	-18.905	4.244	4.17E-01
	L_lingual	-11.827	-1.309	1.90E-01	-29.529	5.875	2.89E-01	-0.576	-0.053	9.58E-01	-21.865	20.714	9.86E-01
	L_medialorbitofrontal	-11.817	-2.676	7.47E-03	-20.473	-3.162	2.28E-02	-4.070	-0.766	4.44E-01	-14.484	6.344	6.75E-01
	L_middletemporal	-15.734	-2.077	3.79E-02	-30.582	-0.885	8.10E-02	4.834	0.531	5.95E-01	-13.002	22.670	7.84E-01
	L_paracentral	-2.660	-0.764	4.45E-01	-9.487	4.166	5.81E-01	-3.885	-0.927	3.54E-01	-12.099	4.329	5.87E-01
	L_parahippocampal	-0.128	-0.076	9.39E-01	-3.419	3.163	9.75E-01	0.993	0.492	6.23E-01	-2.968	4.955	7.98E-01
	L_parsopercularis	-17.160	-3.237	1.22E-03	-27.551	-6.768	4.57E-03	-18.027	-2.825	4.75E-03	-30.535	-5.518	6.96E-02
	L_parsorbitalis	-4.077	-2.284	2.24E-02	-7.576	-0.578	5.21E-02	0.984	0.458	6.47E-01	-3.223	5.190	8.19E-01
	L_parstriangularis	-14.374	-3.477	5.10E-04	-22.475	-6.272	2.23E-03	-11.754	-2.363	1.82E-02	-21.502	-2.005	1.00E-01
	L_pericalcarine	-12.225	-2.022	4.32E-02	-24.075	-0.376	8.98E-02	-5.847	-0.804	4.22E-01	-20.105	8.411	6.58E-01
	L_postcentral	-40.242	-4.777	1.82E-06	-56.753	-23.732	2.16E-05	-13.273	-1.312	1.90E-01	-33.105	6.559	3.87E-01
	L_posteriorcingulate	0.946	0.261	7.94E-01	-6.170	8.063	8.63E-01	2.760	0.632	5.28E-01	-5.805	11.326	7.43E-01
	L_precentral	-7.213	-0.766	4.44E-01	-25.679	11.253	5.81E-01	0.330	0.029	9.77E-01	-21.885	22.545	9.86E-01
	L_precuneus	-26.046	-2.822	4.79E-03	-44.136	-7.956	1.55E-02	-23.502	-2.116	3.44E-02	-45.274	-1.730	1.47E-01
	L_rostralanteriorcingulate	3.987	1.153	2.49E-01	-2.791	10.765	3.63E-01	-3.612	-0.869	3.85E-01	-11.760	4.537	6.20E-01
	L_rostralmiddlefrontal	-67.288	-5.067	4.16E-07	-93.317	-41.260	8.74E-06	-32.351	-2.026	4.28E-02	-63.641	-1.061	1.61E-01
	L_superiorfrontal	-36.529	-2.507	1.22E-02	-65.081	-7.976	3.38E-02	-32.164	-1.835	6.65E-02	-66.512	2.185	2.09E-01
	L_superiorparietal	-68.614	-5.052	4.49E-07	-95.232	-41.997	8.74E-06	-39.887	-2.443	1.46E-02	-71.891	-7.882	9.37E-02
	L_superiortemporal	-31.058	-3.827	1.31E-04	-46.965	-15.152	7.25E-04	-32.020	-3.278	1.05E-03	-51.163	-12.877	3.21E-02
	L_supramarginal	-40.836	-3.239	1.21E-03	-65.550	-16.122	4.57E-03	-20.465	-1.349	1.77E-01	-50.198	9.267	3.72E-01
	L_transversetemporal	-3.068	-1.888	5.90E-02	-6.253	0.117	1.17E-01	-4.775	-2.442	1.46E-02	-8.607	-0.942	9.37E-02
	R_bankssts	-6.417	-2.342	1.92E-02	-11.787	-1.047	4.67E-02	0.842	0.256	7.98E-01	-5.602	7.287	9.28E-01

	R_caudalanteriorcingulate	0.138	0.039	9.69E-01	-6.762	7.038	9.86E-01	-0.086	-0.020	9.84E-01	-8.392	8.221	9.86E-01
	R_caudalmiddlefrontal	-24.769	-3.464	5.37E-04	-38.785	-10.752	2.30E-03	-16.961	-1.971	4.88E-02	-33.831	-0.091	1.71E-01
	R_cuneus	-19.561	-3.865	1.12E-04	-29.480	-9.641	6.68E-04	-12.940	-2.125	3.36E-02	-24.876	-1.005	1.47E-01
	R_entorhinal	-2.822	-1.529	1.26E-01	-6.439	0.796	2.10E-01	-1.940	-0.873	3.83E-01	-6.295	2.414	6.20E-01
	R_frontalpole	0.583	0.717	4.73E-01	-1.009	2.175	6.03E-01	0.394	0.403	6.87E-01	-1.522	2.309	8.65E-01
	R_fusiform	-21.814	-3.068	2.17E-03	-35.750	-7.877	7.69E-03	-20.381	-2.382	1.72E-02	-37.152	-3.611	9.98E-02
	R_inferiorparietal	-24.903	-1.685	9.20E-02	-53.869	4.063	1.60E-01	9.285	0.523	6.01E-01	-25.520	44.089	7.84E-01
	R_inferiortemporal	-23.948	-2.887	3.90E-03	-40.206	-7.690	1.30E-02	-6.906	-0.693	4.89E-01	-26.448	12.637	7.11E-01
	R_insula	2.979	0.514	6.07E-01	-8.381	14.340	7.18E-01	0.241	0.035	9.72E-01	-13.424	13.907	9.86E-01
	R_isthmuscingulate	2.031	0.689	4.91E-01	-3.747	7.810	6.18E-01	-1.695	-0.478	6.33E-01	-8.649	5.259	8.06E-01
	R_lateraloccipital	-29.532	-2.207	2.74E-02	-55.762	-3.303	6.17E-02	-20.653	-1.283	2.00E-01	-52.209	10.904	3.99E-01
	R_lateralorbitofrontal	-16.757	-2.779	5.48E-03	-28.577	-4.937	1.75E-02	-7.975	-1.099	2.72E-01	-22.192	6.242	5.01E-01
	R_lingual	-17.758	-1.804	7.13E-02	-37.054	1.538	1.38E-01	-10.385	-0.877	3.81E-01	-33.599	12.828	6.20E-01
	R_medialorbitofrontal	-10.247	-2.671	7.59E-03	-17.767	-2.727	2.29E-02	-7.070	-1.531	1.26E-01	-16.121	1.980	3.06E-01
	R_middletemporal	-13.482	-1.818	6.91E-02	-28.014	1.050	1.34E-01	5.127	0.576	5.65E-01	-12.326	22.580	7.65E-01
	R_paracentral	5.370	1.319	1.87E-01	-2.611	13.351	2.86E-01	3.743	0.764	4.45E-01	-5.862	13.348	6.75E-01
	R_parahippocampal	-0.438	-0.268	7.89E-01	-3.643	2.767	8.63E-01	1.884	0.957	3.38E-01	-1.972	5.739	5.75E-01
	R_parsopercularis	-11.276	-2.629	8.58E-03	-19.682	-2.871	2.51E-02	-10.494	-2.033	4.21E-02	-20.610	-0.377	1.61E-01
	R_parsorbitalis	-7.345	-3.382	7.24E-04	-11.602	-3.088	2.99E-03	-1.321	-0.506	6.13E-01	-6.439	3.796	7.90E-01
	R_parstriangularis	-9.857	-1.920	5.48E-02	-19.917	0.203	1.12E-01	-9.287	-1.503	1.33E-01	-21.395	2.821	3.12E-01
	R_pericalcarine	-13.691	-2.118	3.42E-02	-26.360	-1.023	7.41E-02	-8.189	-1.053	2.93E-01	-23.436	7.058	5.15E-01
	R_postcentral	-43.047	-4.984	6.38E-07	-59.974	-26.120	1.14E-05	-25.381	-2.445	1.45E-02	-45.731	-5.031	9.37E-02
	R_posteriorcingulate	-2.523	-0.680	4.97E-01	-9.796	4.751	6.22E-01	2.436	0.545	5.86E-01	-6.318	11.189	7.78E-01
	R_precentral	-31.120	-3.263	1.11E-03	-49.814	-12.425	4.33E-03	-23.783	-2.072	3.83E-02	-46.284	-1.283	1.58E-01
	R_precuneus	-31.906	-3.381	7.27E-04	-50.403	-13.410	2.99E-03	-29.820	-2.626	8.67E-03	-52.080	-7.559	7.42E-02
	R_rostralanteriorcingulate	0.387	0.141	8.88E-01	-4.991	5.764	9.45E-01	2.487	0.753	4.51E-01	-3.985	8.960	6.80E-01
	R_rostralmiddlefrontal	-52.847	-3.689	2.27E-04	-80.925	-24.770	1.18E-03	-0.308	-0.018	9.86E-01	-34.024	33.409	9.86E-01
	R_superiorfrontal	-27.719	-1.889	5.89E-02	-56.478	1.040	1.17E-01	-20.730	-1.174	2.41E-01	-55.346	13.887	4.61E-01
	R_superiorparietal	-49.995	-3.946	8.05E-05	-74.830	-25.160	5.38E-04	-32.149	-2.109	3.50E-02	-62.032	-2.265	1.47E-01
	R_superiortemporal	-24.810	-3.501	4.66E-04	-38.697	-10.922	2.08E-03	-20.688	-2.426	1.53E-02	-37.404	-3.973	9.37E-02
	R_supramarginal	-39.527	-3.841	1.24E-04	-59.698	-19.357	7.16E-04	-23.922	-1.932	5.35E-02	-48.195	0.351	1.79E-01
	R_transversetemporal	-2.544	-2.394	1.67E-02	-4.626	-0.461	4.31E-02	-3.705	-2.898	3.76E-03	-6.211	-1.200	6.96E-02
	L_bankssts	0.008	1.744	8.12E-02	-0.001	0.017	1.50E-01	0.012	2.123	3.38E-02	0.001	0.023	1.47E-01
	L_caudalanteriorcingulate	-0.004	-0.342	7.32E-01	-0.026	0.018	8.29E-01	0.013	0.913	3.61E-01	-0.014	0.039	5.95E-01
	L_caudalmiddlefrontal	0.012	3.004	2.67E-03	0.004	0.020	9.23E-03	0.011	2.204	2.75E-02	0.001	0.020	1.32E-01
	L_cuneus	0.015	3.825	1.32E-04	0.007	0.022	7.25E-04	0.000	-0.034	9.73E-01	-0.009	0.009	9.86E-01
	L_entorhinal	-0.009	-1.151	2.50E-01	-0.025	0.006	3.63E-01	0.009	0.972	3.31E-01	-0.009	0.028	5.67E-01
	L_frontalpole	0.026	4.235	2.32E-05	0.014	0.038	1.99E-04	0.014	1.903	5.71E-02	0.000	0.029	1.88E-01
**Cortical Mean Thickness**	L_fusiform	0.000	-0.071	9.44E-01	-0.007	0.007	9.75E-01	0.007	1.748	8.04E-02	-0.001	0.015	2.33E-01
	L_inferiorparietal	0.011	3.298	9.79E-04	0.004	0.017	3.95E-03	0.010	2.484	1.30E-02	0.002	0.017	9.37E-02
	L_inferiortemporal	-0.003	-0.914	3.61E-01	-0.011	0.004	4.89E-01	0.002	0.338	7.35E-01	-0.007	0.010	8.94E-01
	L_insula	-0.007	-1.409	1.59E-01	-0.016	0.003	2.50E-01	0.008	1.460	1.44E-01	-0.003	0.019	3.19E-01
	L_isthmuscingulate	0.002	0.537	5.91E-01	-0.007	0.011	7.07E-01	0.002	0.347	7.28E-01	-0.009	0.013	8.91E-01
	L_lateraloccipital	0.022	6.871	7.02E-12	0.016	0.029	7.51E-10	0.011	2.814	4.91E-03	0.003	0.019	6.96E-02
	L_lateralorbitofrontal	0.004	0.987	3.24E-01	-0.004	0.011	4.56E-01	0.008	1.715	8.63E-02	-0.001	0.016	2.36E-01
	L_lingual	0.013	3.506	4.58E-04	0.006	0.021	2.08E-03	0.006	1.359	1.74E-01	-0.003	0.015	3.69E-01

	L_medialorbitofrontal	-0.004	-0.931	3.52E-01	-0.012	0.004	4.82E-01	0.000	0.054	9.57E-01	-0.009	0.010	9.86E-01
	L_middletemporal	0.001	0.260	7.95E-01	-0.007	0.009	8.63E-01	0.010	2.010	4.45E-02	0.000	0.020	1.64E-01
	L_paracentral	0.007	1.280	2.01E-01	-0.003	0.017	3.00E-01	0.014	2.352	1.87E-02	0.002	0.027	1.00E-01
	L_parahippocampal	-0.021	-2.714	6.66E-03	-0.036	-0.006	2.10E-02	-0.007	-0.765	4.44E-01	-0.025	0.011	6.75E-01
	L_parsopercularis	0.007	1.751	8.00E-02	-0.001	0.014	1.49E-01	0.012	2.567	1.03E-02	0.003	0.021	8.47E-02
	L_parsorbitalis	0.019	4.020	5.88E-05	0.010	0.028	4.19E-04	0.015	2.667	7.67E-03	0.004	0.026	7.13E-02
	L_parstriangularis	0.016	4.195	2.77E-05	0.009	0.024	2.28E-04	0.013	2.839	4.53E-03	0.004	0.022	6.96E-02
	L_pericalcarine	0.013	3.596	3.25E-04	0.006	0.021	1.51E-03	0.001	0.189	8.50E-01	-0.008	0.010	9.52E-01
	L_postcentral	0.016	4.099	4.20E-05	0.008	0.023	3.21E-04	0.009	1.887	5.92E-02	0.000	0.018	1.92E-01
	L_posteriorcingulate	-0.002	-0.502	6.15E-01	-0.012	0.007	7.20E-01	0.000	-0.020	9.84E-01	-0.011	0.011	9.86E-01
	L_precentral	0.008	1.674	9.42E-02	-0.001	0.017	1.62E-01	0.013	2.358	1.84E-02	0.002	0.024	1.00E-01
	L_precuneus	0.017	4.692	2.77E-06	0.010	0.024	2.96E-05	0.014	3.280	1.04E-03	0.006	0.023	3.21E-02
	L_rostralanteriorcingulate	-0.004	-0.739	4.60E-01	-0.015	0.007	5.93E-01	0.010	1.523	1.28E-01	-0.003	0.023	3.06E-01
	L_rostralmiddlefrontal	0.021	6.149	8.26E-10	0.014	0.027	3.54E-08	0.014	3.585	3.40E-04	0.006	0.022	2.65E-02
	L_superiorfrontal	0.010	2.609	9.11E-03	0.002	0.017	2.60E-02	0.011	2.433	1.50E-02	0.002	0.020	9.37E-02
	L_superiorparietal	0.021	5.938	3.05E-09	0.014	0.028	1.09E-07	0.013	3.145	1.67E-03	0.005	0.022	4.46E-02
	L_superiortemporal	-0.004	-0.810	4.18E-01	-0.012	0.005	5.55E-01	0.012	2.185	2.89E-02	0.001	0.022	1.34E-01
	L_supramarginal	0.009	2.503	1.23E-02	0.002	0.016	3.38E-02	0.012	2.632	8.51E-03	0.003	0.020	7.42E-02
	L_transversetemporal	-0.005	-0.743	4.58E-01	-0.017	0.008	5.93E-01	-0.001	-0.094	9.25E-01	-0.016	0.014	9.86E-01
	R_bankssts	0.009	1.775	7.59E-02	-0.001	0.018	1.44E-01	0.010	1.760	7.84E-02	-0.001	0.022	2.33E-01
	R_caudalanteriorcingulate	0.018	2.051	4.03E-02	0.001	0.036	8.54E-02	0.019	1.739	8.20E-02	-0.002	0.040	2.33E-01
	R_caudalmiddlefrontal	0.011	2.851	4.37E-03	0.004	0.019	1.44E-02	0.007	1.520	1.29E-01	-0.002	0.017	3.06E-01
	R_cuneus	0.017	4.486	7.37E-06	0.009	0.024	6.86E-05	0.001	0.257	7.97E-01	-0.008	0.010	9.28E-01
	R_entorhinal	-0.007	-0.824	4.10E-01	-0.024	0.010	5.49E-01	-0.006	-0.589	5.56E-01	-0.026	0.014	7.63E-01
	R_frontalpole	0.030	4.911	9.30E-07	0.018	0.041	1.42E-05	0.014	1.989	4.67E-02	0.000	0.029	1.68E-01
	R_fusiform	0.006	1.636	1.02E-01	-0.001	0.013	1.73E-01	0.010	2.289	2.21E-02	0.001	0.019	1.15E-01
	R_inferiorparietal	0.016	4.730	2.30E-06	0.009	0.023	2.59E-05	0.009	2.234	2.55E-02	0.001	0.017	1.30E-01
	R_inferiortemporal	0.006	1.685	9.21E-02	-0.001	0.013	1.60E-01	0.008	1.822	6.86E-02	-0.001	0.016	2.12E-01
	R_insula	-0.003	-0.631	5.28E-01	-0.012	0.006	6.53E-01	0.000	0.079	9.37E-01	-0.011	0.011	9.86E-01
	R_isthmuscingulate	0.008	1.731	8.34E-02	-0.001	0.018	1.51E-01	0.009	1.583	1.13E-01	-0.002	0.021	2.86E-01
	R_lateraloccipital	0.024	6.888	6.23E-12	0.017	0.030	7.51E-10	0.007	1.657	9.76E-02	-0.001	0.015	2.55E-01
	R_lateralorbitofrontal	0.005	1.417	1.57E-01	-0.002	0.013	2.48E-01	0.008	1.744	8.12E-02	-0.001	0.017	2.33E-01
	R_lingual	0.019	5.087	3.75E-07	0.012	0.026	8.74E-06	0.007	1.452	1.47E-01	-0.002	0.015	3.20E-01
	R_medialorbitofrontal	0.005	1.198	2.31E-01	-0.003	0.013	3.41E-01	0.008	1.710	8.73E-02	-0.001	0.018	2.36E-01
	R_middletemporal	0.013	3.262	1.11E-03	0.005	0.020	4.33E-03	0.013	2.677	7.44E-03	0.003	0.022	7.13E-02
	R_paracentral	0.007	1.447	1.48E-01	-0.003	0.017	2.38E-01	0.010	1.732	8.34E-02	-0.001	0.022	2.33E-01
	R_parahippocampal	-0.004	-0.546	5.85E-01	-0.016	0.009	7.04E-01	0.002	0.277	7.82E-01	-0.013	0.018	9.24E-01
	R_parsopercularis	0.010	2.647	8.14E-03	0.003	0.018	2.42E-02	0.010	2.220	2.64E-02	0.001	0.020	1.32E-01
	R_parsorbitalis	0.021	4.556	5.33E-06	0.012	0.030	5.18E-05	0.016	2.977	2.92E-03	0.006	0.027	6.95E-02
	R_parstriangularis	0.018	4.924	8.70E-07	0.011	0.025	1.42E-05	0.015	3.435	5.95E-04	0.007	0.024	2.65E-02
	R_pericalcarine	0.015	4.175	3.02E-05	0.008	0.023	2.39E-04	-0.001	-0.278	7.81E-01	-0.010	0.007	9.24E-01
	R_postcentral	0.019	4.781	1.78E-06	0.011	0.026	2.16E-05	0.010	2.031	4.23E-02	0.000	0.019	1.61E-01
	R_posteriorcingulate	0.007	1.511	1.31E-01	-0.002	0.017	2.14E-01	0.008	1.387	1.66E-01	-0.003	0.019	3.58E-01
	R_precentral	0.011	2.428	1.52E-02	0.002	0.021	4.02E-02	0.010	1.729	8.39E-02	-0.001	0.021	2.33E-01
	R_precuneus	0.019	5.320	1.07E-07	0.012	0.026	3.27E-06	0.015	3.425	6.19E-04	0.006	0.023	2.65E-02
	R_rostralanteriorcingulate	0.003	0.526	5.99E-01	-0.008	0.014	7.12E-01	0.008	1.172	2.41E-01	-0.005	0.022	4.61E-01

	R_rostralmiddlefrontal	0.022	6.755	1.56E-11	0.015	0.028	8.35E-10	0.011	2.730	6.34E-03	0.003	0.018	6.96E-02
	R_superiorfrontal	0.015	3.953	7.80E-05	0.007	0.022	5.38E-04	0.011	2.402	1.63E-02	0.002	0.020	9.70E-02
	R_superiorparietal	0.024	6.773	1.37E-11	0.017	0.031	8.35E-10	0.012	2.813	4.93E-03	0.004	0.020	6.96E-02
	R_superiortemporal	0.003	0.614	5.39E-01	-0.006	0.011	6.63E-01	0.010	2.034	4.20E-02	0.000	0.020	1.61E-01
	R_supramarginal	0.014	3.789	1.53E-04	0.007	0.022	8.18E-04	0.013	2.815	4.90E-03	0.004	0.021	6.96E-02
	R_transversetemporal	-0.002	-0.292	7.70E-01	-0.015	0.011	8.59E-01	-0.001	-0.169	8.66E-01	-0.017	0.014	9.65E-01
**Cortical Volume**	L_bankssts	-13.911	-1.328	1.84E-01	-34.444	6.622	2.84E-01	10.389	0.826	4.09E-01	-14.276	35.054	6.48E-01
	L_caudalanteriorcingulate	1.042	0.094	9.25E-01	-20.613	22.696	9.70E-01	0.731	0.055	9.56E-01	-25.337	26.798	9.86E-01
	L_caudalmiddlefrontal	-9.610	-0.484	6.28E-01	-48.516	29.296	7.31E-01	12.154	0.509	6.11E-01	-34.634	58.943	7.90E-01
	L_cuneus	-11.175	-0.920	3.58E-01	-34.986	12.636	4.88E-01	-19.996	-1.369	1.71E-01	-48.629	8.637	3.66E-01
	L_entorhinal	-4.146	-0.556	5.78E-01	-18.756	10.464	6.99E-01	9.571	1.067	2.86E-01	-8.008	27.151	5.15E-01
	L_frontalpole	-1.972	-0.876	3.81E-01	-6.385	2.441	5.13E-01	-2.915	-1.076	2.82E-01	-8.222	2.392	5.15E-01
	L_fusiform	-29.610	-1.368	1.71E-01	-72.042	12.823	2.68E-01	3.762	0.145	8.85E-01	-47.250	54.773	9.79E-01
	L_inferiorparietal	-86.699	-2.461	1.39E-02	-155.749	-17.650	3.71E-02	4.111	0.097	9.23E-01	-78.789	87.012	9.86E-01
	L_inferiortemporal	-109.727	-4.024	5.80E-05	-163.175	-56.278	4.19E-04	0.727	0.022	9.82E-01	-63.323	64.776	9.86E-01
	L_insula	-23.213	-1.532	1.26E-01	-52.918	6.492	2.10E-01	-10.569	-0.580	5.62E-01	-46.304	25.166	7.65E-01
	L_isthmuscingulate	13.695	1.717	8.61E-02	-1.939	29.330	1.52E-01	14.853	1.547	1.22E-01	-3.971	33.678	3.00E-01
	L_lateraloccipital	18.601	0.580	5.62E-01	-44.230	81.432	6.86E-01	38.343	0.994	3.20E-01	-37.256	113.941	5.53E-01
	L_lateralorbitofrontal	-24.795	-1.673	9.45E-02	-53.851	4.261	1.62E-01	-2.424	-0.136	8.92E-01	-37.339	32.490	9.79E-01
	L_lingual	15.893	0.700	4.84E-01	-28.625	60.412	6.13E-01	17.300	0.633	5.27E-01	-36.269	70.868	7.43E-01
	L_medialorbitofrontal	-40.559	-3.174	1.51E-03	-65.602	-15.517	5.47E-03	-11.309	-0.736	4.62E-01	-41.412	18.793	6.90E-01
	L_middletemporal	-46.437	-1.888	5.91E-02	-94.647	1.774	1.17E-01	43.604	1.478	1.39E-01	-14.209	101.417	3.17E-01
	L_paracentral	0.589	0.060	9.52E-01	-18.754	19.932	9.80E-01	9.595	0.809	4.19E-01	-13.658	32.848	6.58E-01
	L_parahippocampal	-14.320	-2.293	2.19E-02	-26.557	-2.082	5.16E-02	-1.593	-0.212	8.32E-01	-16.311	13.124	9.42E-01
	L_parsopercularis	-37.982	-2.467	1.36E-02	-68.154	-7.811	3.69E-02	-33.044	-1.784	7.44E-02	-69.343	3.254	2.24E-01
	L_parsorbitalis	0.197	0.035	9.72E-01	-10.801	11.195	9.86E-01	12.247	1.816	6.94E-02	-0.970	25.464	2.12E-01
	L_parstriangularis	-17.931	-1.560	1.19E-01	-40.455	4.593	2.00E-01	-14.516	-1.051	2.93E-01	-41.592	12.561	5.15E-01
	L_pericalcarine	-3.248	-0.272	7.86E-01	-26.697	20.200	8.63E-01	-9.784	-0.680	4.97E-01	-37.999	18.430	7.18E-01
	L_postcentral	-29.359	-1.234	2.17E-01	-75.988	17.270	3.23E-01	6.794	0.238	8.12E-01	-49.257	62.846	9.40E-01
	L_posteriorcingulate	-0.171	-0.017	9.86E-01	-19.800	19.458	9.87E-01	7.264	0.603	5.47E-01	-16.361	30.889	7.63E-01
	L_precentral	16.732	0.576	5.64E-01	-40.171	73.636	6.86E-01	68.024	1.952	5.10E-02	-0.276	136.323	1.73E-01
	L_precuneus	-8.069	-0.331	7.40E-01	-55.780	39.643	8.29E-01	-9.433	-0.322	7.47E-01	-66.804	47.939	8.98E-01
	L_rostralanteriorcingulate	7.681	0.790	4.29E-01	-11.364	26.726	5.67E-01	-1.501	-0.128	8.98E-01	-24.423	21.420	9.80E-01
	L_rostralmiddlefrontal	-71.315	-1.993	4.63E-02	-141.455	-1.176	9.53E-02	-11.166	-0.260	7.95E-01	-95.450	73.117	9.28E-01
	L_superiorfrontal	-42.539	-0.969	3.33E-01	-128.613	43.534	4.65E-01	-11.592	-0.220	8.26E-01	-114.830	91.646	9.42E-01
	L_superiorparietal	-58.977	-1.719	8.57E-02	-126.237	8.283	1.52E-01	-29.667	-0.719	4.72E-01	-110.493	51.160	6.96E-01
	L_superiortemporal	-108.399	-3.886	1.03E-04	-163.071	-53.727	6.48E-04	-49.521	-1.480	1.39E-01	-115.110	16.068	3.17E-01
	L_supramarginal	-83.192	-2.410	1.60E-02	-150.844	-15.540	4.17E-02	-13.417	-0.323	7.46E-01	-94.707	67.873	8.98E-01
	L_transversetemporal	-9.985	-2.378	1.74E-02	-18.215	-1.755	4.34E-02	-12.236	-2.425	1.53E-02	-22.124	-2.348	9.37E-02
	R_bankssts	-9.995	-1.119	2.63E-01	-27.507	7.516	3.78E-01	11.863	1.107	2.69E-01	-9.150	32.877	5.00E-01
	R_caudalanteriorcingulate	12.335	1.123	2.61E-01	-9.191	33.860	3.78E-01	11.221	0.849	3.96E-01	-14.688	37.130	6.32E-01
	R_caudalmiddlefrontal	-45.974	-2.263	2.37E-02	-85.799	-6.149	5.45E-02	-31.659	-1.295	1.95E-01	-79.560	16.241	3.94E-01
	R_cuneus	-11.174	-0.945	3.45E-01	-34.359	12.011	4.76E-01	-24.007	-1.687	9.16E-02	-51.894	3.881	2.42E-01
	R_entorhinal	-12.882	-2.042	4.12E-02	-25.247	-0.517	8.64E-02	-8.055	-1.061	2.89E-01	-22.939	6.829	5.15E-01
	R_frontalpole	10.584	3.930	8.60E-05	5.305	15.863	5.58E-04	5.472	1.691	9.09E-02	-0.871	11.815	2.42E-01
	R_fusiform	-45.324	-2.117	3.43E-02	-87.283	-3.365	7.41E-02	-26.858	-1.044	2.96E-01	-77.276	23.561	5.16E-01

	R_inferiorparietal	13.305	0.332	7.40E-01	-65.307	91.918	8.29E-01	70.970	1.473	1.41E-01	-23.476	165.415	3.17E-01
	R_inferiortemporal	-55.947	-2.204	2.76E-02	-105.706	-6.189	6.17E-02	1.996	0.065	9.48E-01	-57.797	61.788	9.86E-01
	R_insula	0.432	0.027	9.79E-01	-31.343	32.207	9.87E-01	1.448	0.074	9.41E-01	-36.777	39.672	9.86E-01
	R_isthmuscingulate	13.326	1.766	7.74E-02	-1.462	28.114	1.45E-01	4.760	0.524	6.00E-01	-13.031	22.551	7.84E-01
	R_lateraloccipital	48.772	1.453	1.46E-01	-17.030	114.573	2.37E-01	-14.678	-0.364	7.16E-01	-93.808	64.451	8.83E-01
	R_lateralorbitofrontal	-35.781	-2.297	2.16E-02	-66.311	-5.251	5.16E-02	-3.582	-0.192	8.48E-01	-40.200	33.035	9.52E-01
	R_lingual	26.092	1.073	2.83E-01	-21.575	73.759	4.04E-01	0.839	0.029	9.77E-01	-56.511	58.189	9.86E-01
	R_medialorbitofrontal	-19.144	-1.723	8.50E-02	-40.922	2.634	1.52E-01	-2.944	-0.221	8.25E-01	-29.087	23.199	9.42E-01
	R_middletemporal	2.437	0.100	9.20E-01	-45.361	50.236	9.70E-01	58.108	1.986	4.71E-02	0.754	115.462	1.68E-01
	R_paracentral	25.435	2.202	2.77E-02	2.799	48.072	6.17E-02	27.211	1.960	5.01E-02	-0.001	54.423	1.73E-01
	R_parahippocampal	-3.822	-0.727	4.67E-01	-14.128	6.484	5.99E-01	5.946	0.941	3.47E-01	-6.442	18.333	5.81E-01
	R_parsopercularis	-18.401	-1.511	1.31E-01	-42.262	5.460	2.14E-01	-15.494	-1.058	2.90E-01	-44.208	13.220	5.15E-01
	R_parsorbitalis	-4.488	-0.664	5.07E-01	-17.738	8.763	6.31E-01	8.983	1.106	2.69E-01	-6.941	24.907	5.00E-01
	R_parstriangularis	0.222	0.016	9.87E-01	-27.351	27.795	9.87E-01	-2.320	-0.137	8.91E-01	-35.500	30.860	9.79E-01
	R_pericalcarine	-1.091	-0.086	9.32E-01	-26.019	23.837	9.73E-01	-17.515	-1.145	2.52E-01	-47.498	12.469	4.78E-01
	R_postcentral	-23.011	-0.962	3.36E-01	-69.897	23.875	4.67E-01	-16.186	-0.563	5.73E-01	-72.533	40.161	7.72E-01
	R_posteriorcingulate	1.910	0.191	8.49E-01	-17.697	21.517	9.08E-01	15.791	1.313	1.89E-01	-7.788	39.370	3.87E-01
	R_precentral	-30.109	-1.004	3.16E-01	-88.900	28.682	4.47E-01	-13.968	-0.387	6.98E-01	-84.628	56.693	8.69E-01
	R_precuneus	-11.686	-0.475	6.35E-01	-59.940	36.567	7.35E-01	-20.557	-0.695	4.87E-01	-78.567	37.453	7.11E-01
	R_rostralanteriorcingulate	3.376	0.411	6.81E-01	-12.731	19.482	7.80E-01	13.241	1.339	1.81E-01	-6.140	32.622	3.75E-01
	R_rostralmiddlefrontal	-17.162	-0.447	6.55E-01	-92.394	58.070	7.53E-01	57.906	1.256	2.09E-01	-32.441	148.253	4.11E-01
	R_superiorfrontal	16.780	0.380	7.04E-01	-69.728	103.287	8.01E-01	16.014	0.302	7.62E-01	-87.823	119.850	9.12E-01
	R_superiorparietal	6.350	0.195	8.45E-01	-57.358	70.058	9.08E-01	-14.091	-0.361	7.18E-01	-90.628	62.446	8.83E-01
	R_superiortemporal	-64.251	-2.578	9.96E-03	-113.101	-15.401	2.81E-02	-22.864	-0.764	4.45E-01	-81.503	35.775	6.75E-01
	R_supramarginal	-62.300	-2.128	3.34E-02	-119.682	-4.918	7.36E-02	-22.745	-0.646	5.18E-01	-91.736	46.247	7.39E-01
	R_transversetemporal	-7.037	-2.387	1.70E-02	-12.815	-1.259	4.32E-02	-9.649	-2.722	6.51E-03	-16.596	-2.701	6.96E-02
**Subcortical Volume**	Laccumb	4.978	2.621	8.78E-03	1.256	8.699	2.54E-02	3.540	1.551	1.21E-01	-0.934	8.014	3.00E-01
	Lamyg	20.927	4.572	4.92E-06	11.956	29.897	5.01E-05	15.058	2.738	6.20E-03	4.279	25.837	6.96E-02
	Lcaud	13.183	1.360	1.74E-01	-5.813	32.179	2.70E-01	-1.232	-0.106	9.16E-01	-24.068	21.604	9.86E-01
	Lhippo	30.501	3.659	2.55E-04	14.165	46.837	1.24E-03	24.975	2.494	1.27E-02	5.347	44.602	9.37E-02
	LLatVent	-302.555	-1.786	7.42E-02	-634.609	29.499	1.42E-01	-416.461	-2.046	4.08E-02	-815.413	-17.510	1.61E-01
	Lpal	-0.601	-0.129	8.98E-01	-9.747	8.546	9.51E-01	15.597	2.785	5.37E-03	4.620	26.574	6.96E-02
	Lput	54.678	4.828	1.41E-06	32.483	76.874	1.89E-05	29.399	2.160	3.08E-02	2.727	56.070	1.40E-01
	Lthal	38.483	2.993	2.77E-03	13.284	63.683	9.41E-03	58.353	3.779	1.59E-04	28.085	88.621	2.65E-02
	Raccumb	4.887	2.707	6.82E-03	1.348	8.426	2.11E-02	3.981	1.836	6.64E-02	-0.269	8.232	2.09E-01
	Ramyg	21.812	4.829	1.40E-06	12.960	30.664	1.89E-05	13.605	2.506	1.22E-02	2.964	24.246	9.37E-02
	Rcaud	22.366	2.292	2.19E-02	3.240	41.491	5.16E-02	6.526	0.556	5.78E-01	-16.475	29.528	7.73E-01
	Rhippo	32.243	3.663	2.52E-04	14.990	49.497	1.24E-03	23.280	2.200	2.78E-02	2.541	44.020	1.32E-01
	RLatVent	-250.529	-1.732	8.33E-02	-533.971	32.913	1.51E-01	-276.428	-1.591	1.12E-01	-617.049	64.193	2.85E-01
	Rpal	-0.226	-0.050	9.60E-01	-9.114	8.663	9.83E-01	14.564	2.675	7.50E-03	3.892	25.236	7.13E-02
	Rput	41.856	3.670	2.45E-04	19.502	64.210	1.24E-03	20.097	1.466	1.43E-01	-6.778	46.973	3.18E-01
	Rthal	61.731	5.210	1.95E-07	38.507	84.955	5.22E-06	50.341	3.534	4.13E-04	22.420	78.262	2.65E-02

Supplementary Table 5: The association between hearing difficulty without tinnitus (hearing difficulty only), mean thickness and volume in the UK Biobank. Values highlighted in red are the significant values at respective P-value and corrected P-value at 0.05. ICV: intracranial volume; CVD: cardiovascular disease; BMI: body mass index; TDI, Townsend deprivation index; ROI: region of interest; IDP: imaging derived phenotypes; CI: confidence interval; FDR: false discovery rate.

Cases (4,683) vs. Controls (4,683): Hdiff_only vs. Control	IDP	Model 1: IDP ~ Hdiff_only + Age + Sex + ICV						Model 2: IDP ~ Hdiff_only + Age + Sex + ICV + Smoking + Alcohol + Diabetes + CVD + BMI +TDI					
		Estimate	T-value	P-value	Lower-CI	Upper-CI	FDR	Estimate	T-value	P-value	Lower-CI	Upper-CI	FDR
Cortical Surface Area	L_bankssts	-12.487	-4.374	1.23E-05	-18.083	-6.892	2.39E-04	-8.208	-2.621	8.78E-03	-14.346	-2.070	2.35E-01
	L_caudalanteriorcingulate	-2.606	-1.025	3.05E-01	-7.589	2.376	4.66E-01	-5.295	-1.897	5.78E-02	-10.764	0.175	3.64E-01
	L_caudalmiddlefrontal	-9.296	-1.624	1.04E-01	-20.514	1.923	2.23E-01	-0.844	-0.134	8.93E-01	-13.154	11.466	9.46E-01
	L_cuneus	-10.763	-2.604	9.22E-03	-18.863	-2.663	3.79E-02	0.497	0.110	9.13E-01	-8.378	9.371	9.57E-01
	L_entorhinal	-1.784	-1.000	3.17E-01	-5.279	1.712	4.75E-01	-1.783	-0.911	3.62E-01	-5.620	2.053	6.30E-01
	L_frontalpole	-0.373	-0.696	4.86E-01	-1.425	0.678	6.34E-01	0.153	0.259	7.95E-01	-1.001	1.306	9.20E-01
	L_fusiform	-10.544	-1.867	6.19E-02	-21.614	0.525	1.49E-01	-7.110	-1.147	2.51E-01	-19.259	5.038	5.53E-01
	L_inferiorparietal	-43.215	-4.151	3.33E-05	-63.618	-22.812	5.09E-04	-16.779	-1.471	1.41E-01	-39.130	5.572	4.65E-01
	L_inferiortemporal	-26.244	-3.630	2.85E-04	-40.415	-12.073	2.91E-03	-9.335	-1.178	2.39E-01	-24.870	6.200	5.48E-01
	L_insula	4.015	0.976	3.29E-01	-4.046	12.077	4.79E-01	-1.729	-0.383	7.02E-01	-10.576	7.117	8.52E-01
	L_isthmuscingulate	5.790	2.127	3.34E-02	0.455	11.124	1.02E-01	3.688	1.234	2.17E-01	-2.168	9.544	5.48E-01
	L_lateraloccipital	-3.287	-0.319	7.50E-01	-23.506	16.932	8.36E-01	17.746	1.569	1.17E-01	-4.425	39.916	4.35E-01
	L_lateralorbitofrontal	-5.485	-1.358	1.74E-01	-13.400	2.430	3.11E-01	-2.443	-0.551	5.82E-01	-11.129	6.244	7.54E-01
	L_lingual	-4.662	-0.628	5.30E-01	-19.220	9.897	6.56E-01	10.818	1.328	1.84E-01	-5.144	26.780	5.31E-01
	L_medialorbitofrontal	-9.536	-2.672	7.56E-03	-16.531	-2.540	3.30E-02	-6.684	-1.706	8.80E-02	-14.363	0.995	4.10E-01
	L_middletemporal	-21.547	-3.523	4.29E-04	-33.535	-9.560	3.53E-03	-6.468	-0.965	3.35E-01	-19.605	6.669	6.02E-01
	L_paracentral	0.393	0.140	8.88E-01	-5.095	5.881	9.40E-01	-0.251	-0.082	9.35E-01	-6.275	5.773	9.66E-01
	L_parahippocampal	1.127	0.831	4.06E-01	-1.531	3.785	5.50E-01	1.967	1.322	1.86E-01	-0.950	4.884	5.31E-01
	L_parsopercularis	-4.205	-0.990	3.22E-01	-12.529	4.119	4.79E-01	-1.365	-0.293	7.70E-01	-10.502	7.771	9.00E-01
	L_parsorbitalis	-1.440	-0.984	3.25E-01	-4.310	1.429	4.79E-01	1.190	0.741	4.59E-01	-1.957	4.337	6.59E-01
	L_parstriangularis	-2.914	-0.857	3.91E-01	-9.576	3.747	5.37E-01	0.939	0.252	8.01E-01	-6.371	8.249	9.20E-01
	L_pericalcarine	-6.991	-1.426	1.54E-01	-16.603	2.621	2.89E-01	4.134	0.769	4.42E-01	-6.404	14.672	6.59E-01
	L_postcentral	-30.935	-4.484	7.42E-06	-44.457	-17.413	1.59E-04	-11.830	-1.565	1.18E-01	-26.642	2.982	4.35E-01
	L_posteriorcingulate	-4.212	-1.359	1.74E-01	-10.285	1.861	3.11E-01	-3.571	-1.050	2.94E-01	-10.238	3.096	5.91E-01
	L_precentral	-15.549	-2.043	4.11E-02	-30.468	-0.630	1.16E-01	-9.313	-1.115	2.65E-01	-25.683	7.057	5.53E-01
	L_precuneus	-17.523	-2.338	1.94E-02	-32.215	-2.831	6.82E-02	-11.464	-1.393	1.64E-01	-27.589	4.662	5.00E-01
	L_rostralanteriorcingulate	5.063	1.774	7.61E-02	-0.531	10.656	1.77E-01	1.233	0.394	6.94E-01	-4.903	7.369	8.48E-01
	L_rostralmiddlefrontal	-30.109	-2.776	5.51E-03	-51.364	-8.854	2.56E-02	0.655	0.055	9.56E-01	-22.629	23.939	9.67E-01
	L_superiorfrontal	-17.814	-1.476	1.40E-01	-41.475	5.847	2.74E-01	-9.713	-0.733	4.64E-01	-35.687	16.260	6.59E-01
	L_superiorparietal	-37.295	-3.376	7.39E-04	-58.948	-15.642	4.94E-03	-7.996	-0.661	5.09E-01	-31.709	15.717	6.93E-01
	L_superiortemporal	-20.195	-3.022	2.52E-03	-33.294	-7.095	1.36E-02	-11.367	-1.550	1.21E-01	-25.743	3.009	4.35E-01
	L_supramarginal	-30.410	-2.966	3.03E-03	-50.506	-10.313	1.58E-02	-14.254	-1.268	2.05E-01	-36.295	7.787	5.48E-01
	L_transversetemporal	-0.859	-0.648	5.17E-01	-3.456	1.738	6.48E-01	-1.820	-1.252	2.11E-01	-4.671	1.030	5.48E-01
	R_bankssts	-6.844	-3.100	1.94E-03	-11.170	-2.517	1.13E-02	-3.679	-1.519	1.29E-01	-8.425	1.067	4.43E-01

	R_caudalanteriorcingulate	-0.463	-0.159	8.74E-01	-6.177	5.250	9.35E-01	0.126	0.039	9.69E-01	-6.146	6.397	9.73E-01
	R_caudalmiddlefrontal	-17.501	-3.020	2.54E-03	-28.861	-6.142	1.36E-02	-11.390	-1.790	7.34E-02	-23.860	1.079	3.83E-01
	R_cuneus	-6.976	-1.691	9.10E-02	-15.064	1.112	1.99E-01	3.334	0.737	4.61E-01	-5.529	12.197	6.59E-01
	R_entorhinal	-0.080	-0.054	9.57E-01	-3.003	2.843	9.71E-01	0.348	0.213	8.32E-01	-2.860	3.556	9.30E-01
	R_frontalpole	-1.132	-1.703	8.86E-02	-2.435	0.171	1.96E-01	-0.394	-0.539	5.90E-01	-1.823	1.036	7.60E-01
	R_fusiform	-10.820	-1.901	5.74E-02	-21.978	0.337	1.41E-01	-10.072	-1.612	1.07E-01	-22.319	2.176	4.35E-01
	R_inferiorparietal	-41.365	-3.441	5.83E-04	-64.927	-17.802	4.62E-03	-8.975	-0.682	4.96E-01	-34.784	16.834	6.84E-01
	R_inferiortemporal	-25.417	-3.765	1.68E-04	-38.649	-12.185	1.81E-03	-16.385	-2.212	2.70E-02	-30.905	-1.865	3.39E-01
	R_insula	5.005	1.065	2.87E-01	-4.206	14.216	4.48E-01	0.454	0.088	9.30E-01	-9.654	10.563	9.66E-01
	R_isthmuscingulate	2.247	0.916	3.60E-01	-2.562	7.056	5.10E-01	0.599	0.222	8.24E-01	-4.681	5.878	9.28E-01
	R_lateraloccipital	-21.140	-1.939	5.26E-02	-42.512	0.233	1.36E-01	-8.145	-0.681	4.96E-01	-31.584	15.293	6.84E-01
	R_lateralorbitofrontal	-7.182	-1.473	1.41E-01	-16.741	2.377	2.74E-01	-3.032	-0.566	5.71E-01	-13.522	7.459	7.45E-01
	R_lingual	-4.055	-0.508	6.11E-01	-19.692	11.582	7.27E-01	13.686	1.565	1.18E-01	-3.454	30.825	4.35E-01
	R_medialorbitofrontal	-4.267	-1.339	1.81E-01	-10.512	1.979	3.17E-01	-2.783	-0.795	4.26E-01	-9.639	4.073	6.59E-01
	R_middletemporal	-23.004	-3.837	1.25E-04	-34.755	-11.253	1.49E-03	-13.172	-2.003	4.52E-02	-26.060	-0.285	3.39E-01
	R_paracentral	5.716	1.721	8.53E-02	-0.794	12.225	1.92E-01	4.252	1.166	2.44E-01	-2.895	11.398	5.49E-01
	R_parahippocampal	0.142	0.108	9.14E-01	-2.424	2.707	9.40E-01	1.619	1.127	2.60E-01	-1.197	4.434	5.53E-01
	R_parsopercularis	1.178	0.338	7.35E-01	-5.656	8.013	8.24E-01	3.446	0.900	3.68E-01	-4.057	10.948	6.30E-01
	R_parsorbitalis	-3.989	-2.233	2.56E-02	-7.490	-0.488	8.55E-02	-0.664	-0.339	7.35E-01	-4.501	3.174	8.69E-01
	R_parstriangularis	0.116	0.028	9.78E-01	-8.008	8.240	9.80E-01	3.532	0.776	4.38E-01	-5.384	12.447	6.59E-01
	R_pericalcarine	-4.932	-0.948	3.43E-01	-15.127	5.264	4.94E-01	8.620	1.512	1.31E-01	-2.553	19.792	4.43E-01
	R_postcentral	-34.072	-4.834	1.36E-06	-47.885	-20.258	4.47E-05	-17.248	-2.234	2.55E-02	-32.382	-2.114	3.39E-01
	R_posteriorcingulate	-6.618	-2.081	3.74E-02	-12.849	-0.386	1.09E-01	-5.098	-1.461	1.44E-01	-11.938	1.742	4.67E-01
	R_precentral	-12.152	-1.570	1.16E-01	-27.321	3.017	2.42E-01	-1.976	-0.233	8.16E-01	-18.622	14.670	9.24E-01
	R_precuneus	-15.023	-1.960	5.00E-02	-30.044	-0.002	1.31E-01	-6.909	-0.822	4.11E-01	-23.393	9.574	6.49E-01
	R_rostralanteriorcingulate	1.899	0.842	4.00E-01	-2.521	6.319	5.45E-01	2.111	0.852	3.94E-01	-2.742	6.964	6.40E-01
	R_rostralmiddlefrontal	-48.600	-4.159	3.23E-05	-71.504	-25.696	5.09E-04	-14.541	-1.136	2.56E-01	-39.625	10.543	5.53E-01
	R_superiorfrontal	-20.327	-1.678	9.33E-02	-44.064	3.409	2.02E-01	-15.673	-1.179	2.38E-01	-41.729	10.383	5.48E-01
	R_superiorparietal	-18.386	-1.766	7.74E-02	-38.789	2.018	1.78E-01	8.695	0.762	4.46E-01	-13.656	31.046	6.59E-01
	R_superiortemporal	-15.365	-2.654	7.97E-03	-26.712	-4.017	3.41E-02	-12.132	-1.908	5.64E-02	-24.591	0.327	3.64E-01
	R_supramarginal	-24.387	-2.848	4.41E-03	-41.169	-7.605	2.14E-02	-17.465	-1.858	6.32E-02	-35.885	0.956	3.75E-01
	R_transversetemporal	-2.503	-2.883	3.95E-03	-4.205	-0.801	2.01E-02	-2.965	-3.111	1.87E-03	-4.833	-1.097	1.33E-01
**Cortical Mean Thickness**	L_bankssts	0.003	0.671	5.03E-01	-0.005	0.010	6.44E-01	0.003	0.784	4.33E-01	-0.005	0.011	6.59E-01
	L_caudalanteriorcingulate	0.007	0.765	4.44E-01	-0.011	0.025	5.90E-01	0.011	1.119	2.63E-01	-0.009	0.031	5.53E-01
	L_caudalmiddlefrontal	0.004	1.118	2.64E-01	-0.003	0.010	4.21E-01	0.003	0.846	3.98E-01	-0.004	0.010	6.40E-01
	L_cuneus	0.015	4.813	1.51E-06	0.009	0.021	4.47E-05	0.005	1.568	1.17E-01	-0.001	0.012	4.35E-01
	L_entorhinal	-0.003	-0.458	6.47E-01	-0.016	0.010	7.60E-01	0.012	1.735	8.28E-02	-0.002	0.026	3.94E-01
	L_frontalpole	0.010	1.899	5.76E-02	0.000	0.020	1.41E-01	-0.001	-0.092	9.27E-01	-0.011	0.010	9.66E-01
	L_fusiform	-0.005	-1.897	5.79E-02	-0.011	0.000	1.41E-01	0.000	-0.137	8.91E-01	-0.007	0.006	9.46E-01
	L_inferiorparietal	0.004	1.431	1.52E-01	-0.001	0.009	2.89E-01	0.002	0.516	6.06E-01	-0.004	0.007	7.70E-01
	L_inferiortemporal	0.000	-0.025	9.80E-01	-0.006	0.006	9.80E-01	0.002	0.527	5.98E-01	-0.005	0.008	7.66E-01
	L_insula	-0.006	-1.542	1.23E-01	-0.013	0.002	2.51E-01	0.003	0.731	4.65E-01	-0.005	0.011	6.59E-01
	L_isthmuscingulate	0.001	0.209	8.35E-01	-0.007	0.008	9.02E-01	-0.001	-0.237	8.13E-01	-0.009	0.007	9.24E-01
	L_lateraloccipital	0.015	5.553	2.89E-08	0.010	0.020	3.09E-06	0.005	1.882	5.99E-02	0.000	0.011	3.66E-01
	L_lateralorbitofrontal	0.002	0.540	5.89E-01	-0.004	0.008	7.05E-01	0.004	1.300	1.93E-01	-0.002	0.011	5.31E-01
	L_lingual	0.011	3.592	3.30E-04	0.005	0.017	3.07E-03	0.005	1.404	1.60E-01	-0.002	0.011	4.98E-01

	L_medialorbitofrontal	0.000	0.114	9.09E-01	-0.006	0.007	9.40E-01	0.003	0.819	4.13E-01	-0.004	0.010	6.49E-01
	L_middletemporal	-0.001	-0.246	8.06E-01	-0.008	0.006	8.75E-01	0.001	0.368	7.13E-01	-0.006	0.009	8.52E-01
	L_paracentral	-0.001	-0.248	8.04E-01	-0.009	0.007	8.75E-01	0.004	0.980	3.27E-01	-0.004	0.013	6.00E-01
	L_parahippocampal	-0.013	-2.007	4.47E-02	-0.025	0.000	1.23E-01	-0.005	-0.711	4.77E-01	-0.019	0.009	6.72E-01
	L_parsopercularis	0.003	0.918	3.59E-01	-0.003	0.009	5.10E-01	0.003	0.883	3.77E-01	-0.004	0.010	6.40E-01
	L_parsorbitalis	0.009	2.415	1.58E-02	0.002	0.017	5.64E-02	0.009	2.066	3.89E-02	0.000	0.017	3.39E-01
	L_parstriangularis	0.004	1.141	2.54E-01	-0.003	0.010	4.09E-01	0.002	0.512	6.08E-01	-0.005	0.009	7.70E-01
	L_pericalcarine	0.011	3.838	1.25E-04	0.006	0.017	1.49E-03	0.003	0.875	3.82E-01	-0.004	0.009	6.40E-01
	L_postcentral	0.010	3.133	1.73E-03	0.004	0.016	1.06E-02	0.003	0.846	3.98E-01	-0.004	0.009	6.40E-01
	L_posteriorcingulate	0.000	0.126	9.00E-01	-0.007	0.008	9.40E-01	0.001	0.178	8.59E-01	-0.008	0.009	9.33E-01
	L_precentral	0.003	0.814	4.16E-01	-0.004	0.011	5.59E-01	0.005	1.173	2.41E-01	-0.003	0.013	5.48E-01
	L_precuneus	0.008	2.542	1.10E-02	0.002	0.013	4.30E-02	0.005	1.477	1.40E-01	-0.002	0.011	4.65E-01
	L_rostralanteriorcingulate	-0.002	-0.365	7.15E-01	-0.011	0.007	8.05E-01	0.004	0.759	4.48E-01	-0.006	0.014	6.59E-01
	L_rostralmiddlefrontal	0.008	3.058	2.23E-03	0.003	0.014	1.26E-02	0.003	1.139	2.55E-01	-0.002	0.009	5.53E-01
	L_superiorfrontal	0.003	1.004	3.15E-01	-0.003	0.009	4.75E-01	0.004	1.216	2.24E-01	-0.003	0.011	5.48E-01
	L_superiorparietal	0.010	3.407	6.60E-04	0.004	0.016	4.70E-03	0.002	0.608	5.43E-01	-0.004	0.008	7.17E-01
	L_superiortemporal	-0.005	-1.374	1.69E-01	-0.012	0.002	3.07E-01	0.001	0.378	7.06E-01	-0.006	0.009	8.52E-01
	L_supramarginal	0.003	1.097	2.73E-01	-0.003	0.009	4.32E-01	0.005	1.373	1.70E-01	-0.002	0.011	5.05E-01
	L_transversetemporal	-0.008	-1.550	1.21E-01	-0.018	0.002	2.49E-01	-0.012	-2.107	3.51E-02	-0.023	-0.001	3.39E-01
	R_bankssts	0.002	0.428	6.69E-01	-0.006	0.009	7.78E-01	0.004	0.980	3.27E-01	-0.004	0.013	6.00E-01
	R_caudalanteriorcingulate	0.014	1.834	6.67E-02	-0.001	0.028	1.59E-01	0.015	1.905	5.69E-02	0.000	0.031	3.64E-01
	R_caudalmiddlefrontal	0.007	2.080	3.76E-02	0.000	0.013	1.09E-01	0.008	2.121	3.39E-02	0.001	0.015	3.39E-01
	R_cuneus	0.016	5.397	6.94E-08	0.010	0.022	4.95E-06	0.009	2.706	6.83E-03	0.002	0.015	2.35E-01
	R_entorhinal	0.002	0.285	7.75E-01	-0.012	0.016	8.60E-01	0.014	1.814	6.97E-02	-0.001	0.029	3.83E-01
	R_frontalpole	0.021	4.230	2.36E-05	0.011	0.031	4.21E-04	0.012	2.154	3.13E-02	0.001	0.023	3.39E-01
	R_fusiform	-0.002	-0.587	5.57E-01	-0.008	0.004	6.85E-01	0.003	1.005	3.15E-01	-0.003	0.010	6.00E-01
	R_inferiorparietal	0.006	2.222	2.63E-02	0.001	0.012	8.66E-02	0.004	1.175	2.40E-01	-0.002	0.010	5.48E-01
	R_inferiortemporal	0.003	0.890	3.73E-01	-0.003	0.008	5.19E-01	0.004	1.209	2.27E-01	-0.002	0.010	5.48E-01
	R_insula	0.002	0.411	6.81E-01	-0.006	0.009	7.88E-01	0.011	2.521	1.17E-02	0.002	0.019	2.50E-01
	R_isthmuscingulate	0.002	0.437	6.62E-01	-0.006	0.010	7.74E-01	0.004	0.981	3.27E-01	-0.004	0.013	6.00E-01
	R_lateraloccipital	0.017	6.048	1.52E-09	0.011	0.022	3.25E-07	0.007	2.438	1.48E-02	0.001	0.013	2.88E-01
	R_lateralorbitofrontal	0.000	0.118	9.06E-01	-0.006	0.007	9.40E-01	0.005	1.302	1.93E-01	-0.002	0.011	5.31E-01
	R_lingual	0.015	4.793	1.67E-06	0.009	0.021	4.47E-05	0.010	2.861	4.24E-03	0.003	0.016	2.27E-01
	R_medialorbitofrontal	0.003	0.946	3.44E-01	-0.003	0.010	4.94E-01	0.007	1.802	7.16E-02	-0.001	0.014	3.83E-01
	R_middletemporal	0.009	2.859	4.26E-03	0.003	0.016	2.12E-02	0.012	3.453	5.56E-04	0.005	0.019	1.08E-01
	R_paracentral	-0.003	-0.682	4.95E-01	-0.011	0.005	6.41E-01	0.003	0.641	5.21E-01	-0.006	0.012	6.98E-01
	R_parahippocampal	0.002	0.386	7.00E-01	-0.008	0.012	7.96E-01	0.009	1.613	1.07E-01	-0.002	0.021	4.35E-01
	R_parsopercularis	0.005	1.430	1.53E-01	-0.002	0.011	2.89E-01	0.006	1.761	7.82E-02	-0.001	0.013	3.91E-01
	R_parsorbitalis	0.009	2.414	1.58E-02	0.002	0.017	5.64E-02	0.008	1.997	4.59E-02	0.000	0.017	3.39E-01
	R_parstriangularis	0.006	2.069	3.86E-02	0.000	0.012	1.10E-01	0.004	1.234	2.17E-01	-0.002	0.011	5.48E-01
	R_pericalcarine	0.014	4.877	1.10E-06	0.009	0.020	4.47E-05	0.006	1.943	5.21E-02	0.000	0.013	3.59E-01
	R_postcentral	0.012	3.839	1.25E-04	0.006	0.019	1.49E-03	0.006	1.584	1.13E-01	-0.001	0.013	4.35E-01
	R_posteriorcingulate	0.003	0.758	4.48E-01	-0.005	0.011	5.92E-01	0.006	1.281	2.00E-01	-0.003	0.014	5.43E-01
	R_precentral	0.006	1.482	1.38E-01	-0.002	0.013	2.74E-01	0.008	1.795	7.28E-02	-0.001	0.016	3.83E-01
	R_precuneus	0.007	2.582	9.83E-03	0.002	0.013	3.97E-02	0.006	2.037	4.17E-02	0.000	0.013	3.39E-01
	R_rostralanteriorcingulate	0.006	1.165	2.44E-01	-0.004	0.015	4.05E-01	0.008	1.564	1.18E-01	-0.002	0.019	4.35E-01

	R_rostralmiddlefrontal	0.012	4.543	5.63E-06	0.007	0.017	1.34E-04	0.007	2.566	1.03E-02	0.002	0.013	2.45E-01
	R_superiorfrontal	0.007	2.195	2.82E-02	0.001	0.013	9.14E-02	0.007	2.209	2.72E-02	0.001	0.014	3.39E-01
	R_superiorparietal	0.014	4.926	8.54E-07	0.009	0.020	4.47E-05	0.005	1.686	9.18E-02	-0.001	0.012	4.18E-01
	R_superiortemporal	-0.002	-0.546	5.85E-01	-0.009	0.005	7.05E-01	0.006	1.665	9.59E-02	-0.001	0.014	4.19E-01
	R_supramarginal	0.006	1.917	5.53E-02	0.000	0.012	1.39E-01	0.007	2.023	4.31E-02	0.000	0.014	3.39E-01
	R_transversetemporal	-0.005	-0.980	3.27E-01	-0.016	0.005	4.79E-01	-0.004	-0.677	4.98E-01	-0.016	0.008	6.84E-01
**Cortical Volume**	L_bankssts	-30.466	-3.612	3.05E-04	-46.997	-13.934	2.97E-03	-18.096	-1.957	5.04E-02	-36.224	0.032	3.59E-01
	L_caudalanteriorcingulate	-5.221	-0.582	5.60E-01	-22.792	12.351	6.85E-01	-9.761	-0.992	3.21E-01	-29.054	9.532	6.00E-01
	L_caudalmiddlefrontal	-19.898	-1.227	2.20E-01	-51.694	11.898	3.74E-01	2.396	0.135	8.93E-01	-32.482	37.275	9.46E-01
	L_cuneus	1.121	0.116	9.08E-01	-17.819	20.061	9.40E-01	8.976	0.846	3.97E-01	-11.809	29.761	6.40E-01
	L_entorhinal	-8.470	-1.411	1.58E-01	-20.234	3.293	2.94E-01	-0.961	-0.146	8.84E-01	-13.869	11.946	9.46E-01
	L_frontalpole	1.214	0.657	5.11E-01	-2.405	4.832	6.47E-01	0.250	0.124	9.02E-01	-3.718	4.218	9.51E-01
	L_fusiform	-48.796	-2.809	4.99E-03	-82.849	-14.743	2.37E-02	-23.350	-1.226	2.20E-01	-60.691	13.991	5.48E-01
	L_inferiorparietal	-100.872	-3.529	4.19E-04	-156.894	-44.850	3.53E-03	-41.239	-1.317	1.88E-01	-102.607	20.129	5.31E-01
	L_inferiortemporal	-82.951	-3.763	1.69E-04	-126.154	-39.749	1.81E-03	-26.858	-1.112	2.66E-01	-74.188	20.472	5.53E-01
	L_insula	-1.490	-0.119	9.05E-01	-26.010	23.030	9.40E-01	2.851	0.208	8.35E-01	-24.036	29.738	9.30E-01
	L_isthmuscingulate	13.865	2.123	3.38E-02	1.063	26.667	1.02E-01	7.012	0.978	3.28E-01	-7.034	21.057	6.00E-01
	L_lateraloccipital	63.377	2.474	1.34E-02	13.162	113.592	5.03E-02	65.332	2.324	2.02E-02	10.223	120.442	3.39E-01
	L_lateralorbitofrontal	-13.965	-1.148	2.51E-01	-37.803	9.873	4.07E-01	2.159	0.162	8.71E-01	-23.963	28.282	9.42E-01
	L_lingual	22.479	1.219	2.23E-01	-13.671	58.629	3.75E-01	35.354	1.746	8.08E-02	-4.323	75.030	3.93E-01
	L_medialorbitofrontal	-26.475	-2.563	1.04E-02	-46.722	-6.228	4.12E-02	-13.441	-1.187	2.35E-01	-35.637	8.756	5.48E-01
	L_middletemporal	-67.455	-3.389	7.05E-04	-106.468	-28.443	4.86E-03	-16.314	-0.749	4.54E-01	-59.029	26.401	6.59E-01
	L_paracentral	-2.120	-0.268	7.89E-01	-17.613	13.373	8.70E-01	4.403	0.508	6.12E-01	-12.590	21.396	7.70E-01
	L_parahippocampal	-5.218	-1.022	3.07E-01	-15.228	4.792	4.66E-01	2.497	0.446	6.56E-01	-8.482	13.476	8.11E-01
	L_parsopercularis	-9.091	-0.736	4.62E-01	-33.294	15.113	6.06E-01	-0.921	-0.068	9.46E-01	-27.470	25.627	9.67E-01
	L_parsorbitalis	1.202	0.261	7.94E-01	-7.808	10.212	8.71E-01	8.422	1.671	9.47E-02	-1.453	18.297	4.19E-01
	L_parstriangularis	-5.071	-0.544	5.87E-01	-23.346	13.204	7.05E-01	2.540	0.248	8.04E-01	-17.502	22.582	9.20E-01
	L_pericalcarine	3.653	0.379	7.05E-01	-15.260	22.566	7.98E-01	11.020	1.040	2.98E-01	-9.739	31.779	5.91E-01
	L_postcentral	-34.948	-1.812	7.00E-02	-72.747	2.852	1.65E-01	-19.270	-0.911	3.62E-01	-60.727	22.186	6.30E-01
	L_posteriorcingulate	-13.244	-1.612	1.07E-01	-29.347	2.858	2.27E-01	-11.127	-1.234	2.17E-01	-28.805	6.550	5.48E-01
	L_precentral	-32.899	-1.400	1.62E-01	-78.966	13.168	2.98E-01	-6.543	-0.254	7.99E-01	-56.995	43.910	9.20E-01
	L_precuneus	-22.745	-1.156	2.48E-01	-61.314	15.823	4.05E-01	-17.697	-0.820	4.13E-01	-60.022	24.627	6.49E-01
	L_rostralanteriorcingulate	12.011	1.505	1.32E-01	-3.634	27.655	2.65E-01	6.017	0.687	4.92E-01	-11.152	23.187	6.84E-01
	L_rostralmiddlefrontal	-41.551	-1.426	1.54E-01	-98.649	15.547	2.89E-01	11.579	0.363	7.17E-01	-50.990	74.149	8.52E-01
	L_superiorfrontal	-39.756	-1.091	2.75E-01	-111.197	31.685	4.33E-01	-7.662	-0.192	8.48E-01	-85.981	70.657	9.31E-01
	L_superiorparietal	-44.310	-1.589	1.12E-01	-98.970	10.350	2.35E-01	-15.365	-0.503	6.15E-01	-75.272	44.542	7.70E-01
	L_superiortemporal	-80.843	-3.524	4.27E-04	-125.803	-35.882	3.53E-03	-29.878	-1.189	2.35E-01	-79.138	19.381	5.48E-01
	L_supramarginal	-74.383	-2.647	8.13E-03	-129.460	-19.307	3.41E-02	-23.763	-0.772	4.40E-01	-84.105	36.579	6.59E-01
	L_transversetemporal	-5.945	-1.730	8.36E-02	-12.680	0.789	1.90E-01	-10.123	-2.686	7.25E-03	-17.511	-2.735	2.35E-01
	R_bankssts	-17.684	-2.458	1.40E-02	-31.784	-3.583	5.16E-02	-6.870	-0.871	3.84E-01	-22.332	8.593	6.40E-01
	R_caudalanteriorcingulate	6.023	0.678	4.98E-01	-11.381	23.428	6.41E-01	8.835	0.906	3.65E-01	-10.269	27.940	6.30E-01
	R_caudalmiddlefrontal	-35.939	-2.183	2.91E-02	-68.205	-3.674	9.28E-02	-17.542	-0.971	3.32E-01	-52.953	17.868	6.01E-01
	R_cuneus	10.972	1.159	2.47E-01	-7.584	29.527	4.05E-01	18.778	1.807	7.08E-02	-1.589	39.145	3.83E-01
	R_entorhinal	0.580	0.113	9.10E-01	-9.462	10.622	9.40E-01	6.694	1.191	2.34E-01	-4.320	17.709	5.48E-01
	R_frontalpole	2.988	1.341	1.80E-01	-1.379	7.354	3.17E-01	2.207	0.903	3.66E-01	-2.582	6.995	6.30E-01
	R_fusiform	-36.483	-2.146	3.19E-02	-69.811	-3.155	1.01E-01	-18.622	-0.999	3.18E-01	-55.170	17.925	6.00E-01

	R_inferiorparietal	-86.492	-2.684	7.29E-03	-149.653	-23.331	3.25E-02	-12.828	-0.363	7.16E-01	-82.000	56.344	8.52E-01
	R_inferiortemporal	-70.666	-3.416	6.38E-04	-111.212	-30.121	4.70E-03	-39.927	-1.760	7.85E-02	-84.401	4.547	3.91E-01
	R_insula	17.159	1.288	1.98E-01	-8.953	43.271	3.41E-01	23.865	1.633	1.03E-01	-4.781	52.511	4.35E-01
	R_isthmuscingulate	5.544	0.902	3.67E-01	-6.503	17.590	5.17E-01	4.215	0.625	5.32E-01	-9.008	17.439	7.07E-01
	R_lateraloccipital	35.802	1.321	1.87E-01	-17.314	88.918	3.24E-01	19.428	0.653	5.13E-01	-38.845	77.701	6.95E-01
	R_lateralorbitofrontal	-21.741	-1.711	8.72E-02	-46.649	3.168	1.94E-01	0.727	0.052	9.58E-01	-26.566	28.020	9.67E-01
	R_lingual	37.770	1.924	5.44E-02	-0.705	76.244	1.39E-01	56.844	2.639	8.33E-03	14.627	99.060	2.35E-01
	R_medialorbitofrontal	-7.269	-0.800	4.24E-01	-25.085	10.547	5.67E-01	3.959	0.397	6.91E-01	-15.576	23.494	8.48E-01
	R_middletemporal	-39.026	-1.966	4.94E-02	-77.936	-0.116	1.30E-01	0.369	0.017	9.86E-01	-42.258	42.996	9.86E-01
	R_paracentral	9.566	1.032	3.02E-01	-8.606	27.737	4.65E-01	14.578	1.433	1.52E-01	-5.356	34.512	4.85E-01
	R_parahippocampal	1.704	0.393	6.94E-01	-6.786	10.193	7.96E-01	10.158	2.138	3.25E-02	0.848	19.467	3.39E-01
	R_parsopercularis	8.556	0.860	3.90E-01	-10.955	28.067	5.37E-01	16.862	1.544	1.23E-01	-4.538	38.263	4.35E-01
	R_parsorbitalis	-4.952	-0.897	3.69E-01	-15.767	5.863	5.17E-01	3.626	0.600	5.49E-01	-8.220	15.473	7.20E-01
	R_parstriangularis	7.489	0.665	5.06E-01	-14.575	29.553	6.44E-01	13.172	1.067	2.86E-01	-11.028	37.371	5.89E-01
	R_pericalcarine	12.734	1.248	2.12E-01	-7.272	32.740	3.63E-01	23.359	2.085	3.71E-02	1.404	45.314	3.39E-01
	R_postcentral	-29.318	-1.510	1.31E-01	-67.362	8.725	2.64E-01	-17.281	-0.811	4.17E-01	-59.028	24.466	6.52E-01
	R_posteriorcingulate	-16.410	-1.994	4.62E-02	-32.541	-0.280	1.25E-01	-9.428	-1.044	2.97E-01	-27.128	8.272	5.91E-01
	R_precentral	-9.348	-0.388	6.98E-01	-56.569	37.873	7.96E-01	26.614	1.008	3.13E-01	-25.119	78.347	6.00E-01
	R_precuneus	-12.696	-0.640	5.22E-01	-51.575	26.183	6.50E-01	4.050	0.186	8.52E-01	-38.612	46.712	9.31E-01
	R_rostralanteriorcingulate	8.199	1.215	2.24E-01	-5.022	21.421	3.75E-01	10.533	1.422	1.55E-01	-3.983	25.049	4.88E-01
	R_rostralmiddlefrontal	-62.706	-2.011	4.44E-02	-123.829	-1.583	1.23E-01	-2.265	-0.066	9.47E-01	-69.238	64.708	9.67E-01
	R_superiorfrontal	-20.778	-0.577	5.64E-01	-91.373	49.817	6.86E-01	-2.358	-0.060	9.52E-01	-79.715	74.999	9.67E-01
	R_superiorparietal	27.440	1.047	2.95E-01	-23.944	78.823	4.58E-01	44.210	1.538	1.24E-01	-12.136	100.555	4.35E-01
	R_superiortemporal	-55.846	-2.759	5.80E-03	-95.512	-16.180	2.64E-02	-16.321	-0.736	4.62E-01	-59.783	27.141	6.59E-01
	R_supramarginal	-48.167	-1.980	4.77E-02	-95.845	-0.490	1.28E-01	-25.504	-0.956	3.39E-01	-77.794	26.787	6.05E-01
	R_transversetemporal	-7.861	-3.241	1.19E-03	-12.614	-3.107	7.75E-03	-8.749	-3.289	1.01E-03	-13.963	-3.535	1.08E-01
**Subcortical Volume**	Laccumb	0.121	0.079	9.37E-01	-2.894	3.136	9.55E-01	-0.343	-0.204	8.39E-01	-3.648	2.961	9.30E-01
	Lamyg	11.535	3.099	1.95E-03	4.240	18.829	1.13E-02	8.716	2.135	3.28E-02	0.716	16.717	3.39E-01
	Lcaud	25.525	3.192	1.42E-03	9.851	41.200	8.93E-03	17.669	2.016	4.39E-02	0.489	34.849	3.39E-01
	Lhippo	9.427	1.394	1.63E-01	-3.826	22.680	2.99E-01	8.442	1.138	2.55E-01	-6.094	22.978	5.53E-01
	LLatVent	321.765	2.233	2.56E-02	39.347	604.184	8.55E-02	136.682	0.865	3.87E-01	-172.966	446.329	6.40E-01
	Lpal	0.148	0.039	9.69E-01	-7.255	7.551	9.78E-01	8.262	1.997	4.59E-02	0.152	16.371	3.39E-01
	Lput	31.637	3.413	6.45E-04	13.470	49.804	4.70E-03	14.011	1.380	1.68E-01	-5.885	33.907	5.05E-01
	Lthal	-5.140	-0.486	6.27E-01	-25.861	15.580	7.41E-01	-7.421	-0.640	5.22E-01	-30.143	15.301	6.98E-01
	Raccumb	-0.128	-0.087	9.30E-01	-3.001	2.745	9.53E-01	-0.505	-0.315	7.53E-01	-3.653	2.643	8.86E-01
	Ramyg	8.328	2.250	2.45E-02	1.074	15.582	8.44E-02	4.180	1.030	3.03E-01	-3.777	12.137	5.95E-01
	Rcaud	31.719	3.888	1.02E-04	15.729	47.709	1.45E-03	18.907	2.115	3.45E-02	1.383	36.430	3.39E-01
	Rhippo	4.575	0.646	5.18E-01	-9.297	18.447	6.48E-01	1.439	0.185	8.53E-01	-13.784	16.662	9.31E-01
	RLatVent	270.409	2.135	3.28E-02	22.198	518.620	1.02E-01	107.201	0.772	4.40E-01	-164.880	379.281	6.59E-01
	Rpal	-0.686	-0.184	8.54E-01	-7.998	6.625	9.18E-01	5.373	1.313	1.89E-01	-2.646	13.392	5.31E-01
	Rput	23.462	2.491	1.27E-02	5.004	41.921	4.87E-02	10.747	1.041	2.98E-01	-9.483	30.977	5.91E-01
	Rthal	20.505	2.091	3.66E-02	1.283	39.726	1.09E-01	5.154	0.479	6.32E-01	-15.916	26.225	7.86E-01

Supplementary Table 6: The association between tinnitus without hearing loss (tinnitus only), mean thickness and volume in the UK Biobank. Values highlighted in red are the significant values at respective P-value and corrected P-value at 0.005. ICV: intracranial volume; CVD: cardiovascular disease; BMI: body mass index; TDI, Townsend deprivation index; ROI: region of interest; IDP: imaging derived phenotypes; CI: confidence interval; FDR: false discovery rate.

Cases (854) vs. Controls (854)	IDP	Model 1: IDP ~ Tinnitus_only + Age + Sex + ICV						Model 2: IDP ~ Tinnitus_only + Age + Sex + ICV + Smoking + Alcohol + Diabetes + CVD + BMI + TDI					
		Estimate	T-value	P-value	Lower-CI	Upper-CI	FDR	Estimate	T-value	P-value	Lower-CI	Upper-CI	FDR
Cortical Surface Area	L_bankssts	-6.528	-1.011	3.12E-01	-19.180	6.124	5.12E-01	-3.805	-0.422	6.73E-01	-21.459	13.849	9.68E-01
	L_caudalanteriorcingulate	6.040	1.009	3.13E-01	-5.694	17.773	5.12E-01	8.607	1.029	3.03E-01	-7.782	24.997	9.06E-01
	L_caudalmiddlefrontal	-46.906	-3.548	3.98E-04	-72.816	-20.997	7.62E-03	-24.536	-1.332	1.83E-01	-60.652	11.580	8.90E-01
	L_cuneus	-17.639	-1.788	7.40E-02	-36.977	1.699	2.17E-01	-1.655	-0.120	9.04E-01	-28.627	25.317	9.82E-01
	L_entorhinal	-7.989	-1.920	5.51E-02	-16.146	0.168	1.77E-01	-4.323	-0.743	4.57E-01	-15.718	7.073	9.06E-01
	L_frontalpole	-2.629	-2.121	3.41E-02	-5.057	-0.200	1.27E-01	-1.314	-0.760	4.47E-01	-4.702	2.074	9.06E-01
	L_fusiform	-13.486	-1.020	3.08E-01	-39.389	12.417	5.12E-01	9.592	0.522	6.01E-01	-26.390	45.574	9.68E-01
	L_inferiorparietal	-63.991	-2.634	8.51E-03	-111.606	-16.376	5.41E-02	-15.374	-0.454	6.50E-01	-81.731	50.983	9.68E-01
	L_inferiortemporal	-17.428	-1.022	3.07E-01	-50.855	15.999	5.12E-01	42.430	1.791	7.35E-02	-4.010	88.870	8.02E-01
	L_insula	-1.556	-0.164	8.69E-01	-20.095	16.983	9.05E-01	2.230	0.169	8.66E-01	-23.659	28.119	9.82E-01
	L_isthmuscingulate	6.771	1.065	2.87E-01	-5.695	19.238	5.08E-01	13.844	1.559	1.19E-01	-3.558	31.245	8.02E-01
	L_lateraloccipital	-19.317	-0.786	4.32E-01	-67.501	28.868	6.51E-01	68.790	2.014	4.42E-02	1.830	135.750	8.02E-01
	L_lateralorbitofrontal	-11.063	-1.225	2.21E-01	-28.767	6.641	4.38E-01	-18.375	-1.460	1.44E-01	-43.038	6.287	8.13E-01
	L_lingual	-15.058	-0.884	3.77E-01	-48.460	18.343	5.85E-01	8.545	0.360	7.19E-01	-37.980	55.071	9.82E-01
	L_medialorbitofrontal	-14.071	-1.654	9.84E-02	-30.748	2.606	2.57E-01	-15.106	-1.272	2.04E-01	-38.388	8.177	8.90E-01
	L_middletemporal	4.947	0.348	7.28E-01	-22.918	32.812	8.51E-01	39.999	2.023	4.33E-02	1.241	78.758	8.02E-01
	L_paracentral	-6.984	-1.080	2.80E-01	-19.664	5.695	5.08E-01	-7.430	-0.823	4.11E-01	-25.131	10.271	9.06E-01
	L_parahippocampal	0.468	0.145	8.84E-01	-5.836	6.771	9.08E-01	3.398	0.756	4.50E-01	-5.409	12.205	9.06E-01
	L_parsopercularis	-12.008	-1.176	2.40E-01	-32.026	8.010	4.65E-01	-9.484	-0.665	5.06E-01	-37.451	18.483	9.10E-01
	L_parsorbitalis	-0.984	-0.292	7.70E-01	-7.588	5.620	8.72E-01	7.333	1.562	1.18E-01	-1.868	16.534	8.02E-01
	L_parstriangularis	-3.576	-0.441	6.59E-01	-19.473	12.321	8.35E-01	2.578	0.228	8.20E-01	-19.628	24.784	9.82E-01
	L_pericalcarine	-17.111	-1.460	1.45E-01	-40.086	5.864	3.58E-01	-6.679	-0.408	6.83E-01	-38.731	25.373	9.68E-01
	L_postcentral	-39.923	-2.467	1.37E-02	-71.638	-8.209	7.34E-02	21.498	0.958	3.38E-01	-22.485	65.481	9.06E-01
	L_posteriorcingulate	-0.503	-0.072	9.43E-01	-14.228	13.221	9.56E-01	8.455	0.866	3.87E-01	-10.685	27.595	9.06E-01
	L_precentral	-18.760	-1.054	2.92E-01	-53.630	16.110	5.08E-01	18.449	0.746	4.56E-01	-30.032	66.930	9.06E-01
	L_precuneus	-24.597	-1.398	1.62E-01	-59.083	9.888	3.78E-01	-29.562	-1.203	2.29E-01	-77.712	18.587	9.06E-01
	L_rostralanteriorcingulate	1.397	0.213	8.31E-01	-11.428	14.221	8.88E-01	-2.393	-0.262	7.93E-01	-20.275	15.488	9.82E-01
	L_rostralmiddlefrontal	-76.406	-2.965	3.07E-03	-126.908	-25.905	2.73E-02	-23.286	-0.648	5.17E-01	-93.725	47.153	9.10E-01
	L_superiorfrontal	-37.898	-1.359	1.74E-01	-92.556	16.760	3.93E-01	8.638	0.222	8.24E-01	-67.486	84.763	9.82E-01
	L_superiorparietal	-90.342	-3.519	4.45E-04	-140.665	-40.020	7.62E-03	-68.262	-1.905	5.69E-02	-138.491	1.967	8.02E-01
	L_superiortemporal	-15.217	-1.008	3.13E-01	-44.795	14.362	5.12E-01	-3.674	-0.175	8.61E-01	-44.925	37.577	9.82E-01
	L_supramarginal	-50.636	-2.151	3.16E-02	-96.777	-4.494	1.21E-01	-29.081	-0.885	3.76E-01	-93.472	35.310	9.06E-01
	L_transversetemporal	-0.636	-0.209	8.34E-01	-6.596	5.325	8.88E-01	-1.771	-0.417	6.76E-01	-10.088	6.545	9.68E-01
	R_bankssts	-1.676	-0.325	7.45E-01	-11.785	8.433	8.57E-01	12.737	1.775	7.61E-02	-1.327	26.801	8.02E-01

	R_caudalanteriorcingulate	-4.848	-0.732	4.64E-01	-17.822	8.127	6.76E-01	6.667	0.722	4.70E-01	-11.421	24.754	9.09E-01
	R_caudalmiddlefrontal	-42.074	-3.072	2.16E-03	-68.916	-15.233	2.31E-02	-25.440	-1.332	1.83E-01	-62.864	11.984	8.90E-01
	R_cuneus	-24.890	-2.580	9.97E-03	-43.801	-5.979	5.68E-02	-6.745	-0.501	6.16E-01	-33.120	19.631	9.68E-01
	R_entorhinal	-7.687	-2.229	2.60E-02	-14.448	-0.927	1.07E-01	-7.418	-1.540	1.24E-01	-16.856	2.020	8.02E-01
	R_frontalpole	1.227	0.793	4.28E-01	-1.806	4.259	6.50E-01	3.624	1.681	9.30E-02	-0.602	7.850	8.02E-01
	R_fusiform	-15.661	-1.162	2.45E-01	-42.081	10.760	4.65E-01	-6.637	-0.354	7.23E-01	-43.359	30.086	9.82E-01
	R_inferiorparietal	-33.949	-1.242	2.14E-01	-87.507	19.609	4.37E-01	3.990	0.105	9.17E-01	-70.695	78.676	9.82E-01
	R_inferiortemporal	-30.280	-1.917	5.53E-02	-61.232	0.671	1.77E-01	-5.924	-0.269	7.88E-01	-49.067	37.219	9.82E-01
	R_insula	5.437	0.490	6.24E-01	-16.301	27.175	8.14E-01	-14.140	-0.914	3.61E-01	-44.457	16.177	9.06E-01
	R_isthmuscingulate	6.010	1.090	2.76E-01	-4.793	16.814	5.06E-01	3.508	0.456	6.49E-01	-11.578	18.593	9.68E-01
	R_lateraloccipital	-6.101	-0.240	8.10E-01	-55.853	43.651	8.82E-01	35.759	1.011	3.12E-01	-33.597	105.114	9.06E-01
	R_lateralorbitofrontal	-18.633	-1.633	1.03E-01	-40.993	3.728	2.65E-01	-0.844	-0.053	9.58E-01	-31.993	30.305	9.84E-01
	R_lingual	-11.977	-0.646	5.18E-01	-48.297	24.344	7.25E-01	25.440	0.987	3.24E-01	-25.074	75.955	9.06E-01
	R_medialorbitofrontal	-19.586	-2.614	9.04E-03	-34.274	-4.898	5.41E-02	-12.338	-1.180	2.38E-01	-32.830	8.153	9.06E-01
	R_middletemporal	0.176	0.013	9.90E-01	-26.933	27.285	9.95E-01	33.011	1.713	8.70E-02	-4.768	70.789	8.02E-01
	R_paracentral	1.839	0.243	8.08E-01	-13.007	16.686	8.82E-01	9.136	0.864	3.88E-01	-11.596	29.868	9.06E-01
	R_parahippocampal	1.354	0.446	6.55E-01	-4.588	7.296	8.35E-01	5.227	1.237	2.16E-01	-3.056	13.510	8.98E-01
	R_parsopercularis	-19.505	-2.305	2.13E-02	-36.088	-2.923	9.52E-02	-14.550	-1.232	2.18E-01	-37.698	8.598	8.98E-01
	R_parsorbitalis	-2.264	-0.567	5.71E-01	-10.091	5.563	7.87E-01	10.080	1.812	7.02E-02	-0.823	20.983	8.02E-01
	R_parstriangularis	-3.251	-0.332	7.40E-01	-22.467	15.965	8.56E-01	0.172	0.013	9.90E-01	-26.630	26.973	9.95E-01
	R_pericalcarine	-13.418	-1.088	2.77E-01	-37.593	10.757	5.06E-01	-6.517	-0.378	7.05E-01	-40.266	27.232	9.82E-01
	R_postcentral	-43.274	-2.642	8.32E-03	-75.378	-11.170	5.41E-02	4.431	0.195	8.45E-01	-40.102	48.963	9.82E-01
	R_posteriorcingulate	-7.106	-1.008	3.13E-01	-20.918	6.706	5.12E-01	3.424	0.349	7.27E-01	-15.828	22.676	9.82E-01
	R_precentral	-40.742	-2.315	2.07E-02	-75.237	-6.247	9.52E-02	3.514	0.144	8.86E-01	-44.434	51.462	9.82E-01
	R_precuneus	-27.969	-1.543	1.23E-01	-63.499	7.561	3.13E-01	-23.182	-0.916	3.60E-01	-72.763	26.399	9.06E-01
	R_rostralanteriorcingulate	-0.703	-0.136	8.92E-01	-10.798	9.393	9.08E-01	10.851	1.510	1.31E-01	-3.237	24.938	8.13E-01
	R_rostralmiddlefrontal	-53.801	-1.923	5.46E-02	-108.634	1.033	1.77E-01	-3.716	-0.095	9.24E-01	-80.095	72.664	9.82E-01
	R_superiorfrontal	-19.894	-0.694	4.88E-01	-76.075	36.287	7.01E-01	3.032	0.076	9.40E-01	-75.339	81.402	9.82E-01
	R_superiorparietal	-61.946	-2.641	8.35E-03	-107.924	-15.967	5.41E-02	-29.549	-0.902	3.67E-01	-93.739	34.641	9.06E-01
	R_superiortemporal	-18.644	-1.365	1.72E-01	-45.411	8.124	3.92E-01	8.827	0.463	6.43E-01	-28.521	46.174	9.68E-01
	R_supramarginal	-38.859	-1.971	4.89E-02	-77.501	-0.216	1.70E-01	3.901	0.142	8.87E-01	-49.987	57.789	9.82E-01
	R_transversetemporal	0.790	0.396	6.92E-01	-3.119	4.699	8.51E-01	-0.190	-0.068	9.46E-01	-5.651	5.270	9.82E-01
**Cortical Mean Thickness**	L_bankssts	0.016	1.824	6.84E-02	-0.001	0.033	2.12E-01	0.006	0.511	6.10E-01	-0.018	0.030	9.68E-01
	L_caudalanteriorcingulate	-0.011	-0.500	6.17E-01	-0.053	0.032	8.10E-01	0.017	0.549	5.83E-01	-0.043	0.076	9.68E-01
	L_caudalmiddlefrontal	0.013	1.755	7.94E-02	-0.002	0.028	2.21E-01	0.002	0.194	8.46E-01	-0.018	0.023	9.82E-01
	L_cuneus	0.019	2.611	9.10E-03	0.005	0.034	5.41E-02	-0.014	-1.404	1.60E-01	-0.034	0.006	8.38E-01
	L_entorhinal	-0.022	-1.492	1.36E-01	-0.052	0.007	3.42E-01	-0.023	-1.119	2.63E-01	-0.064	0.018	9.06E-01
	L_frontalpole	0.024	1.998	4.59E-02	0.000	0.047	1.64E-01	0.010	0.617	5.38E-01	-0.022	0.042	9.28E-01
	L_fusiform	-0.004	-0.547	5.85E-01	-0.016	0.009	7.92E-01	-0.006	-0.651	5.15E-01	-0.024	0.012	9.10E-01
	L_inferiorparietal	0.013	2.187	2.89E-02	0.001	0.025	1.14E-01	0.006	0.752	4.52E-01	-0.010	0.023	9.06E-01
	L_inferiortemporal	-0.012	-1.775	7.62E-02	-0.026	0.001	2.18E-01	-0.008	-0.800	4.24E-01	-0.027	0.011	9.06E-01
	L_insula	-0.011	-1.290	1.97E-01	-0.029	0.006	4.22E-01	-0.013	-1.066	2.87E-01	-0.037	0.011	9.06E-01
	L_isthmuscingulate	-0.012	-1.408	1.59E-01	-0.030	0.005	3.78E-01	-0.018	-1.480	1.39E-01	-0.042	0.006	8.13E-01
	L_lateraloccipital	0.026	4.236	2.40E-05	0.014	0.038	1.28E-03	0.002	0.290	7.72E-01	-0.014	0.019	9.82E-01
	L_lateralorbitofrontal	0.001	0.140	8.89E-01	-0.013	0.014	9.08E-01	-0.003	-0.334	7.38E-01	-0.022	0.016	9.82E-01
	L_lingual	0.015	2.118	3.44E-02	0.001	0.030	1.27E-01	0.003	0.252	8.01E-01	-0.017	0.022	9.82E-01

	L_medialorbitofrontal	0.005	0.647	5.18E-01	-0.010	0.021	7.25E-01	0.003	0.239	8.11E-01	-0.019	0.024	9.82E-01
	L_middletemporal	-0.002	-0.310	7.56E-01	-0.018	0.013	8.66E-01	-0.005	-0.479	6.32E-01	-0.027	0.016	9.68E-01
	L_paracentral	0.005	0.516	6.06E-01	-0.014	0.024	8.05E-01	0.001	0.040	9.68E-01	-0.026	0.027	9.84E-01
	L_parahippocampal	-0.044	-2.993	2.80E-03	-0.072	-0.015	2.73E-02	-0.046	-2.268	2.34E-02	-0.086	-0.006	8.02E-01
	L_parsopercularis	0.009	1.245	2.13E-01	-0.005	0.024	4.37E-01	0.007	0.675	5.00E-01	-0.013	0.027	9.10E-01
	L_parsorbitalis	0.015	1.706	8.82E-02	-0.002	0.033	2.34E-01	0.003	0.224	8.23E-01	-0.022	0.027	9.82E-01
	L_parstriangularis	0.018	2.423	1.55E-02	0.003	0.033	8.09E-02	0.007	0.694	4.88E-01	-0.013	0.028	9.10E-01
	L_pericalcarine	0.009	1.308	1.91E-01	-0.005	0.023	4.16E-01	-0.014	-1.468	1.42E-01	-0.033	0.005	8.13E-01
	L_postcentral	0.019	2.741	6.19E-03	0.006	0.033	4.58E-02	0.003	0.296	7.67E-01	-0.016	0.022	9.82E-01
	L_posteriorcingulate	-0.010	-1.135	2.57E-01	-0.028	0.008	4.77E-01	-0.015	-1.213	2.25E-01	-0.040	0.010	9.06E-01
	L_precentral	0.004	0.505	6.14E-01	-0.013	0.021	8.10E-01	-0.007	-0.549	5.83E-01	-0.030	0.017	9.68E-01
	L_precuneus	0.024	3.520	4.43E-04	0.010	0.037	7.62E-03	0.016	1.714	8.67E-02	-0.002	0.034	8.02E-01
	L_rostralanteriorcingulate	-0.012	-1.053	2.92E-01	-0.033	0.010	5.08E-01	-0.002	-0.126	9.00E-01	-0.032	0.028	9.82E-01
	L_rostralmiddlefrontal	0.025	3.940	8.47E-05	0.013	0.037	2.59E-03	0.010	1.125	2.61E-01	-0.007	0.027	9.06E-01
	L_superiorfrontal	0.013	1.774	7.63E-02	-0.001	0.027	2.18E-01	0.001	0.129	8.98E-01	-0.019	0.021	9.82E-01
	L_superiorparietal	0.027	4.115	4.07E-05	0.014	0.040	1.74E-03	0.010	1.122	2.62E-01	-0.008	0.028	9.06E-01
	L_superiortemporal	-0.018	-2.208	2.74E-02	-0.035	-0.002	1.10E-01	-0.011	-0.920	3.57E-01	-0.033	0.012	9.06E-01
	L_supramarginal	0.010	1.433	1.52E-01	-0.004	0.023	3.70E-01	0.004	0.452	6.51E-01	-0.014	0.023	9.68E-01
	L_transversetemporal	-0.021	-1.763	7.81E-02	-0.045	0.002	2.20E-01	-0.014	-0.851	3.95E-01	-0.047	0.019	9.06E-01
	R_bankssts	0.016	1.794	7.30E-02	-0.001	0.034	2.17E-01	0.023	1.873	6.12E-02	-0.001	0.048	8.02E-01
	R_caudalanteriorcingulate	0.003	0.181	8.57E-01	-0.030	0.036	8.99E-01	0.019	0.825	4.10E-01	-0.027	0.066	9.06E-01
	R_caudalmiddlefrontal	0.016	2.231	2.58E-02	0.002	0.031	1.07E-01	0.009	0.891	3.73E-01	-0.011	0.029	9.06E-01
	R_cuneus	0.022	3.142	1.70E-03	0.008	0.035	1.92E-02	-0.005	-0.505	6.13E-01	-0.024	0.014	9.68E-01
	R_entorhinal	-0.038	-2.318	2.06E-02	-0.069	-0.006	9.52E-02	-0.053	-2.332	1.98E-02	-0.097	-0.008	8.02E-01
	R_frontalpole	0.029	2.576	1.01E-02	0.007	0.051	5.68E-02	0.003	0.182	8.55E-01	-0.028	0.034	9.82E-01
	R_fusiform	-0.003	-0.365	7.15E-01	-0.016	0.011	8.51E-01	0.001	0.147	8.83E-01	-0.018	0.020	9.82E-01
	R_inferiorparietal	0.020	3.188	1.46E-03	0.008	0.033	1.73E-02	0.006	0.682	4.95E-01	-0.011	0.024	9.10E-01
	R_inferiortemporal	0.002	0.223	8.24E-01	-0.012	0.015	8.86E-01	0.001	0.127	8.99E-01	-0.018	0.020	9.82E-01
	R_insula	-0.004	-0.457	6.48E-01	-0.022	0.013	8.30E-01	0.000	0.016	9.88E-01	-0.024	0.024	9.95E-01
	R_isthmuscingulate	-0.009	-0.967	3.34E-01	-0.027	0.009	5.37E-01	0.001	0.088	9.30E-01	-0.024	0.027	9.82E-01
	R_lateraloccipital	0.026	4.033	5.74E-05	0.014	0.039	2.05E-03	0.000	0.047	9.62E-01	-0.017	0.018	9.84E-01
	R_lateralorbitofrontal	0.005	0.712	4.77E-01	-0.009	0.020	6.89E-01	-0.002	-0.176	8.60E-01	-0.022	0.018	9.82E-01
	R_lingual	0.021	2.942	3.31E-03	0.007	0.035	2.83E-02	0.009	0.938	3.48E-01	-0.010	0.029	9.06E-01
	R_medialorbitofrontal	0.014	1.797	7.24E-02	-0.001	0.029	2.17E-01	0.009	0.815	4.15E-01	-0.012	0.030	9.06E-01
	R_middletemporal	0.014	1.968	4.92E-02	0.000	0.029	1.70E-01	0.012	1.163	2.45E-01	-0.008	0.032	9.06E-01
	R_paracentral	0.002	0.194	8.46E-01	-0.016	0.020	8.97E-01	-0.002	-0.150	8.81E-01	-0.027	0.023	9.82E-01
	R_parahippocampal	-0.024	-1.955	5.07E-02	-0.049	0.000	1.72E-01	-0.009	-0.509	6.11E-01	-0.043	0.025	9.68E-01
	R_parsopercularis	0.018	2.519	1.19E-02	0.004	0.032	6.51E-02	0.020	1.977	4.82E-02	0.000	0.039	8.02E-01
	R_parsorbitalis	0.026	2.973	2.99E-03	0.009	0.043	2.73E-02	0.001	0.087	9.31E-01	-0.022	0.025	9.82E-01
	R_parstriangularis	0.022	3.199	1.40E-03	0.009	0.036	1.73E-02	0.016	1.677	9.38E-02	-0.003	0.036	8.02E-01
	R_pericalcarine	0.022	3.259	1.14E-03	0.009	0.035	1.63E-02	0.001	0.069	9.45E-01	-0.018	0.019	9.82E-01
	R_postcentral	0.028	3.799	1.50E-04	0.014	0.042	4.02E-03	0.020	1.993	4.64E-02	0.000	0.041	8.02E-01
	R_posteriorcingulate	0.008	0.854	3.93E-01	-0.010	0.026	6.05E-01	0.009	0.685	4.93E-01	-0.016	0.034	9.10E-01
	R_precentral	0.011	1.324	1.86E-01	-0.005	0.028	4.10E-01	0.006	0.489	6.25E-01	-0.018	0.029	9.68E-01
	R_precuneus	0.024	3.508	4.63E-04	0.010	0.037	7.62E-03	0.016	1.721	8.54E-02	-0.002	0.034	8.02E-01
	R_rostralanteriorcingulate	0.006	0.557	5.77E-01	-0.015	0.028	7.87E-01	0.000	-0.003	9.98E-01	-0.030	0.030	9.98E-01

	R_rostralmiddlefrontal	0.029	4.734	2.38E-06	0.017	0.040	2.55E-04	0.015	1.830	6.74E-02	-0.001	0.032	8.02E-01
	R_superiorfrontal	0.022	3.052	2.31E-03	0.008	0.036	2.35E-02	0.010	0.977	3.29E-01	-0.010	0.029	9.06E-01
	R_superiorparietal	0.032	4.802	1.71E-06	0.019	0.045	2.55E-04	0.012	1.258	2.09E-01	-0.006	0.030	8.93E-01
	R_superiortemporal	-0.004	-0.526	5.99E-01	-0.019	0.011	8.05E-01	0.005	0.418	6.76E-01	-0.017	0.026	9.68E-01
	R_supramarginal	0.020	2.912	3.64E-03	0.007	0.033	2.99E-02	0.016	1.669	9.53E-02	-0.003	0.035	8.02E-01
	R_transversetemporal	-0.002	-0.146	8.84E-01	-0.026	0.023	9.08E-01	-0.006	-0.327	7.43E-01	-0.040	0.028	9.82E-01
**Cortical Volume**	L_bankssts	-0.990	-0.051	9.60E-01	-39.233	37.252	9.69E-01	-1.268	-0.047	9.63E-01	-54.625	52.089	9.84E-01
	L_caudalanteriorcingulate	7.738	0.367	7.14E-01	-33.565	49.042	8.51E-01	26.691	0.908	3.64E-01	-30.902	84.284	9.06E-01
	L_caudalmiddlefrontal	-102.105	-2.729	6.42E-03	-175.436	-28.773	4.58E-02	-59.540	-1.142	2.54E-01	-161.704	42.623	9.06E-01
	L_cuneus	-6.565	-0.284	7.77E-01	-51.915	38.785	8.75E-01	-26.668	-0.826	4.09E-01	-89.927	36.591	9.06E-01
	L_entorhinal	-38.291	-2.734	6.33E-03	-65.745	-10.838	4.58E-02	-27.805	-1.421	1.56E-01	-66.163	10.554	8.38E-01
	L_frontalpole	-1.719	-0.400	6.89E-01	-10.144	6.705	8.51E-01	-1.134	-0.189	8.50E-01	-12.896	10.627	9.82E-01
	L_fusiform	-54.890	-1.345	1.79E-01	-134.890	25.109	3.99E-01	4.775	0.084	9.33E-01	-106.208	115.757	9.82E-01
	L_inferiorparietal	-114.798	-1.735	8.29E-02	-244.476	14.879	2.27E-01	-9.757	-0.106	9.16E-01	-190.526	171.012	9.82E-01
	L_inferiortemporal	-100.030	-1.905	5.69E-02	-202.945	2.884	1.79E-01	93.738	1.286	1.99E-01	-49.170	236.645	8.90E-01
	L_insula	-35.714	-1.258	2.08E-01	-91.345	19.917	4.34E-01	-28.080	-0.709	4.79E-01	-105.733	49.572	9.09E-01
	L_isthmuscingulate	5.210	0.333	7.39E-01	-25.428	35.848	8.56E-01	18.169	0.833	4.05E-01	-24.593	60.932	9.06E-01
	L_lateraloccipital	82.442	1.379	1.68E-01	-34.751	199.635	3.87E-01	173.667	2.084	3.73E-02	10.325	337.009	8.02E-01
	L_lateralorbitofrontal	-28.642	-1.043	2.97E-01	-82.487	25.204	5.09E-01	-61.790	-1.619	1.06E-01	-136.589	13.009	8.02E-01
	L_lingual	10.724	0.251	8.02E-01	-72.907	94.355	8.82E-01	21.412	0.360	7.19E-01	-95.233	138.057	9.82E-01
	L_medialorbitofrontal	-29.029	-1.170	2.42E-01	-77.643	19.585	4.65E-01	-35.544	-1.028	3.04E-01	-103.303	32.214	9.06E-01
	L_middletemporal	-0.284	-0.006	9.95E-01	-91.130	90.561	9.95E-01	90.663	1.405	1.60E-01	-35.839	217.165	8.38E-01
	L_paracentral	-14.407	-0.763	4.46E-01	-51.422	22.609	6.67E-01	-18.982	-0.720	4.72E-01	-70.671	32.707	9.09E-01
	L_parahippocampal	-29.311	-2.398	1.66E-02	-53.263	-5.359	8.44E-02	-22.903	-1.343	1.79E-01	-56.328	10.522	8.90E-01
	L_parsopercularis	-21.701	-0.737	4.61E-01	-79.440	36.037	6.76E-01	-17.037	-0.414	6.79E-01	-97.629	63.554	9.68E-01
	L_parsorbitalis	7.871	0.739	4.60E-01	-12.992	28.734	6.76E-01	24.644	1.663	9.66E-02	-4.407	53.695	8.02E-01
	L_parstriangularis	13.612	0.600	5.49E-01	-30.850	58.075	7.62E-01	16.660	0.526	5.99E-01	-45.407	78.727	9.68E-01
	L_pericalcarine	-19.321	-0.840	4.01E-01	-64.393	25.751	6.13E-01	-35.663	-1.112	2.66E-01	-98.524	27.198	9.06E-01
	L_postcentral	-11.236	-0.249	8.03E-01	-99.680	77.208	8.82E-01	65.638	1.046	2.96E-01	-57.401	188.677	9.06E-01
	L_posteriorcingulate	-12.759	-0.675	5.00E-01	-49.808	24.290	7.11E-01	5.310	0.201	8.41E-01	-46.397	57.017	9.82E-01
	L_precentral	-37.570	-0.672	5.02E-01	-147.132	71.993	7.11E-01	17.151	0.221	8.25E-01	-134.938	169.241	9.82E-01
	L_precuneus	21.955	0.479	6.32E-01	-67.827	111.736	8.19E-01	-15.653	-0.245	8.06E-01	-140.876	109.571	9.82E-01
	L_rostralanteriorcingulate	-4.497	-0.238	8.12E-01	-41.495	32.501	8.82E-01	-6.322	-0.240	8.10E-01	-57.930	45.286	9.82E-01
	L_rostralmiddlefrontal	-74.058	-1.070	2.85E-01	-209.759	61.644	5.08E-01	-14.881	-0.154	8.78E-01	-204.252	174.489	9.82E-01
	L_superiorfrontal	-31.779	-0.378	7.05E-01	-196.380	132.822	8.51E-01	36.765	0.315	7.53E-01	-192.018	265.547	9.82E-01
	L_superiorparietal	-81.876	-1.277	2.02E-01	-207.495	43.743	4.27E-01	-113.694	-1.272	2.04E-01	-288.923	61.536	8.90E-01
	L_superiortemporal	-118.365	-2.304	2.14E-02	-219.070	-17.659	9.52E-02	-55.724	-0.780	4.36E-01	-195.755	84.307	9.06E-01
	L_supramarginal	-110.014	-1.703	8.87E-02	-236.598	16.570	2.34E-01	-67.178	-0.745	4.56E-01	-243.862	109.505	9.06E-01
	L_transversetemporal	-11.396	-1.457	1.45E-01	-26.731	3.938	3.58E-01	-11.035	-1.014	3.11E-01	-32.364	10.294	9.06E-01
	R_bankssts	9.328	0.563	5.73E-01	-23.134	41.791	7.87E-01	56.995	2.472	1.35E-02	11.807	102.182	8.02E-01
	R_caudalanteriorcingulate	-7.199	-0.349	7.27E-01	-47.639	33.242	8.51E-01	31.826	1.108	2.68E-01	-24.494	88.146	9.06E-01
	R_caudalmiddlefrontal	-84.771	-2.158	3.11E-02	-161.775	-7.768	1.21E-01	-51.462	-0.940	3.47E-01	-158.719	55.795	9.06E-01
	R_cuneus	-11.539	-0.521	6.02E-01	-54.939	31.861	8.05E-01	-19.920	-0.645	5.19E-01	-80.491	40.650	9.10E-01
	R_entorhinal	-41.007	-3.371	7.67E-04	-64.852	-17.162	1.17E-02	-44.531	-2.622	8.82E-03	-77.818	-11.243	8.02E-01
	R_frontalpole	12.014	2.358	1.85E-02	2.026	22.002	9.21E-02	11.001	1.549	1.22E-01	-2.919	24.921	8.02E-01
	R_fusiform	-49.963	-1.235	2.17E-01	-129.232	29.305	4.38E-01	-9.742	-0.174	8.62E-01	-119.677	100.194	9.82E-01

	R_inferiorparietal	20.566	0.279	7.81E-01	-124.137	165.269	8.75E-01	52.240	0.507	6.12E-01	-149.647	254.126	9.68E-01
	R_inferiortemporal	-86.705	-1.804	7.15E-02	-180.930	7.519	2.17E-01	-10.257	-0.153	8.78E-01	-141.507	120.993	9.82E-01
	R_insula	7.072	0.232	8.16E-01	-52.560	66.704	8.82E-01	-37.297	-0.878	3.80E-01	-120.541	45.948	9.06E-01
	R_isthmuscingulate	6.561	0.467	6.41E-01	-20.978	34.100	8.26E-01	8.304	0.423	6.72E-01	-30.171	46.779	9.68E-01
	R_lateraloccipital	122.933	1.942	5.23E-02	-1.121	246.987	1.75E-01	93.583	1.059	2.90E-01	-79.568	266.733	9.06E-01
	R_lateralorbitofrontal	-40.276	-1.399	1.62E-01	-96.715	16.163	3.78E-01	-8.691	-0.217	8.28E-01	-87.094	69.712	9.82E-01
	R_lingual	42.299	0.929	3.53E-01	-46.963	131.562	5.60E-01	80.740	1.272	2.04E-01	-43.698	205.177	8.90E-01
	R_medialorbitofrontal	-25.326	-1.169	2.43E-01	-67.790	17.139	4.65E-01	-14.931	-0.495	6.21E-01	-74.030	44.167	9.68E-01
	R_middletemporal	52.265	1.151	2.50E-01	-36.759	141.288	4.69E-01	143.246	2.264	2.37E-02	19.220	267.273	8.02E-01
	R_paracentral	8.054	0.365	7.15E-01	-35.223	51.330	8.51E-01	21.725	0.706	4.80E-01	-38.565	82.015	9.09E-01
	R_parahippocampal	-11.775	-1.164	2.44E-01	-31.598	8.047	4.65E-01	7.650	0.544	5.87E-01	-19.936	35.237	9.68E-01
	R_parsopercularis	-31.002	-1.303	1.93E-01	-77.627	15.623	4.16E-01	-12.907	-0.389	6.97E-01	-77.914	52.101	9.82E-01
	R_parsorbitalis	13.133	1.049	2.95E-01	-11.415	37.681	5.08E-01	28.273	1.618	1.06E-01	-5.975	62.520	8.02E-01
	R_parstriangularis	23.733	0.888	3.75E-01	-28.668	76.134	5.85E-01	23.807	0.638	5.23E-01	-49.276	96.890	9.10E-01
	R_pericalcarine	10.337	0.431	6.66E-01	-36.627	57.301	8.39E-01	-8.660	-0.259	7.96E-01	-74.156	56.836	9.82E-01
	R_postcentral	11.553	0.252	8.01E-01	-78.145	101.251	8.82E-01	92.872	1.462	1.44E-01	-31.657	217.400	8.13E-01
	R_posteriorcingulate	-6.678	-0.353	7.24E-01	-43.781	30.426	8.51E-01	23.251	0.882	3.78E-01	-28.409	74.910	9.06E-01
	R_precentral	-58.536	-1.051	2.93E-01	-167.652	50.580	5.08E-01	45.971	0.595	5.52E-01	-105.450	197.391	9.45E-01
	R_precuneus	18.719	0.399	6.90E-01	-73.294	110.732	8.51E-01	5.555	0.085	9.32E-01	-122.775	133.885	9.82E-01
	R_rostralanteriorcingulate	2.498	0.163	8.71E-01	-27.585	32.581	9.05E-01	34.444	1.608	1.08E-01	-7.533	76.421	8.02E-01
	R_rostralmiddlefrontal	22.401	0.303	7.62E-01	-122.280	167.081	8.67E-01	82.614	0.804	4.21E-01	-118.684	283.912	9.06E-01
	R_superiorfrontal	84.775	0.982	3.26E-01	-84.409	253.958	5.29E-01	80.107	0.666	5.05E-01	-155.620	315.834	9.10E-01
	R_superiorparietal	24.198	0.404	6.86E-01	-93.180	141.576	8.51E-01	-3.070	-0.037	9.71E-01	-166.804	160.665	9.84E-01
	R_superiortemporal	-67.700	-1.426	1.54E-01	-160.718	25.319	3.70E-01	47.109	0.713	4.76E-01	-82.320	176.537	9.09E-01
	R_supramarginal	-40.950	-0.743	4.57E-01	-148.945	67.046	6.76E-01	65.132	0.848	3.96E-01	-85.387	215.652	9.06E-01
	R_transversetemporal	0.993	0.182	8.55E-01	-9.692	11.679	8.99E-01	-3.239	-0.426	6.70E-01	-18.149	11.671	9.68E-01
	L_accumb	4.506	1.258	2.09E-01	-2.517	11.529	4.34E-01	0.679	0.136	8.92E-01	-9.125	10.483	9.82E-01
	Lamyg	31.074	3.567	3.72E-04	13.997	48.151	7.62E-03	18.832	1.549	1.22E-01	-4.998	42.662	8.02E-01
	Lcaud	31.382	1.707	8.81E-02	-4.657	67.422	2.34E-01	22.426	0.874	3.82E-01	-27.859	72.711	9.06E-01
	Lhippo	32.410	1.999	4.57E-02	0.640	64.179	1.64E-01	10.654	0.471	6.38E-01	-33.686	54.993	9.68E-01
	LLatVent	-117.874	-0.382	7.03E-01	-722.724	486.975	8.51E-01	-349.862	-0.816	4.15E-01	-1190.34	490.621	9.06E-01
	Lpal	-8.232	-0.942	3.46E-01	-25.351	8.888	5.53E-01	13.350	1.097	2.73E-01	-10.493	37.194	9.06E-01
	Lput	59.341	2.856	4.35E-03	18.613	100.069	3.45E-02	22.715	0.784	4.33E-01	-34.094	79.525	9.06E-01
**Subcortical Volume**	Lthal	56.925	2.267	2.35E-02	7.714	106.136	1.03E-01	66.430	1.896	5.81E-02	-2.235	135.096	8.02E-01
	Raccumb	3.168	0.908	3.64E-01	-3.673	10.009	5.73E-01	-1.251	-0.257	7.97E-01	-10.792	8.289	9.82E-01
	Ramyg	22.201	2.623	8.79E-03	5.613	38.790	5.41E-02	6.187	0.524	6.00E-01	-16.956	29.331	9.68E-01
	Rcaud	42.664	2.244	2.50E-02	5.395	79.933	1.07E-01	28.746	1.084	2.78E-01	-23.218	80.710	9.06E-01
	Rhippo	55.073	3.209	1.36E-03	21.436	88.710	1.73E-02	25.698	1.073	2.83E-01	-21.224	72.620	9.06E-01
	RLatVent	-317.120	-1.228	2.20E-01	-823.263	189.024	4.38E-01	-459.467	-1.282	2.00E-01	-1161.79	242.856	8.90E-01
	Rpal	-3.071	-0.363	7.17E-01	-19.661	13.519	8.51E-01	13.633	1.155	2.48E-01	-9.496	36.762	9.06E-01
	Rput	49.462	2.336	1.96E-02	7.956	90.968	9.52E-02	9.923	0.336	7.37E-01	-47.998	67.844	9.82E-01
	Rthal	97.027	4.353	1.42E-05	53.342	140.712	1.01E-03	74.945	2.408	1.61E-02	13.949	135.941	8.02E-01

References

1. Pereira JB, Mijalkov M, Kakaei E, et al. Disrupted network topology in patients with stable and progressive mild cognitive impairment and Alzheimer's disease. *Cerebral Cortex*. 2016;26(8):3476-93.
2. Armstrong NM, An Y, Doshi J, et al. Association of Midlife Hearing Impairment With Late-Life Temporal Lobe Volume Loss. *JAMA Otolaryngology–Head & Neck Surgery*. 2019;145(9):794. doi:10.1001/jamaoto.2019.1610
3. Chen X, Hu K, Song H, et al. Depression, anxiety and brain volume after hearing loss and tinnitus: cohort study in the UK Biobank. *BJPsych Open*. 2024;10(2):e37. doi:10.1192/bjo.2023.634
4. Profant O, Škoch A, Tintěra J, et al. The Influence of Aging, Hearing, and Tinnitus on the Morphology of Cortical Gray Matter, Amygdala, and Hippocampus. *Front Aging Neurosci*. 2020;12. doi:10.3389/fnagi.2020.553461
5. Wang HF, Zhang W, Rolls ET, et al. Hearing impairment is associated with cognitive decline, brain atrophy and tau pathology. *EBioMedicine*. 2022;86:104336. doi:10.1016/j.ebiom.2022.104336
6. Elmer S, Schmitt R, Giroud N, Meyer M. The neuroanatomical hallmarks of chronic tinnitus in comorbidity with pure-tone hearing loss. *Brain Struct Funct*. 2023;228(6):1511-1534. doi:10.1007/s00429-023-02669-0
7. Lin FR, Ferrucci L, An Y, et al. Association of hearing impairment with brain volume changes in older adults. *Neuroimage*. 2014;90:84-92. doi:10.1016/j.neuroimage.2013.12.059
8. Llano DA, Kwok SS, Devanarayan V, Alzheimer's Disease Neuroimaging Initiative (ADNI). Reported hearing loss in Alzheimer's disease is associated with loss of brainstem and cerebellar volume. *Frontiers in Human Neuroscience*, 2021;15: 739754.
9. Mühlau M, Rauschecker JP, Oestreicher E, et al. Structural Brain Changes in Tinnitus. *Cerebral Cortex*. 2006;16(9):1283-1288. doi:10.1093/cercor/bhj070
10. Oosterloo BC, Croll PH, Goedegebure A, et al. Tinnitus and Its Central Correlates: A Neuroimaging Study in a Large Aging Population. *Ear Hear*. 2021;42(5):1428-1435. doi:10.1097/AUD.0000000000001042
11. Mahoney CJ, Rohrer JD, Goll JC, Fox NC, Rossor MN, Warren JD. Structural neuroanatomy of tinnitus and hyperacusis in semantic dementia. *J Neurol Neurosurg Psychiatry*. 2011;82(11):1274-1278. doi:10.1136/jnnp.2010.235473
